# A direct interaction between CPF and RNA Pol II links RNA 3′ end processing to transcription

**DOI:** 10.1016/j.molcel.2023.11.004

**Published:** 2023-12-21

**Authors:** Manuel Carminati, Juan B. Rodríguez-Molina, M. Cemre Manav, Dom Bellini, Lori A. Passmore

**Affiliations:** 1 MRC Laboratory of Molecular Biology, Cambridge CB2 0QH, UK

**Keywords:** transcription, poly(A) tail, phosphatase, cryo-EM, X-ray crystallography, polymerase, CPSF

## Abstract

Transcription termination by RNA polymerase II (RNA Pol II) is linked to RNA 3′ end processing by the cleavage and polyadenylation factor (CPF or CPSF). CPF contains endonuclease, poly(A) polymerase, and protein phosphatase activities, which cleave and polyadenylate pre-mRNAs and dephosphorylate RNA Pol II to control transcription. Exactly how the RNA 3′ end processing machinery is coupled to transcription remains unclear. Here, we combine *in vitro* reconstitution, structural studies, and genome-wide analyses to show that yeast CPF physically and functionally interacts with RNA Pol II. Surprisingly, CPF-mediated dephosphorylation promotes the formation of an RNA Pol II stalk-to-stalk homodimer *in vitro*. This dimer is compatible with transcription but not with the binding of transcription elongation factors. Disruption of the dimerization interface in cells causes transcription defects, including altered RNA Pol II abundance on protein-coding genes, tRNA genes, and intergenic regions. We hypothesize that RNA Pol II dimerization may provide a mechanistic basis for the allosteric model of transcription termination.

## Highlights

• The yeast 3′ end processing complexes, CPF and APT, directly interact with RNA Pol II
• Ref2 contributes to RNA Pol II binding by CPF/APT and regulates Glc7 (PP1) phosphatase
• Dephosphorylation of RNA Pol II *in vitro* promotes stalk-to-stalk homodimerization
• Disrupting the RNA Pol II dimerization interface *in vivo* causes transcription defects

## Graphical abstract

**Figure:**
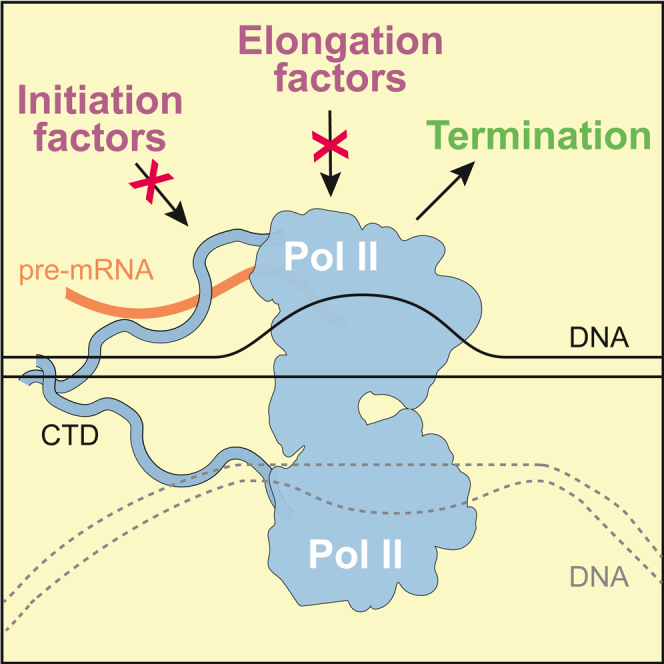

## Teaser

The nature of the coupling between transcription and RNA 3′ end processing has remained elusive. Carminati et al. report that the cleavage and polyadenylation factor (CPF) binds and dephosphorylates RNA Pol II *in vitro*, which promotes RNA Pol II dimerization. Dimerization is incompatible with elongation factor binding, suggesting a potential role in termination.

## Introduction

Eukaryotic mRNAs must be processed before they can be exported from the nucleus and translated into proteins. This includes capping at their 5′ end, splicing, and 3′ cleavage and polyadenylation. Crosstalk between these processes promotes coordinated and timely recruitment of transcription factors, 5′ capping proteins, the spliceosome, and the 3′ end processing machinery to RNA polymerase II (RNA Pol II) and nascent pre-messenger RNA (pre-mRNA).^1^^,^^2^^,^^3^ Together, this enhances the efficiency and accuracy of pre-mRNA maturation.^4^^,^^5^

Transcription and RNA processing are coordinated by the regulated phosphorylation of the C-terminal domain (CTD) of Rpb1, the largest subunit of RNA Pol II. The CTD is composed of 26 heptapeptide repeats in yeast (52 in humans) with a consensus sequence of Y_1_S_2_P_3_T_4_S_5_P_6_S_7_ that is differentially phosphorylated throughout the transcription cycle. The CTD is unphosphorylated when RNA Pol II is loaded onto promoters during transcription initiation.^6^ Phosphorylation of CTD Ser5 (P-Ser5) peaks early in transcription and promotes the recruitment of transcription elongation factors, 5′ capping proteins, splicing factors^7^^,^^8^^,^^9^ and, on short non-coding RNAs, termination factors.^10^ When RNA Pol II engages in productive elongation, P-Ser5 levels decrease and phosphorylation of Tyr1 and Ser2 residues (P-Tyr1 and P-Ser2) accumulates. P-Tyr1 prevents the premature termination of transcription.^11^^,^^12^ After transcription of the polyadenylation signal (PAS), P-Ser2 recruits transcription termination factors.^11^^,^^13^

mRNA 3′ end processing is carried out by the cleavage and polyadenylation factor (CPF in yeast; CPSF in humans). CPF contains 14 subunits (total ∼1 MDa) that are organized into three enzymatic modules.^14^ The nuclease module contains the Ysh1 (CPSF73) endonuclease that cleaves nascent RNA at specific sites.^15^ The polymerase module contains Pap1 (PAP), which adds a poly(A) tail onto the new free 3′ end.^16^^,^^17^ The phosphatase module harbors Ssu72 (SSU72) and Glc7 (the yeast protein phosphatase 1, or PP1). Ssu72 is a P-Ser5 phosphatase that plays roles in transcription elongation^18^ and gene looping,^19^ where the gene promoter and terminator are juxtaposed to facilitate RNA Pol II recycling and regulate transcription directionality.^20^^,^^21^ Glc7 dephosphorylates P-Tyr1 of the CTD to elicit transcription termination.^22^ The human and fission yeast orthologs of Glc7, PP1, and Dis2, respectively, also promote transcription termination through dephosphorylation of the elongation factor Spt5.^23^^,^^24^ Although CPF processes the 3′ ends of protein-coding (pc) transcripts, the related associated with Pta1 (APT) complex is required for the transcription of non-coding small nuclear (sn)RNAs and small nucleolar (sno)RNAs by RNA Pol II in yeast.^25^^,^^26^ APT contains all six subunits of the phosphatase module and an additional subunit, Syc1.

Transcription and mRNA 3′ end processing are intimately coupled.^27^^,^^28^ CPF interacts with the transcription initiation complex at promoters, remains associated with RNA Pol II throughout transcription, and is required for transcription termination.^6^^,^^29^^,^^30^ There are two prevalent, non-exclusive models for transcription termination. First, in the torpedo model, pre-mRNA cleavage by CPF is required to allow access to the 5′-3′ “torpedo” exonuclease that releases RNA Pol II from DNA.^31^^,^^32^ In the allosteric model, transcription of the PAS triggers an undefined change in RNA Pol II that renders it competent for termination.^28^^,^^33^ CPF-mediated dephosphorylation of RNA Pol II may contribute to the allosteric change proposed in the allosteric model.^22^

Despite the coupling between 3′ end processing and transcription, it remains unclear how or whether CPF binds transcribing RNA Pol II. Moreover, it is unknown how RNA Pol II affects 3′ end processing. Here, we report that CPF and APT directly bind RNA Pol II. We show that dephosphorylation promotes the formation of RNA Pol II homodimers *in vitro* that are structurally incompatible with transcription elongation. Mutating the RNA Pol II dimerization interface in yeast leads to a number of transcription defects. Together, this establishes that transcription and 3′ end processing are physically connected and suggests that CPF phosphatase activity may regulate transcription by controlling the oligomerization state of RNA Pol II.

## Results

### CPF and APT interact directly with RNA Pol II

**Figure 1:**
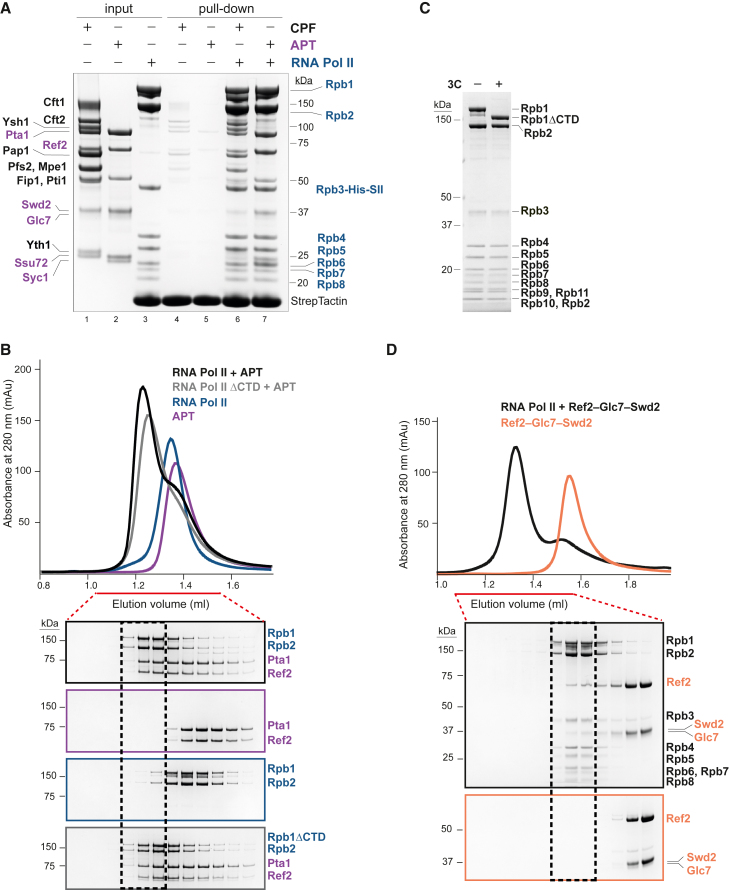
CPF and APT interact directly with RNA Pol II (A) Pull-down assay of CPF or APT using immobilized StrepII (SII)-tagged RNA Pol II (Rpb3-His-SII). Input and bound proteins were analyzed on SDS-PAGE. Labels show APT subunits (purple), core CPF subunits (black), and RNA Pol II (blue). The experiment was repeated twice. (B) (Top) Analytical size exclusion chromatography profiles of RNA Pol II (blue) and APT (purple), loaded separately or after incubation (black). APT with RNA Pol II-ΔCTD is in gray. (Bottom) SDS-PAGE (cropped) of the fractions indicated by a red line. The black dashed box indicates the migration position of the RNA Pol II-APT complex. Gels are outlined according to colors of chromatograms. (C) SDS-PAGE showing 3C protease cleavage of the Rpb1 CTD to make RNA Pol II-ΔCTD. (D) Analytical size exclusion chromatography experiment, as in (B), of RNA Pol II incubated with the Ref2-Glc7-Swd2 subcomplex of APT. See also Figures S1 and S2.

Since transcription and RNA 3′ end processing are functionally coupled,^18^^,^^22^ we tested whether RNA Pol II interacts directly with CPF or APT in a fully defined *in vitro* system. We purified an affinity-tagged RNA Pol II from yeast, as well as recombinant yeast CPF, CPF-core (polymerase and nuclease modules), phosphatase module, and APT (Figure S1A). We then performed CPF and APT pull-down assays using immobilized RNA Pol II. Interestingly, both CPF and APT bound to RNA Pol II *in vitro* (Figure 1A). The phosphatase module of CPF is necessary and sufficient for the interaction (Figure S1B).

As the six subunits of the phosphatase module are also found within APT, we reasoned that both complexes likely interact with RNA Pol II in a similar manner. We therefore focused our subsequent analyses on APT, which is more stable and less prone to degradation than the phosphatase module *in vitro*. APT and RNA Pol II co-eluted on analytical size exclusion chromatography, showing that they form a stable complex in solution (Figure 1B). In summary, these data show that APT and the phosphatase module of CPF directly bind RNA Pol II.

### APT interacts with the core of RNA Pol II

Next, we investigated which region of RNA Pol II interacts with APT. Since the CTD of RNA Pol II is a substrate of APT, it could be involved in their interaction. The CTD is essential for viability in yeast,^34^ and therefore, we used CRISPR-Cas9 to introduce a 3C protease cleavage site into Rpb1 upstream of the CTD. This allowed removal of the CTD after RNA Pol II purification (Figure 1C). We incubated RNA Pol II-ΔCTD with APT and found that they still co-elute on size exclusion chromatography (Figure 1B), demonstrating that they interact even in the absence of the CTD.

Previous data showed that an interaction between Ssu72 and Rpb4 (a subunit of the RNA Pol II stalk) promotes the formation of gene loops in yeast.^20^ We purified a version of RNA Pol II lacking both Rpb4 and Rpb7 stalk subunits (RNA Pol II-Δstalk).^20^^,^^35^ This stalk-less RNA Pol II also retained the ability to interact with APT on size exclusion chromatography (Figure S1C).

Next, to define which region of APT interacts with RNA Pol II, we purified two subcomplexes of APT that we had previously identified by native mass spectrometry (Ref2-Glc7-Swd2 and Pta1-Pti1-Ssu72-Syc1, respectively; Figure S1A).^14^ In a pull-down assay, both the Glc7- and Ssu72-containing subcomplexes interact with RNA Pol II, consistent with multiple interaction sites (Figure S1D). The Glc7-subcomplex pulled down more RNA Pol II; hence, we focused on it. In size exclusion chromatography, the migration position of the Glc7 subcomplex shifts after incubation with RNA Pol II, confirming that they directly interact (Figure 1D).

Together, our data show that neither the CTD nor the stalk are required for APT to bind RNA Pol II. Moreover, we found that the Ref2-Glc7-Swd2 subcomplex makes a major contribution toward the interaction of APT with RNA Pol II, but there are likely also interactions with Pta1-Pti1-Ssu72-Syc1.

### APT binds RNA Pol II next to the RNA exit channel

To gain insights into the functional interplay between RNA Pol II and the 3′ end processing machinery, we tested whether *in vitro* transcription by RNA Pol II is influenced by the presence of APT or CPF in a promoter-independent RNA elongation assay. We made an artificial transcription bubble using a model pre-mRNA substrate, which is elongated by RNA Pol II in the presence of ribonucleotides. This showed that basal transcription by RNA Pol II is compatible with the presence of APT and CPF (Figure S2A).

The RNA Pol II CTD has been proposed to stimulate 3′ cleavage.^1^ We tested this in a fully reconstituted system. We assembled RNA Pol II on a transcription bubble containing a longer version of the model pre-mRNA substrate, mimicking the transcription of an RNA with an upstream cleavage site. We carried out cleavage assays with CPF in the presence of the accessory factors: cleavage factor (CF) IA and CF IB. Our results show that CPF mediates efficient and specific cleavage of pre-mRNA in the presence of RNA Pol II, but its activity is neither stimulated nor inhibited (Figure S2B).

To understand whether there are other functional consequences of physically connecting the RNA 3′ end processing machinery to RNA Pol II, we examined the structure of RNA Pol II in complex with APT by cryoelectron microscopy (cryo-EM). Thus, we assembled a complex between purified APT and RNA Pol II loaded onto a DNA-RNA scaffold, which mimics a transcription elongation bubble. Because APT specifically plays a role in the transcription of non-coding transcripts, we assembled a transcription bubble using a small nucleolar RNA (snR47), which had been previously linked to APT activity^25^ (Figure S2C). Cryo-EM analysis of this complex revealed protein aggregation, possibly due to disassembly and denaturation of the 3′ end processing machinery at the air/water interface.^36^ Thus, we prepared cryo-EM grids after gradient centrifugation coupled to glutaraldehyde crosslinking (GraFix)^37^ to stabilize the complex (Figure S3A).

**Figure 2:**
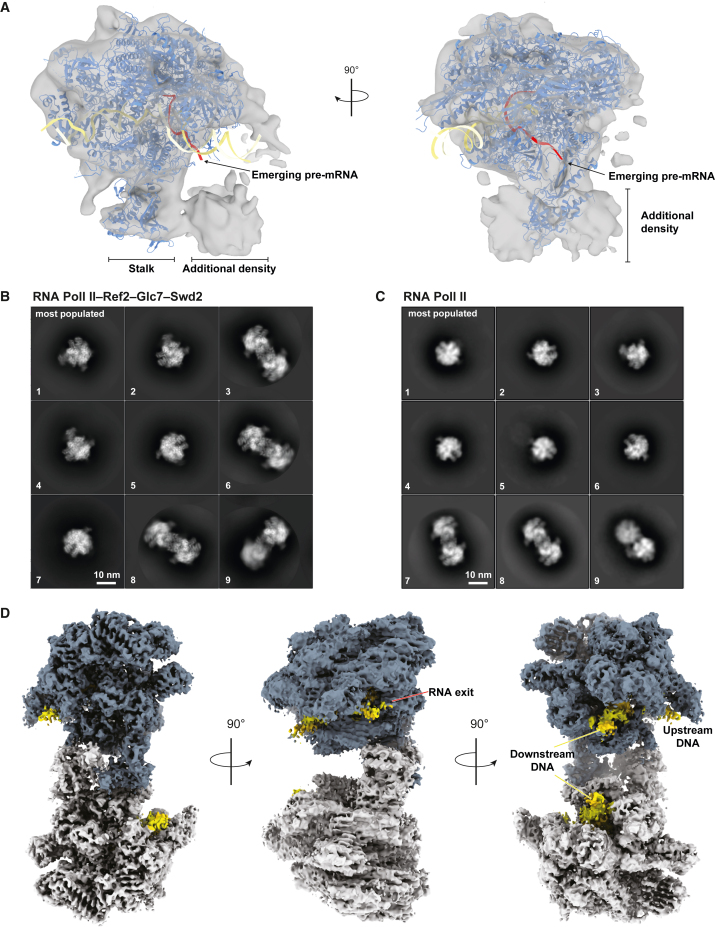
Cryo-EM analysis reveals RNA Pol II homodimers in the presence of Ref2-Glc7-Swd2 (A) 3D reconstruction of RNA Pol II-APT (gray transparent surface) with a model of RNA Pol II (PDB: 5C4X; blue ribbon) rigid-body fit into the map. DNA is in yellow, and RNA is in red. Density not accounted for is indicated. Arrows point at the RNA exit channel. (B and C) Selected 2D class averages of (B) DNA-RNA-loaded RNA Pol II with Ref2-Glc7-Swd2 from 850,000 particles and (C) DNA-RNA-loaded RNA Pol II from 400,000 particles. Classes are ordered by the number of particles in each class, from the most populated (1) to the least populated (9). (D) Composite map of the RNA Pol II dimer after signal subtraction and focused 3D refinement of the individual monomers (global resolution 3.6 Å). Monomers are colored in blue and gray. The DNA-RNA scaffold is in yellow. Anisotropy is due to preferred particle orientation on the grid. See also Figures S3–S6.

3D reconstructions of the transcribing complex show RNA Pol II with additional density next to the RNA exit channel and the stalk (Figures 2A, S3B, and S3C). The additional density is poorly resolved, indicating that APT (and presumably CPF) may be flexibly positioned on RNA Pol II. This binding site of APT is in agreement with the previously reported interaction of Ssu72 with the stalk protein Rpb4.^20^ Together, these data show that the RNA 3′ end processing machinery is positioned on RNA Pol II so that it can monitor the nascent transcript as it emerges from the RNA exit channel. Flexibility would allow it to access RNAs with diverse lengths and structures.

### Ref2-Glc7-Swd2 promotes the formation of RNA Pol II homodimers

To further investigate the association of the 3′ end processing machinery with RNA Pol II, we next assembled the smaller Ref2-Glc7-Swd2 subcomplex of APT with RNA Pol II loaded onto a DNA-RNA hybrid (Figure S4A). We vitrified the sample in native conditions (without crosslinkers) but in the presence of detergents to minimize the orientation bias known to affect RNA Pol II in cryo-EM.^38^^,^^39^^,^^40^ In 2D-class averages, we did not identify any obvious density for Ref2-Glc7-Swd2 (Figures 2B, S4B, and S4C). Unexpectedly, we observed many RNA Pol II homodimers in this dataset. RNA Pol II dimers were also present in the absence of Ref2-Glc7-Swd2, but at a much lower proportion (Figure 2C). Dimers were not evident in our crosslinked samples, but they have been observed in previous studies.^41^ Crosslinking may therefore antagonize dimer formation.

We obtained a 3D map of the RNA Pol II homodimer at a global resolution of 4.6 Å, although the overall map is somewhat anisotropic (Figures S4D and S4E; Table 1). Compared with the structure of RNA Pol II alone, there was no additional density that could account for Ref2-Glc7-Swd2. To further improve the RNA Pol II homodimer map, we carried out focused 3D refinement on each monomer after signal subtraction by masking one monomer at a time (Figures S5 and S6). We then combined the two resulting maps, yielding a composite map of the intact RNA Pol II dimer at an overall resolution of 3.6 Å (Figure 2D; Table 1).

**Table 1:** Cryo-EM data collection and processing

	RNA Pol II dimer (EMDB-15358)	RNA Pol II dimer focused refinement on monomer 1 (EMDB-15359)	RNA Pol II dimer focused refinement on monomer 2 (EMDB-15360)
**Data collection and processing**			

Magnification^a^ UCam	105,000×	105,000×	105,000×
Magnification LMB Krios3	105,000×	105,000×	105,000×
Magnification LMB Krios1	75,000×	75,000×	75,000×
Voltage (kV)	300	300	300
Electron exposure (e^−^/Å^2^)	40	40	40
Defocus range (μm)	1.5–2.7	1.5–2.7	1.5–2.7
Pixel size (Å)^a^ UCam	0.83	0.83	0.83
Pixel size LMB Krios3	0.86	0.86	0.86
Pixel size LMB Krios1	1.04	1.04	1.04
Symmetry imposed	C1	C1	C1
Initial particle images (no.)	5,800,000	5,800,000	5,800,000
Final particle images (no.)	151,000	151,000	151,000
Map resolution (Å)	4.6	3.6	3.6
FSC threshold	0.143	0.143	0.143
Map resolution range (Å)	3.8–17.5	3.3–11.8	3.3–9.0
Map sharpening *B* factor (Å^2^)	−100	−50	−50

a Datasets collected on three different Titan Krios microscopes. Magnification and pixel size refer respectively to K3-Gatan, University of Cambridge; K3-Gatan, Krios III, MRC-LMB; Falcon3, Krios I MRC-LMB.

### RNA Pol II dimerizes via the stalk

**Figure 3:**
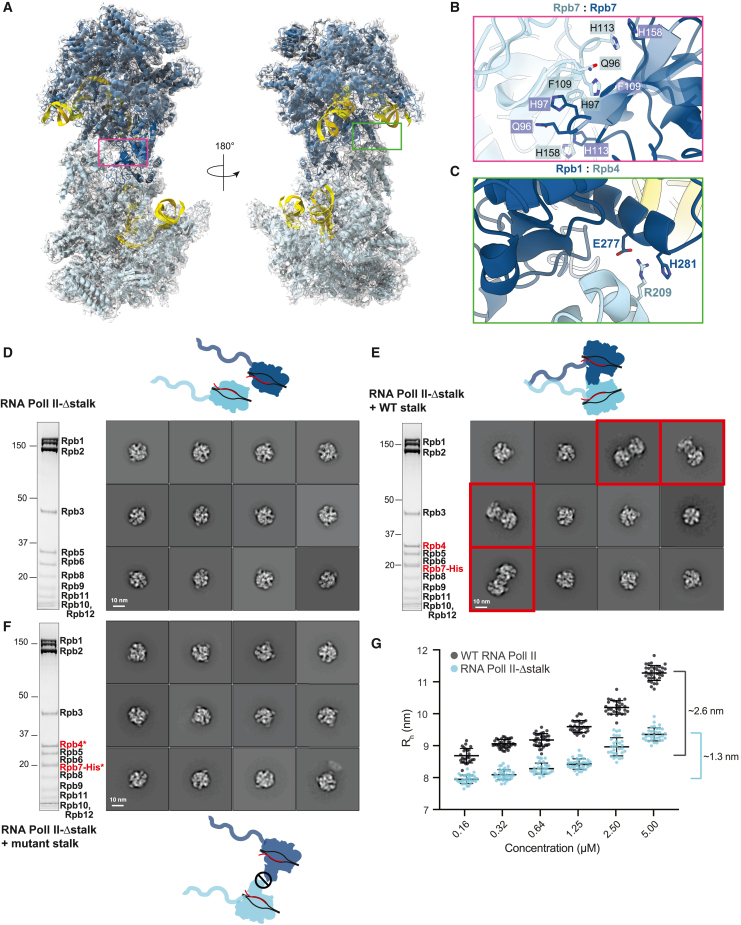
RNA Pol II dimerizes via the stalk protein Rpb7 (A) Two copies of monomeric RNA Pol II rigid-body fit into the dimeric RNA Pol II cryo-EM density. Monomer 1, blue; monomer 2, cyan; DNA, yellow. The magenta and green boxes on the model indicate the close-up views of the RNA Pol II dimer interfaces in (B) and (C), respectively. (B) Hydrophobic interaction between the Rpb7 subunits from each monomer. (C) Interaction between Rpb1 from monomer 1 with Rpb4 from monomer 2. (D–F) SDS-PAGE and cryo-EM analysis of RNA Pol II dimerization. 2D class averages were generated from 400,000 particles for RNA Pol II-Δstalk (D), RNA Pol II-Δstalk with recombinant wild-type (WT) stalk (E), or RNA Pol II-Δstalk with recombinant mutant stalk (F). Selected 2D classes are ordered by the number of particles per class, and dimeric classes are highlighted in red. Asterisks denote mutant stalk proteins. (G) Dynamic light scattering analysis on wild-type (dark gray) or Δstalk RNA Pol II (cyan). The hydrodynamic radius (R_h_) is plotted as a function of RNA Pol II concentration. Black bars represent mean ± SD on 40 acquisitions for each concentration. The brackets on the right represent the increase in R_h_ from the lowest to the highest concentration. See also Figure S7 and Video S1.

We fitted two copies of a crystal structure of monomeric RNA Pol II^42^ into the composite map of the RNA Pol II homodimer (Figure 3A). Overall, these models agree well with the map within the RNA Pol II core, including the DNA-RNA hybrid. There were some deviations between the map and the models for the stalk regions (Figure S7A), suggesting that the stalk is somewhat rearranged upon dimer formation.

The topology of the RNA Pol II dimer is similar to a crystallographic dimer,^42^ with the monomers rotated by ∼15° around the 2-fold symmetry axis (Video S1). RNA Pol II self-associates via two interaction regions that together bury a surface area of ∼2,800 Å^2^ in the crystal structure. This includes a hydrophobic interaction between the Rpb7 stalk subunits of each monomer (Figure 3B) and a polar interaction between Rpb4 of one monomer and Rpb1 of the opposite monomer (Figure 3C). This stalk-to-stalk dimerization interface is adjacent to, but not overlapping with, the putative APT density in the crosslinked RNA Pol II-APT complex (Figure S7B). Thus, crosslinking may stabilize APT binding but destabilize dimerization, and APT binding seems to be structurally compatible with RNA Pol II dimerization. A RNA Pol II dimer in which the cleft is occupied by the stalk of the opposite monomer was previously observed and proposed to be an inactive storage form of RNA Pol II.^41^ In contrast, the stalk-to-stalk dimer is structurally compatible with DNA-RNA loading.

Video S1. Stalk-to-stalk connection in the RNA Pol II dimer, related to Figure 3 Morph movie showing the relationship between the crystallographic Pol II dimer from Barnes et al.,^42^ and the cryoEM Pol II dimer from this study. The two structures are related by a ∼15° rotation along the 2-fold symmetry axis.

To examine whether the interface in the crystallographic dimer is also involved in the dimerization of RNA Pol II in solution, we introduced surface mutations in Rpb7 (Gln96Ala, His97Ala, Phe109Lys, and His158Ala) and Rpb4 (Arg209Ala) (Figures 3B and 3C). We first examined recombinant versions of wild-type (WT) and mutant RNA Pol II stalk alone (Rpb4-Rpb7), expressed and purified from *E. coli*. Size exclusion chromatography coupled with multi-angle light scattering (SEC-MALS) analysis is consistent with the WT stalk being in a monomer-dimer equilibrium. In contrast, the mutant stalk has an average molecular weight less than that of WT Rpb4-Rpb7, suggesting that mutations based on the crystallographic dimerization interface destabilize the dimer in solution (Figure S7C).

To determine the effect of a disrupted stalk-to-stalk interface in the context of RNA Pol II, we incubated DNA-RNA-loaded RNA Pol II-Δstalk with recombinant Rpb4-Rpb7 (WT or mutant) and purified the resultant complex by gel filtration (Figures 3D–3F). We then analyzed these RNA Pol II variants by cryo-EM. We did not observe any dimeric 2D classes with stalk-less RNA Pol II or RNA Pol II reconstituted with a mutant stalk (Figures 3D and 3F). However, RNA Pol II dimerization was rescued after reconstitution with a WT stalk (Figure 3E compared with Figure 2C).

We also performed dynamic light scattering to further examine RNA Pol II behavior in solution. This showed that WT RNA Pol II exhibits a concentration-dependent increase in hydrodynamic radius (R_h_) (Figure 3G), consistent with the concentration-dependent formation of dimers in solution. This increase in R_h_ was reduced for RNA Pol II-Δstalk, suggesting that stalk deletion leads to defects in dimerization. Together, our analysis of RNA Pol II indicates that transcribing RNA Pol II can homodimerize through the stalk.

### Dephosphorylation promotes RNA Pol II dimerization

RNA Pol II dimerization is promoted by Ref2-Glc7-Swd2. However, since Ref2-Glc7-Swd2 was not visible in the cryo-EM map of the RNA Pol II dimer, it likely does not mediate dimerization by physically holding two RNA Pol II monomers together. We therefore tested whether dephosphorylation of RNA Pol II was required for dimerization.

**Figure 4:**
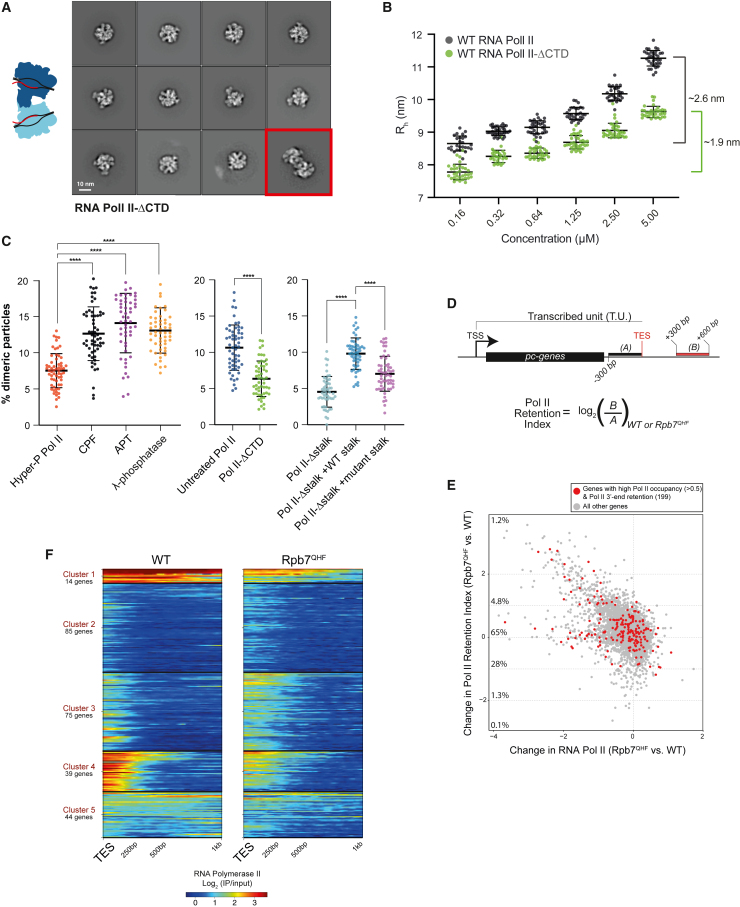
Dephosphorylation promotes RNA Pol II dimerization (A) Selected 2D class averages of RNA Pol II-ΔCTD from 400,000 particles, ordered by the number of particles in each class. A dimeric class is highlighted in red. (B) Dynamic light scattering analysis on wild-type (dark gray; replotted from Figure 3G) or RNA Pol II-ΔCTD (green). Black bars represent mean ± SD on 40 acquisitions for each concentration. The brackets on the right represent the increase in R_h_ between the lowest and highest concentrations for each condition. (C) Analysis of RNA Pol II dimerization by negative stain EM. The percentage of dimeric particles per micrograph is plotted. For each RNA Pol II treatment, 60 (left) or 50 (middle and right) micrographs were acquired. The experiments in the left and middle panels were performed twice, once as a “double-blind” experiment. Black bars represent the mean ± SD. Pairwise comparisons are shown as indicated, where ^∗∗∗∗^p < 0.0001 by one-way ANOVA Tukey’s test. (D) Schematic of regions used to calculate the RNA Pol II retention index. TSS, transcription start site; TES, transcription end site; pc, protein-coding. (E) Scatter plot of the log_2_ fold change in RNA Pol II retention index versus the log_2_-fold change in RNA Pol II signal across the transcribed unit for genes with annotated transcription start and end sites (5,357 genes). Data points highlighted in red correspond to genes with RNA Pol II retention at the 3′ end (clusters 2–4 from the subset of data analyzed in F). The percentage of genes within each retention index bracket is shown. (F) k-means clustering and heatmap of RNA Pol II occupancy beyond the transcription end site (TES) at mRNA genes with RNA Pol II signal ≥0.5 in both wild-type (WT) and Rpb7^QHF^ cells. Clusters 2–4 show RNA Pol II retention in Rpb7^QHF^ cells compared with WT. See also Figures S8 and S9.

First, we determined whether the CTD plays a role in dimerization. We performed cryo-EM followed by 2D classification on RNA Pol II-ΔCTD loaded with the DNA-RNA hybrid. This showed that there was a reduced frequency of RNA Pol II-ΔCTD dimers compared with WT RNA Pol II (Figure 4A compared with Figure 2C). In agreement with this, RNA Pol II-ΔCTD behaved similarly to the dimerization-defective RNA Pol II-Δstalk in dynamic light scattering (Figure 4B compared with Figure 3G). Together, these data suggest that the CTD promotes RNA Pol II dimerization.

To determine whether the phosphorylation state of RNA Pol II can modulate the extent of RNA Pol II dimerization, we estimated the percentage of dimers under different conditions using negative stain EM. We hyperphosphorylated RNA Pol II with Erk2 kinase, incubated it with CPF, APT, or λ phosphatase, and then imaged it (Figures S8A–S8C). We determined the percentage of dimers per micrograph in each condition and calculated the mean dimerization frequencies to allow pairwise comparison (Figure 4C). This showed that CPF- and APT-treated RNA Pol II had an increased frequency of dimerization compared with hyperphosphorylated RNA Pol II. Treatment with λ phosphatase also resulted in an increased number of dimers. Thus, RNA Pol II dimerization is enhanced by phosphatase activity rather than the binding of CPF or APT per se.

We also calculated the proportion of dimers for RNA Pol II-ΔCTD and RNA Pol II-Δstalk and found that the dimerization frequency of both was decreased compared with WT, untreated RNA Pol II (which has a mixed phosphorylation status) (Figure 4C). In agreement with the cryo-EM data, RNA Pol II-Δstalk complemented with WT stalk has a higher proportion of dimers than RNA Pol II with mutant stalk or RNA Pol II-Δstalk (Figure 4C, right). Overall, these data show that dephosphorylated RNA Pol II has an increased dimerization frequency compared with phosphorylated RNA Pol II, and efficient dimerization requires the RNA Pol II CTD and stalk.

We also analyzed RNA Pol II in the absence of a nucleic acid scaffold as a control and found that it can form the stalk-to-stalk dimer, but it also dimerizes through a second interface (a cleft-to-cleft interaction) (Figure S8D). Since DNA occupies the cleft during transcription, the cleft-to-cleft interaction would not be possible in transcribing RNA Pol II. Interestingly, we also observed higher-order oligomers in the absence of a nucleic acid scaffold, which are formed by a combination of the stalk-to-stalk and cleft-to-cleft interactions (Figure S8D). Thus, RNA Pol II can dimerize via at least two distinct mechanisms, and each of these may have different functional implications.

Taken together, our findings indicate that stalk-to-stalk RNA Pol II dimerization is enhanced by dephosphorylation, raising the interesting hypothesis that RNA Pol II phosphorylation state might regulate transcription by modulating RNA Pol II dimerization.

### Transcription is deregulated in a dimerization mutant

To test the impact of disrupting the stalk-to-stalk RNA Pol II dimerization interface on transcription *in vivo*, we generated a yeast strain carrying three-point mutations in Rpb7 (Rpb7^QHF^; Gln96Ala, His97Ala, and Phe109Lys), which disrupt the formation of stalk dimers *in vitro* (Figure S7C). Rpb7^QHF^ yeast cells show a slow growth phenotype compared with WT cells (Figure S9A), demonstrating the importance of this interface.

To determine if Rpb7^QHF^ has an impact on transcription, we measured RNA Pol II occupancy and RNA abundance for the *NCW1* gene by chromatin immunoprecipitation (ChIP)-qPCR and RT-qPCR, respectively (Figures S9B–S9D). This showed that Rpb7^QHF^ causes a retention of RNA Pol II downstream of the canonical termination site, but curiously, we did not detect a corresponding readthrough transcript.

To determine if RNA Pol II retention at the 3′ ends of genes is widespread, we performed genome-wide RNA Pol II ChIP-seq in WT and Rpb7^QHF^ cells (Figure S9E). We also performed 4-thiouracil (4tU)-seq to quantify nascent RNA and detect any readthrough transcripts.^43^ We calculated a RNA Pol II retention index as the normalized RNA Pol II occupancy 300 bp downstream of the transcription end site (TES) (Figures 4D and 4E). We plotted the change in RNA Pol II retention index against the change in RNA Pol II occupancy across the transcription unit for all genes with annotated transcription start and end sites (5,357 genes). Most genes (66%) show a decrease in RNA Pol II occupancy across the transcription unit in Rpb7^QHF^ cells. Curiously, on tRNA genes, RNA Pol II occupancy was increased (Figure S9F). In Rpb7^QHF^ cells, most genes (70.5%) show an increase in RNA Pol II retention index compared with WT, and a small number of genes (6%) show a ≥2-fold increase in the RNA Pol II retention index. Thus, mutation of the dimerization interface leads to major transcriptional defects.

We performed k-means clustering of the RNA Pol II signal within a 1 kb window downstream of the TES. Because most genes showed a general loss of RNA Pol II occupancy, we focused on genes with high RNA Pol II signal (≥0.5 average over the transcribed unit) in both WT and Rpb7^QHF^ cells (257 genes) (Figure 4F). Clusters 2–4 (199 genes; 77% of genes analyzed) show RNA Pol II retention, which is reflected in their RNA Pol II retention index (Figure 4E) and a metagene plot of the average RNA Pol II signal at these genes (Figure S9G). Interestingly, we did not observe readthrough transcripts for these genes (or more generally for genes across the genome), suggesting that 3′ end processing is not affected in Rpb7^QHF^ cells (Figure S9H).

Together, these data show that mutation of the dimerization interface in Rpb7 alters transcription across the yeast genome, including: (1) reduced RNA Pol II occupancy on pc genes; (2) increased RNA Pol II occupancy on tRNA genes; and (3) a retention of RNA Pol II on chromatin beyond the canonical termination site on some pc genes without an obvious readthrough transcript. This suggests that the RNA Pol II dimerization interface is important for normal transcription. It may also contribute to transcription termination and RNA Pol II release without affecting mRNA 3′ end processing.

### Ref2 binds Glc7 and acts as a PP1 regulator

Since the Ref2-Glc7-Swd2 subcomplex of APT binds and dephosphorylates RNA Pol II *in vitro*, we investigated it further. PP1 phosphatases (including Glc7) function with a regulatory subunit that imparts substrate specificity.^44^ Regulatory subunits often interact with PP1 via an RVxF peptide motif. Additional interaction motifs are also commonly found in the regulatory subunits; for example, the ΦΦ motif includes a pair of hydrophobic residues that bind PP1.^45^ The C-terminal region of Ref2 bears a non-canonical RVxF motif (SIKF in Ref2) that has been implicated in Glc7 regulation *in vivo*.^26^

**Figure 5:**
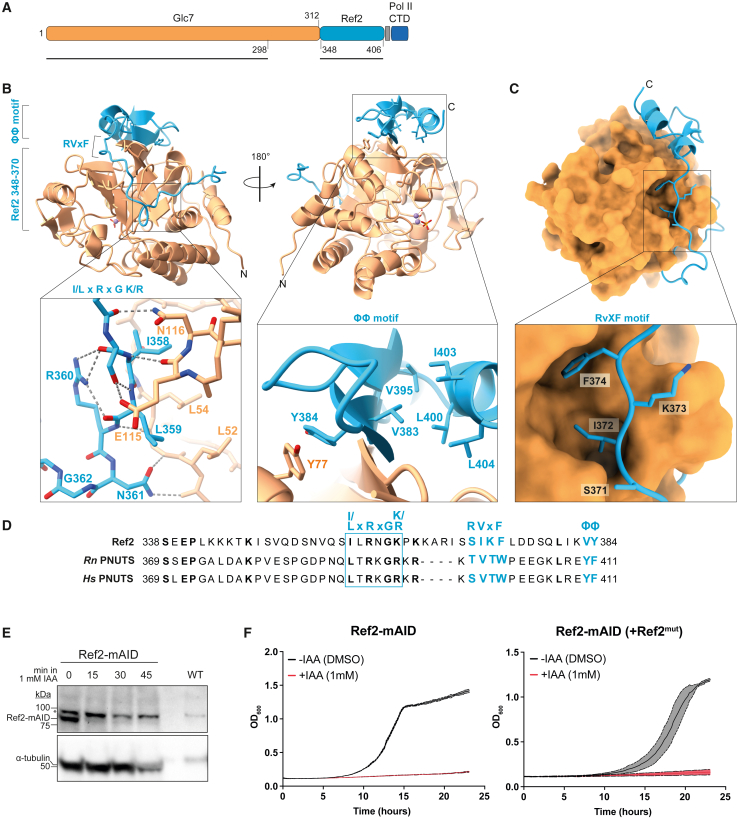
The Ref2 subunit of CPF and APT is a regulatory subunit of Glc7 (A) Schematic of the Glc7-Ref2_348–406_ chimeric protein used for crystallization. Orange, Glc7; blue, Ref2_348–406_; gray, glycine-serine linker; dark blue, two SPTYSPS RNA Pol II CTD repeats. Black lines indicate the regions visible in the crystal structure. (B) Cartoon representation of the Glc7-Ref2_348–406_ crystal structure in two orientations and close-up views of the interactions of Ref2 residues 358–362 (left) and of the intermolecular β sheet formed between Glc7 and Ref2, including interaction details for the hydrophobic pair (ΦΦ) (right). Two manganese ions and a phosphate group are shown in ball-and-stick. Coordination waters are in red. The N and C termini are indicated. (C) View of the interaction interface between Ref2_348–406_ in cartoon and Glc7 in surface representation. The inset shows how Ref2 binds Glc7 through the conserved “RVxF” motif. (D) Sequence alignment of yeast Ref2 and putative orthologs from *Rattus norvegicus* (*Rn*) and *Homo sapiens* (*Hs*). The Ref2 “I/L-x-R-x-G-K/R” motif is enclosed in a light blue box. The RVxF and downstream ΦΦ motifs shared among PP1-regulators are highlighted in light blue. (E) Immunoblots (top, anti-FLAG; bottom, anti-α-tubulin) of Ref2-mAID cells before or after addition of 1 mM auxin (IAA). Ref2-mAID is degraded within 15 min of adding auxin. The anti-FLAG antibody cross-reacts with a protein (^∗^) also present in the wild-type parent strain (final lane). n = 3. (F) Growth curves of Ref2-mAID (left) or Ref2-mAID cells co-expressing a triple-point mutant of Ref2 (Ref2^mut^: I372D, F374K, Y384E). Cells were grown with 1 mM auxin (IAA) or an equivalent volume of DMSO. Dotted line, mean OD_600_ of biological replicates (n = 3); shaded area, standard deviation of the mean. See also Figure S10.

To examine the interaction between Ref2 and Glc7 *in vitro*, we generated C-terminal Ref2 truncations and tested for co-migration with Glc7 in gel filtration (Figure S10A). The smallest truncation we tested comprised residues 348–406 of Ref2 (Ref2_348–406_), and we found that this interacted with Glc7. To understand the molecular basis of their association, we purified the Glc7-Ref2_348–406_ complex, but this was refractory to crystallization. Therefore, we made a chimeric protein where the Ref2 fragment is fused to the flexible C-terminal tail of Glc7 (Figure 5A). We also included two repeats of the RNA Pol II CTD in the chimeric protein with the aim of understanding how the substrate is bound. We crystallized the chimeric protein and determined its structure at 1.85 Å resolution (Figures 5B and S10B; Table 2). All residues of Glc7 and Ref2 could be modeled, except the last 14 residues of the flexible C terminus of Glc7. The CTD substrate was not visible.

**Table 2:** Data collection and refinement statistics (molecular replacement)

	Glc7-Ref2_348–406_
**Data collection**	

Space group	P 31 2 1

**Cell dimensions**	

*a*, *b*, *c* (Å)	90.23, 90.23, 101.14
α, β, γ (°)	90, 90, 120
Total reflections	411,950 (40,121)^a^
Unique reflections	41,059 (4,028)
Resolution (Å)	45.12–1.85 (1.917–1.85)
I/σI	20.46 (1.84)
CC1/2	1 (0.9)
Completeness (%)	99.96 (98.82)
Redundancy	10.0 (10.0)
Rmerge	0.04671 (1.224)

**Refinement**	

Resolution (Å)	45.12–1.85
No. reflections in working set	40,978 (4,015)
No. reflections in working set	2,090 (201)
Rwork/Rfree	0.1920/0.2205
No. atoms	2,987
Protein	2,859
Ligand/ion	3
Water	121

**B-factors**	

Protein	54.64
Ligand/ion	37.78
Water	54.99

**Ramachandran**	

Favored (%)	95.43
Allowed (%)	4.29
Outliers (%)	0.29

**RMSDs**	

Bond lengths (Å)	0.017
Bond angles (°)	1.60

a Values in parentheses are for highest-resolution shell.

The structure shows that Ref2 wraps around the back of Glc7, burying a surface area of ∼4,200 Å^2^. The catalytic site on the opposite side of Glc7 is occupied by two manganese ions and one phosphate ion, as in the recently reported structures of apo-Glc7 (PDB: 7QWJ) and the Phactr1/PP1 holoenzyme.^45^ The absence of density for the RNA Pol II CTD in the active site could indicate weak binding affinity, crystal packing constraints, or a requirement for CTD modifications such as phosphorylation or proline isomerization.^18^^,^^46^

The extensive interaction between Ref2 and Glc7 encompasses three regions of Ref2 (Figures 5B and 5C). First, an N-terminal low-complexity region (348–370) of Ref2 buries a surface area of ∼2,000 Å^2^ through polar and hydrophobic contacts with Glc7 (Figure 5B, left). Sequence alignment with putative Ref2 orthologs shows that many of the interacting residues within this region are conserved (I/L-x-R-x-G-K/R) (Figure 5D). The other two Ref2-Glc7 interactions in the crystal structure are more generally conserved among PP1 regulatory subunits.^44^^,^^45^ This includes the non-canonical RVxF motif (SIKF in Ref2), where Ile372 and Phe374 insert into a hydrophobic pocket on Glc7 (Figure 5C). Consistent with this, Phe374 was previously reported to be required for Glc7 incorporation into the APT complex.^26^ Finally, the ΦΦ motif that is found in many PP1 regulatory subunits is formed of a pair of hydrophobic residues in Ref2, Val383, and Tyr384 (Figure 5B, right). Tyr384 stabilizes the formation of an intermolecular β sheet via a π-π stacking interaction with Tyr77 of Glc7. Val383 inserts into a hydrophobic groove that includes residues 396–406 of Ref2, which form a C-terminal α helix that folds back onto the Glc7-Ref2 β sheet.

Superposition of the Glc7-Ref2_348–406_ crystal structure onto apo-Glc7 (Cα root-mean-square deviation [RMSD] 0.47 Å) (PDB: 7QWJ) or apo-PP1 (Cα RMSD 0.52 Å) (PDB: 4MOV) indicates that Ref2 does not induce major conformational changes within Glc7 (Figure S10C). This is consistent with the mechanism of PP1 regulatory subunits, which often play roles in substrate recruitment, specificity, and localization rather than as allosteric factors.^44^ In agreement with this, the *in vitro* dephosphorylation efficiency of hyperphosphorylated RNA Pol II is similar for Glc7 and Glc7-Ref2_348–406_ (Figure S10D). Taken together, these findings suggest that Ref2 is a PP1 regulatory subunit.

To test the physiological impact of the Ref2-Glc7 interface, we constructed a yeast strain with a mini auxin-induced degron (mAID) fused to the C-terminal end of Ref2 (Ref2-mAID; Figure 5E).^47^ Cells with a deleted *REF2* gene have been reported to be viable^48^; hence, we were surprised to find that depletion of Ref2-mAID completely arrested cell growth (Figures 5F, left and S10E, left). Therefore, *REF2* is essential for viability in this genetic background.

We next built a construct with a Ref2 variant carrying point mutations at the Ref2-Glc7 interaction interface (Ref2^mut^; Ile372Asp, Phe374Lys, and Tyr384Glu). Co-expression of Ref2^mut^ was unable to rescue the growth defect of Ref2-mAID cells in the presence of auxin, but WT Ref2 did (Figures 5F and S10E). These data suggest an essential role for the Ref2-Glc7 interaction.

### The Ref2 subunit of CPF and APT contributes to the interaction with RNA Pol II

**Figure 6:**
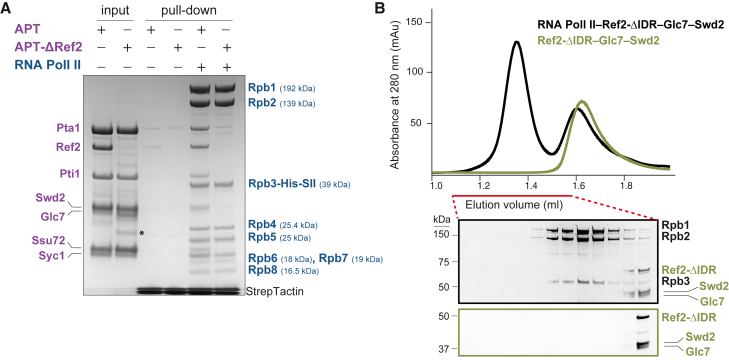
Ref2 is required for the APT interaction with RNA Pol II (A) Pull-down assay of APT and APT-ΔRef2 with RNA Pol II immobilized on StrepTactin beads. SII, StrepII-tagged protein; APT subunits, purple; RNA Pol II subunits, blue; asterisk, degradation product. APT-ΔRef2 was obtained after Ref2 was degraded by contaminating proteases during purification of APT. (B) Analytical size exclusion chromatography of RNA Pol II and Ref2-ΔIDR-Glc7-Swd2. The fractions indicated by a red line were analyzed on the SDS-PAGE below. See also Figure S11.

Many RNA Pol II interactions are mediated by intrinsically disordered regions (IDRs).^49^ Ref2 and its putative mammalian ortholog PNUTS^23^^,^^50^^,^^51^ are predicted to be largely disordered by AlphaFold2,^52^^,^^53^ except for their TFIIS N-terminal domain (TND), which is a structured interaction module found in transcription elongation factors (Figure S11A).^54^ To test whether Ref2 is required for the interaction between APT and RNA Pol II, we performed a pull-down assay using WT APT or APT lacking Ref2, and immobilized RNA Pol II. This revealed that the Ref2-containing version of APT, but not APT-ΔRef2, interacts with RNA Pol II (Figure 6A). Thus, Ref2 is required for the interaction of APT (and presumably CPF) with RNA Pol II.

Next, we sought to determine which region of Ref2 is responsible for the interaction with RNA Pol II. Given the role of disordered regions in transcription regulation,^49^^,^^54^ we generated a Ref2-ΔIDR-Glc7-Swd2 complex where the IDR of Ref2 was removed. Size exclusion chromatography showed that the IDR of Ref2 is essential for the interaction of Ref2-Glc7-Swd2 with RNA Pol II (Figure 6B). However, the interaction of APT with RNA Pol II was not fully abrogated with Ref2-ΔIDR (Figure S11B), suggesting that APT and Ref2 might make multiple contacts with RNA Pol II. Overall, these data show that Ref2 connects the Glc7 phosphatase subunit of CPF and APT to RNA Pol II, thus creating a physical link between RNA 3′ end processing and transcription regulation.

## Discussion

Here, we describe physical and functional connections between the RNA 3′ end processing and transcription machineries. We show that dephosphorylation promotes stalk-to-stalk RNA Pol II dimerization *in vitro*, which is compatible with transcribing RNA Pol II. In cells, the RNA Pol II dimerization interface is important for general transcription and the release of RNA Pol II from some genes. Given that CPF-mediated dephosphorylation is known to promote termination in yeast and humans,^22^^,^^23^^,^^24^^,^^26^ we hypothesize that transcription of the PAS leads to the activation of CPF endonuclease and phosphatase activities, promoting RNA Pol II dimerization and termination.

We show that the Ref2 subunit of CPF and APT is required for the interaction with both RNA Pol II and Glc7; it acts as a PP1 regulatory subunit, and it likely targets Glc7 phosphatase activity. A region within the Ref2 IDR has also been reported to bind RNA,^55^ whereas the TND may mediate crosstalk between transcription factors.^54^ Thus, Ref2 might act as a hub, bringing together the transcription machinery, the 3′ end processing machinery, transcription factors, and RNA. In humans and fission yeast, Ref2 and Glc7 orthologs also play roles in transcription termination,^23^^,^^24^ and therefore, the function of Ref2 is likely to be conserved.

CPF is thought to associate with RNA Pol II and TFIID at promoters^6^ and remain associated with RNA Pol II throughout transcription.^25^ However, it has remained unclear if CPF interacts with RNA Pol II directly. We show that CPF and APT contact core regions of RNA Pol II, and the CTD is dispensable for their interaction. In contrast, CF IA and many transcription factors are recruited by the CTD. For example, within the CF IA complex, the Pcf11 subunit recognizes P-Ser2 of the CTD at the PAS.^13^^,^^56^ Interestingly, although the CTD is not required for the interaction of the 3′ end processing machinery with RNA Pol II, it is required for efficient RNA Pol II dimerization. Oligomerization of dephosphorylated CTDs could increase the local concentration of RNA Pol II to promote the stalk-to-stalk interaction.

**Figure 7:**
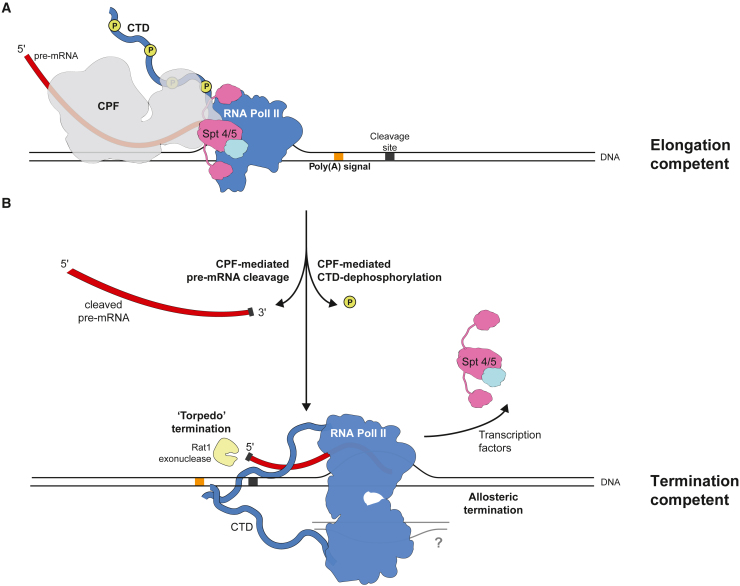
Proposed model for the role of CPF in transcription termination (A) CPF and APT can bind elongating RNA Pol II to monitor RNA as it emerges from the RNA exit channel. PAS sequence, orange box; cleavage site, gray box; nascent RNA, dark red; yellow circles, phosphorylation. Transcription elongation factors Spt4/5 are also shown. (B) After the PAS is transcribed, CPF-mediated pre-mRNA cleavage and RNA Pol II CTD-dephosphorylation are activated. As a result, the newly exposed 5′ end still attached to RNA Pol II is degraded by the torpedo exonuclease Rat1 (left), and RNA Pol II dephosphorylation triggers an allosteric event (RNA Pol II dimerization), which displaces transcription factors, or domains of transcription factors (right). Thus, CPF may convert RNA Pol II into a termination-competent complex. It remains unclear whether the second RNA Pol II is also transcribing. See also Figure S12.

The stalk-to-stalk dimer forms *in vitro* on dephosphorylated RNA Pol II loaded onto a DNA-RNA transcription bubble, and therefore, CPF/APT could promote formation of the stalk-to-stalk dimer while RNA Pol II is actively transcribing. Future work will be required to define whether dimers form between two transcribing RNA Pol II molecules in cells. Association of APT near the RNA exit channel would allow it to monitor the emerging RNA, as it is transcribed by RNA Pol II (Figure 7A). The RNA capping enzyme complex^57^^,^^58^ and the U1 snRNP splicing complex^59^ also bind close to the RNA exit tunnel, but they all contact different parts of RNA Pol II. Thus, our study shows that co-transcriptional RNA-processing factors use a common mechanism of associating with transcribing RNA Pol II near the RNA exit channel, but they achieve this through different contacts.

We propose that RNA Pol II dimerization could be the structural change in RNA Pol II that is postulated in the “allosteric” model of transcription termination. The stalk-to-stalk RNA Pol II dimer is not compatible with transcription initiation or elongation, as there are major clashes with pre-initiation and elongation factors (Figures S12A and S12B). Moreover, an intact stalk-to-stalk dimer interface is required for complete transcription termination at some mRNA genes (Figure S9G).

Thus, in a revised model of transcription termination, the 3′ end processing machinery recognizes the cleavage site after transcription of the PAS. This could activate Glc7 within CPF (or within APT on snRNAs/snoRNAs) to dephosphorylate RNA Pol II. Dephosphorylation promotes RNA Pol II dimerization (the allosteric change), which is incompatible with elongation factor binding. Thus, this would render RNA Pol II competent for transcription termination (Figure 7B).

After termination, multiple modes of RNA Pol II dimerization (stalk-to-stalk and cleft-to-cleft) could contribute to the assembly of higher-order oligomers (Figure S8D), consistent with clustering of hypophosphorylated RNA Pol II.^60^^,^^61^^,^^62^^,^^63^ In support of this possibility, DNA-RNA-loaded RNA Pol II can undergo droplet formation in the presence of APT *in vitro* (Figure S12C).

In summary, transcription of the RNA signals recognized by the 3′ end processing machinery triggers a series of events that inhibit transcription elongation and promote transcription termination. A complex interplay between the nascent RNA, RNA Pol II, and the 3′ end processing machinery suggests that the regulation of transcription by RNA processing is likely underappreciated.

### Limitations of the study

Although Ref2-Glc7 (and PNUTS-PP1) are known to play a role in termination, we cannot exclude the possibility that another phosphatase promotes the RNA Pol II stalk-to-stalk dimer *in vivo*. For example, the Ssu72 phosphatase of CPF/APT is known to play a role in gene looping.^20^^,^^21^ In addition, although we investigated the Ref2-Glc7-Swd2 complex more thoroughly, the Pti1-Pta1-Ssu72-Syc1 subcomplex may also contribute to the interaction with RNA Pol II.

There are major transcriptional defects in the RNA Pol II dimerization mutant. We observe the accumulation of RNA Pol II downstream of a subset of genes in the dimerization interface mutant. However, transcription termination was difficult to evaluate on most genes because of the widespread decrease in RNA Pol II occupancy across most transcription units. RNA Pol II occupancy on pc genes may be generally reduced because transcription units are poorly defined when termination is defective, consistent with an increase in RNA Pol II occupancy in intergenic regions and on tRNA genes.

The RNA Pol II stalk-to-stalk dimer could also be involved in gene looping and polymerase recycling, possibly mediating contact between gene promoter and terminator. The dimerization surface could also be involved in other transcription processes, including initiation and capping, complicating the interpretation of our Rpb7^QHF^ mutant. Specifically, it is possible that the three-point mutations in the Rpb7^QHF^ strain also affect TFIIE binding. However, a TFIIE mutant affects transcription differently to mutations in the dimerization interface.^64^ Finally, the origin of the second RNA Pol II remains unclear. It could be at the promoter, it could be free RNA Pol II, or it could originate from somewhere else.

## STAR★Methods

### Key resources table

**Table:** 

REAGENT or RESOURCE	SOURCE	IDENTIFIER
**Antibodies**		

Anti-RNA Pol II Rpb3 (clone 1Y26)	Abcam	Cat# ab202893 RRID:AB_1129174
Anti-RNA Pol II pTyr1 (clone 3D12)	Antibody Core Facility - Helmholtz-Zentrum Munich	N/A
Anti-RNA Pol II pSer5 (clone 3E8)	Antibody Core Facility - Helmholtz-Zentrum Munich	N/A
Goat anti-mouse IgG DyLight 800	ThermoFisher	Cat# SA5-10176; RRID:AB_2556756
Goat anti-rat IgG DyLight 800	ThermoFisher	Cat# SA5-10024; RRID:AB_2556604
Anti-FLAG M2	Sigma	F1804; RRID:AB_262044
Anti-alpha tubulin	Abcam	ab184970; RRID:AB_2928998

**Bacterial and virus strains**		

*E. coli* DH10 EMBacY	Geneva Biotech	N/A
*E. coli* TOP10	ThermoFisher	Cat# C404010
BL21(DE3) Chemically Competent Cells	Merck	CMC0014
Rosetta 2(DE3)pLysS Competent Cells	Merck (Novagen)	71403-M

**Chemicals, peptides, and recombinant proteins**		

Insect-XPRESS protein-free insect cell medium with L-glutamine	Lonza	12-730Q
Strep-Tactin resin	IBA-Lifesciences	Cat# 2-1201-025
Desthiobiotin	IBA-Lifesciences	Cat# 2-1000-005
Protease inhibitor tablets	Roche	Cat# 11836153001
DnaseI (Rnase free)	New England Biolabs	Cat# M0303S
ECL Prime Western Blotting Detection Reagent	Cytiva	Cat# RPN2236
Imidazole	Sigma-Aldrich	Cat# I5513
TWEEN® 20	Sigma-Aldrich	Cat# P1379
Glutaraldehyde solution	Sigma-Aldrich	Cat# G5882
UltraPure Salmon sperm DNA solution	Invitrogen	Cat# 15632011
1,4-Butanediol, 99%	Thermo Scientific™	Cat# 11382176
5-FOA	Zymo Research	Cat# F9001-5
Recombinant protein: *S. cerevisiae* CPF phosphatase module (Ref2-SII)	Kumar et al.^65^	N/A
Recombinant protein: *S. cerevisiae* CPF (Ref2-3C-SII, Pfs2-3C-SII)	Kumar et al.^65^	N/A
Recombinant protein: *S. cerevisiae* APT (Ref2-3C-SII)	This study	N/A
Recombinant protein: *S. cerevisiae* APTΔRef2	This study	N/A
Recombinant protein: *S. cerevisiae* Ref2-TEV-SII–Glc7–Swd2	This study	N/A
Recombinant protein: *S. cerevisiae* Ref2ΔIDR-TEV-SII–Glc7–Swd2	This study	N/A
Recombinant protein: *S. cerevisiae* Pta1-TEV-SII–Pti1–Ssu72–Syc1	This study	N/A
Recombinant protein: *S. cerevisiae* Rpb4–Rpb7-3C-His_6_	This study	N/A
Recombinant protein: *S. cerevisiae* Rpb4–Rpb7-3C-His_6_ Q96A, H97A, F109K, H158A (Rpb7), R209A (Rpb4)	This study	N/A
Recombinant protein: *S. cerevisiae* His_10_-SUMO-Glc7–Ref_2348-406_–GSGSG–SPSYSPTSPSYSPT	This study	N/A
Recombinant protein: *S. cerevisiae* His_10_-SUMO-Glc7	PDB: 7QWJ	N/A
Recombinant protein: *S. cerevisiae* His_10_-SUMO-Ref2_348-523_	This study	N/A
Recombinant protein: *S. cerevisiae* GST-3C–Ref2_348-434_	This study	N/A
Recombinant protein: *S. cerevisiae* GST-3C–Ref2_348-406_	This study	N/A
Recombinant protein: *S. cerevisiae* CF IA	Kumar et al.^65^	N/A
Recombinant protein: *S. cerevisiae* CF IB	Hill et al.^36^	N/A
Auxin (3-Indoleacetic acid)	Sigma	Cat# I3750-100G-A
4-thiouracil	Sigma	Cat# 440736–1G
TRI reagent	Thermo Scientific™	Cat# AM9738
DnaseI (Rnase free)	New England Biolabs	Cat# M030S
EZ-Link HPDP Biotin	Thermo Scientific™	Cat# A35390
Dynabeads MyOne Streptavidin C1	Thermo Scientific™	Cat# 65001
Phenol:Chloroform:Iso-amyl alcohol (125:24:1)	Sigma	Cat# P1944-100ML
Dynabeads™ Protein G	Thermo Scientific™	10003D
Recombinant protein: *S. cerevisiae* Rpb4–Rpb7-3C-His_6_ Q96A, H97A, F109K, (Rpb7)	This study	N/A
BirA biotin ligase	Merck	SRP0717

**Critical commercial assays**		

NEBNext Ultra II DNA Library Prep Kit for Illumina	New England Biolabs	E7645S
NEBNext Ultra II Directional RNA Library Prep Kit for Illumina	New England Biolabs	Cat# E7760S
Ribominus Transcriptome Isolation Kit	Invitrogen	Cat# K1550-03
Superscript IV Reverse Transcriptase	Invitrogen	Cat# 18090010
High Sensitivity DNA Assay	Agilent	Cat# 5067-4626
Power SYBR Green PCR	Thermo Scientific™	Cat# 4367659

**Deposited data**		

Original uncropped gels and blots, dynamic light scattering (DLS) analysis, quantification of RNA Pol II dimerization	This study	Mendeley data: https://dx.doi.org/10.17632/hyp4ym8hhm.1
RNA Pol II homodimer (consensus cryoEM map)	This study	EMDB: EMD-15358
RNA Pol II homodimer (focused cryoEM map for monomer 1)	This study	EMDB: EMD-15359
RNA Pol II homodimer (focused cryoEM map for monomer 2)	This study	EMDB: EMD-15360
RNA Pol II – APT complex (cryoEM map)	This study	N/A
Glc7-Ref2_348-406_ crystal structure	This study	PDB: 8A8F
Glc7 crystal structure (for molecular replacement)	PDB: 7QWJ	PDB: 7QWJ
RNA Pol II crystal structure (for rigid fit of the cryoEM maps)	Barnes et al.^42^	PDB: 5C4X
Yeast transcription pre-initiation complex (PIC)	Schilbach et al.^66^	PDB: 5OQM
Yeast transcription elongation complex (EC)	Ehara et al.^39^	PDB: 5XON
ChIP-seq of RNA Polymerase II (Rpb3) from wild-type and Rpb7-QHF cells	This study	ArrayExpress: E-MTAB-13369
4tU RNA-seq from from wild-type and Rpb7-QHF cells	This study	ArrayExpress: E-MTAB-13370

**Experimental models: Cell lines**		

Sf9	Oxford Expression Technologies Ltd.	Cat# 600100-Sf9 cells

**Experimental models: Organisms/strains**		

wt (protease deficient) *S. cerevisiae*: MATalpha pra1-1 prb1-1 prc1-1 cps1-3 ura3delta5 leu2-3 his-	Heinemeyer et al.^67^	JWY104 (S1-19)
RPB3-His6-Bio wt *S. cerevisiae*: MATa ura3-52 trp1 leu2-delta1 his3-delta200 pep4::HIS3 prb1-delta1.6R can1 GAL RPB3-His6-BIO::URA3	Kireeva et al.^68^	BJ5464 (S3-68)
rpb4Δ *S. cerevisiae*: MATa, ade2-1, his 3-11,15, leu2-3,112, trp1-1, ura3-1, rpb4::HIS3	Allepuz-Fuster et al.^35^	OCSC2065 (S4-16)
RPB3-TEVHis_6_-SII rpb4Δ *S. cerevisiae*: MATa, ade2-1, his 3-11,15, leu2-3,112, trp1-1, ura3-1, rpb4::HIS3 RPB3-TEV-His6-SII	This study	MCY101 (S4-17)
RPB3-TEVHis6-SII, Rpb1-3C-CTD wt *S. cerevisiae*: MATa, ade2-1, his 3-11,15, leu2-3,112, trp1-1, ura3-1,rpb4::HIS3 RPB3-TEV-His6-SII	This study	MCY102 (S4-39)
*S. cerevisiae*: MATalpha pra1-1 prb1-1 prc1-1 cps1-3 ura3delta5 leu2-3 his-RPB1-3C-CTD RPB3-His6-SII rpb7(Q96A, H97A, F109K)	This study	MCY106 (S4-46)
*S. cerevisiae*: MATα {leu2-3,112 trp1-1 can1-100 ura3-1::pMK200 ade2-1his3-11,15} REF2 (P531G)-1xmAID-3xFLAG	This study	JRY219 (S4-50)
*S. cerevisiae*: JRY219 + pRS314 (REF2)	This study	JRY228 (S4-59)
*S. cerevisiae*: JRY219 + pRS314 (Ref2-I372D,F374K, Y384E)	This study	JRY229 (S4-60)
*S. cerevisiae*: MATα {leu2-3,112 trp1-1 can1-100 ura3-1::pMK200 ade2-1his3-11,15}	This study	JRY216 (S4-47)

**Oligonucleotides**		

List of DNA and RNA oligonucleotides used across the study	This study and Ehara et al.^39^	Table S1

**Recombinant DNA**		

pACEBac1-Ref2–3C-SII	Kumar et al.^65^	P21-53
pACEBac1-Glc7	Kumar et al.^65^	P21-59
pACEBac1-Swd2	Kumar et al.^65^	P21-57
pACEBac1-Pta1	Kumar et al.^65^	P21-54
pACEBac1-Pti1	Kumar et al.^65^	P21-58
pACEBac1-Ssu72	Kumar et al.^65^	P21-56
pACEBac1-Syc1	This study	P21-55
pBIG2ab -CPF phosphatase module (Ssu72-Pti1-Glc7-Ref2-SII-Swd2-Pta1)	Kumar et al.^65^	P27-37
pBIG2ab -APT (Ssu72-Pti1-Glc7-Ref2-SII-Swd2-Pta1-Syc1)	This study	P24-11
pBig1a-CPF-polymerase module (Cft1-Pap1-Pfs2-SII-Fip1-Yth1)	Hill et al.^36^	P20-3
pBig1a-CPF-nuclease module (Mpe1, Ysh1, Cft2)	Hill et al.^36^	P20-6
pBig1a-Ref2-TEV-SII–Glc7–Swd2	This study	P24-13
pBig1a-Ref2ΔIDR-TEV-SII–Glc7–Swd2	This study	P30-64
pBig1b-Pta1-TEV-SII–Pti1–Ssu72–Syc1	This study	P25-23
pML104 (CRISPR-Cas9 in yeast)	Addgene	Cat# 67638
pML107 (CRISPR-Cas9 in yeast)	Addgene	Cat# 67639
pETDuet-1-Rpb7-3C-HIS-Rpb4	This study (Epoch Life Science)	P28-37
pETDuet-1-Rpb7-3C-HIS-Rpb4 (mutant: dimerization deficient)	This study	P30-60
pET24a -His_10_-SUMO-Glc7–Ref_2348-406_–GSGSG–SPSYSPTSPSYSPT (crystal structure)	This study (Epoch Life Science)	P28-39
FX-Ref2 348-523 - His-SUMO(N)	This study	P25-56
FX-Glc7 - His-SUMO(N)	This study	N/A
pNHS-FX-GST-Ref2_348-434_	This study	P25-45
pNHS-FX-GST-Ref2_348-406_	This study	P25-44
CF IA (_6H-_Rna14-Rna15- _6H-_Pcf11-Clp1)	Kumar et al.^65^	P20-24
CF IB (_6H-_Hrp1)	Hill et al.^36^	P2-43
pST1933	Tanaka et al.^47^	P19-52
pRS314 (REF2)	This study	P42-38
pRS314 (Ref2-I372D,F374K, Y384E)	This study	P42-39

**Software and algorithms**		

Prism 9 (v. 9.4.0)	N/A	https://www.graphpad.com
cryoEF	Naydenova and Russo^69^	https://www.mrc-lmb.cam.ac.uk/crusso/cryoEF/
ProtParam	Gasteiger et al.^70^	https://web.expasy.org/protparam/
Relion 3.1	Zivanov et al.^71^	https://github.com/3dem/relion
XIA2	Winter et al.^72^	N/A
XDS	Kabsch^73^	N/A
XSCALE	Kabsch^74^	N/A
Phaser	McCoy^75^	N/A
phenix.refine	Adams et al.^76^	N/A
Refmac	Murshudov et al.^77^	N/A
MolProbity	Williams et al.^78^	N/A
PDBePISA tool	Krissinel and Henrick^79^	https://www.ebi.ac.uk/pdbe/pisa/
Coot (v. 0.9.5.1-pre)	Emsley et al.^80^	https://www2.mrc-lmb.cam.ac.uk/personal/pemsley/coot
UCSF ChimeraX (v. 1.4)	Pettersen et al.^81^	https://www.cgl.ucsf.edu/chimerax/
PyMOL (v. 2.3.5)	Schrödinger, LLC	https://pymol.org/2/
Clustal Omega	Goujon et al.^82^; Sievers et al.^83^	http://www.ebi.ac.uk/Tools/msa/clustal/ (RRID:SCR_001591)
AlphaFold2	Varadi et al.^52^ and Jumper et al.^53^	https://colab.research.google.com/github/sokrypton/ColabFold/blob/main/AlphaFold2.ipynb
DYNAMICS®	Wyatt Technology	https://www.wyatt.com/products/software/dynamics.html
ASTRA	Wyatt Technology	https://www.wyatt.com/products/software/astra.html
FIJI (ImageJ)	Schindelin et al.^84^	https://fiji.sc/
Integrative Genome Viewer (v. 2.4.11)	Robinson et al.^85^	https://software.broadinstitute.org/software/igv/
Bowtie (v2.5.1)	Langmead et al.^86^	https://bowtie-bio.sourceforge.net/index.shtml
STAR (v. 2.6.0a)	Dobin et al.^87^	https://github.com/alexdobin/STAR
SAMtools (v1.6)	Li et al.^88^	http://samtools.sourceforge.net/
Deeptools (v3.5.1)	Ramirez et al.^89^	https://deeptools.readthedocs.io/en/develop/
Seq-Plots	Stempor and Ahringer^90^	https://github.com/Przemol/seqplots
MACS2 (v2.2.6)	Zhang et al.^91^	https://hbctraining.github.io/Intro-to-ChIPseq/lessons/05_peak_calling_macs.html
DiffBind (v3.10.0)	Ross-Ines et al.^92^	https://bioconductor.org/packages/release/bioc/html/DiffBind.html
R (v4.3.0)	N/A	https://www.r-project.org
Remote 3DFSC Processing Server	Tan et al.^93^	https://3dfsc.salk.edu/
PDBeFold	Krissinel and Henrick^94^	https://www.ebi.ac.uk/msd-srv/ssm/

### Resource availability

#### Lead contact

Further information and requests for resources and reagents should be directed to and will be fulfilled by the lead contact, Lori Passmore (passmore@mrc-lmb.cam.ac.uk).

#### Materials availability

All unique/stable reagents generated in this study are available from the lead contact with a completed Materials Transfer Agreement.

#### Data and code availability

- The models and maps for the structures presented have been deposited in the PDB and EMDB. Sequencing data have been deposited in ArrayExpress. The accession numbers are listed in the key resources table. Original gel and western blot files, as well as DLS and SEC MALS data, have been deposited on Mendeley Data: https://dx.doi.org/10.17632/hyp4ym8hhm.1. These are all publicly available as of the date of publication. Micrographs reported in this paper will be shared by the lead contact upon request.
- This paper does not report original code.
- Any additional information required to reanalyse the data reported in this paper is available from the lead contact upon request.

### Experimental model and study participant details

All gene cloning, manipulation, and plasmid propagation steps were carried out in *Escherichia coli* TOP10 cells grown in 2 X TY media supplemented with appropriate selection antibiotics. *E.coli* DH10 EMBacY cells were used for bacmid isolation.

Recombinant Rpb4-Rpb7, Ref2 truncations and Glc7 were expressed in *E. coli* BL21 (DE3) cells or Rosetta 2 (DE3) pLysS cells grown in 2 X TY media until an OD_600nm_ of 0.6 – 1.0 was reached. Expression was induced with 0.5 mM IPTG for an appropriate time and temperature as described. For all other recombinant proteins and complexes, the *Spodoptera frugiperda* Sf9 cell line was used for baculovirus-driven overexpression. Suspension cultures were grown at 27°C, 140 rpm in Insect-XPRESS protein-free insect cell medium with L-glutamine (Lonza).

Functional studies were performed in *Saccharomyces cerevisiae* strains listed in the key resources table. Yeast strains were grown at 30°C with shaking at 180 rpm in YPD media (YPD media per L: 20 g peptone, 20 g D-glucose, 10 g yeast extract). Synthetic complete or drop out media was used as indicated. Media was supplemented with appropriate selection antibiotics.

### Method details

#### Cloning

##### Genome editing of S. cerevisiae RNA Pol II

Endogenous yeast RNA Pol II from wild-type protease-deficient (JWY104) and Δ*RPB4* (OCSC2065) strains (key resources table), was purified by insertion of a His_6_-TEV-StrepII-tag (SII) at the C terminus of Rpb3 via CRISPR-Cas9-mediated homologous recombination.^95^ The same approach was used to introduce a 3C protease cleavage site within the *RPB1* ORF before the start of the CTD (residue 1454 of Rpb1) and to create the Rpb7^QHF^ allele in JWY104. We also used CRISPR-Cas9 to insert the mini-auxin induced degron tag at the C terminus of Ref2 (Ref2-mAID) in W303-1b cells. CRISPR-Cas9 engineering was performed based on Laughery and Wyrick.^95^ First, we designed a 20 nt-long single guide RNA (sgRNA) matching the target DNA sequence harboring a protospacer-adjacent motif (PAM) within an 8-20 bp window from the site being edited. To design the optimal sgRNA for *S. pyogenes* Cas9 with a 5ʹ-NGG-3ʹ PAM site, we utilized the online resource CRISPOR.^96^ Synthetic oligonucleotide sequences encoding the selected sgRNAs (Table S1) were cloned via *BclI* and *SwaI* restriction sites into a pML104 vector,^95^ which expresses the Cas9 enzyme from *S. pyogenes* and carries a *URA3* marker. Double stranded DNA repair templates (donor DNA; Table S1) were designed with ∼30-40 bp homology arms and carried point mutations, preferably within the PAM, so that the target sequence will not be re-cleaved after the editing. The donor DNA to generate the Ref2-mAID strain was amplified from pST1933,^47^ excluding the KanMX casette.

25 ng vector expressing sgRNA and 2 μg of the relevant donor DNA were co-transformed into yeast,^97^ and cells selected on SD/MSG agar -URA dropout plates. Colonies were screened for the intended modifications by PCR and sequencing. Positive clones were counter-selected on 5-fluoroorotic acid (5-FOA) containing plates to ensure removal the pML104 vector. All sgRNA and donor sequences are available in Table S1. To complete construction of the Ref2-mAID strain, we transformed StuI-digested pMK200 for recombination into the endogenous URA3 locus of W303-1b cells.^47^

##### Generation of vectors carrying APT complexes

For APT, *E. coli* codon*-*optimized genes encoding Pta1 (Uniprot Q01329), Ref2-StrepII-tag (SII) (Uniprot P42073), Pti1 (Uniprot O49339), Swd2 (Uniprot P36104), Glc7 (Uniprot P32598), Ssu72 (Uniprot P53538), and Syc1 (Q08553) (Gene Art, Thermo Fisher) were amplified and cloned into pACEBac1 through *BamHI* and *XbaI* restriction sites. Ref2 contained two sequence variations not present in the consensus sequence (A291V, P389S). APT genes were subsequently amplified from pACEBac1 and assembled by Gibson assembly into vectors from a modified biGBac system.^36^^,^^98^ Syc1 and Pta1 were inserted into pBig1a; Ref2-SII, Pti1, Swd2, Glc7 and Ssu72 were inserted into pBig1b. Correct integration was verified by SwaI digestion. A pBig2ab vector containing all seven APT subunits was then assembled via a second Gibson reaction. The same approach was followed to clone the Ref2-SII, Glc7, Swd2 into pBig1a, and Pta1-SII, Pti1, Ssu72, Syc1 into pBig1b. For Ref2ΔIDR-SII–Glc7–Swd2, the IDR region of Ref2 (residues 230–338) was deleted.

##### Glc7 and Ref2 C-terminal truncations

Genes encoding Glc7 and Ref2_348-523_ were amplified from *E. coli* codon*-*optimized genes (Gene Art, Thermo Fisher) and cloned into FX cloning vectors carrying an N-terminal His_10_-SUMO tag (gift from Dr. Harvey MacMahon, MRC-LMB). Genes encoding Ref2_348-434_ and Ref2_348-406_ were amplified and cloned into the *BamHI* and *XhoI* sites of a pGEX-6P-2 vector, downstream of a GST-tag followed by a 3C cleavage site.

##### Glc7–Ref2_348-406_ fusion protein

The coding sequence for His_10_-SUMO–Glc7*–*Ref2_348-406_ followed by a Gly-Ser-Gly-Ser-Gly linker and two RNA Pol II CTD repeats (SPSYSPT) was synthesized and cloned into a pET24b expression vector by Epoch Life Science.

##### Recombinant Rpb4–Rpb7 (RNA Pol II stalk)

*RPB4* (Uniprot P20433) and *RPB7* (Uniprot P34087) genes were synthesized and cloned (Epoch Life Science) into a bicistronic pETDuet-1 vector. The gene encoding Rpb7-3C-His_6_ was inserted in the first cassette, and Rpb4 was inserted into the second. For the generation of the mutant RNA Pol II stalk, point mutations were introduced by site-directed mutagenesis in *RPB4* (Arg209Ala) and in *RPB7* (Glu96Ala, His97Ala, Phe190Lys, His158Ala).

##### Native Ref2 rescue constructs

A *REF2* cassette containing sequences 200 bp upstream and 173 bp downstream (to include its native promoter and terminator, respectively) was amplified from the yeast genome and cloned into *BamHI*-digested pRS314 to generate the Ref2^WT^ construct. This construct was used as template to generate the Ref2^mut^ construct carrying point mutations in Ref2 (Ile372Asp, Phe374Lys, Tyr384Glu) by site-directed mutagenesis. Primers used for cloning and mutagenesis are listed in Table S1. Ref2^WT^ and Ref2^mut^ were transformed into Ref2-mAID and colonies selected on SD/MSG agar -TRP -URA dropout plates.

##### Growth curves

Growth curves were performed using a Tecan M200 Pro 96-well instrument with shaking at 30^o^C. Independent biological replicates were grown overnight in the appropriate selection media and diluted (1:80) in 200 μl YEPD. For treatment with IAA, cells were treated throughout the growth curve with 1 mM IAA (from 100 mM IAA freshly made stock) or the equivalent volume of solvent (DMSO).

#### Recombinant proteins from insect cells

Preparation of bacmids in EmBacY cells, transfection into Sf9 cells, initial and secondary baculovirus generation, and protein overexpression in Sf9 cells were carried out as previously described.^36^^,^^65^ For large-scale protein expression, 5-10 ml secondary virus was used to infect 500 ml suspension Sf9 cells (at 2x10^6^ cells/ml and >90% viability) grown in 2-litre roller bottle flasks. All Sf9 cells were grown in insect-EXPRESS (Lonza) media, incubated at 27°C and at 140 rpm. No additional supplements were provided to the media.

APT, the APT subcomplex Pta1-SII–Pti1–Ssu72–Syc1, CPF phosphatase module,^65^ CPF-core (polymerase and nuclease modules),^36^ the nuclease-phosphatase CPF module^65^ and CPF polymerase module^14^ were purified from Sf9 cell pellets according to the following protocol. Pellets from 4-litre cultures were harvested at 4,000 rpm in a JLA 8.1000 rotor and resuspended up to 120 ml with lysis buffer (50 mM Na-HEPES pH 8.0, 300 mM NaCl, 0.5 mM Mg(OAc)_2_, 0.5 mM TCEP and 10 % v/v glycerol) enriched with 4x EDTA-free protease inhibitor cocktail tablets (Roche, cat. No. 11836153001). Cells were lysed by sonication using a 10 mm tip on a VC 750 ultrasonic processor (Sonics). Sonication was performed at 70% amplitude with 5 seconds ‘on’ time and 10 seconds ‘off’ time and was followed by ultra-centrifugation at 45,000 rpm for 35 minutes in a Ti45 Beckman rotor. Clear lysates were treated with 4 ml BioLock (IBA, cat. No. 2-0205-050) before incubation with 2 ml bed volume of StrepTactin resin (IBA, cat. No. 2-1201-025) at 4 °C for 1 hour. Beads were washed with 100 ml lysis buffer, and proteins carrying a Strep-II tag were eluted by incubation with 20-25 ml lysis buffer supplemented with 6 mM desthiobiotin (IBA, cat. No. 2-1000-005). The filtered eluate (0.45 μM filter) was diluted to about 150 mM NaCl with buffer A (10 mM Na-HEPES pH 8.0, 0.5 mM TCEP and 5 % v/v glycerol) and loaded on a 6 ml Resource Q anion exchange chromatography column (Cytiva, cat. No. 17117901). Protein complexes were eluted by applying a gradient of 15–60 % buffer B (buffer A supplemented with 1 M NaCl) over 7-8 CV. The relevant fractions were pooled, and proteins concentrated in Amicon Ultra-15 concentrators (Merck) before flash freezing and storage at -80 °C.

For the APT subcomplex Ref2-SII–Glc7–Swd2 and Ref2ΔIDR-SII–Glc7–Swd2, the ion exchange chromatography step was replaced by gel filtration on a Superdex 200 Increase 10/300 GL (Cytiva, cat No. 28990944) equilibrated in 10 mM K-HEPES pH 8.0, 150 mM KCl, 0.5 mM TCEP and 5 % glycerol.

Recombinant CPF was reconstituted by incubating equimolar amounts of purified polymerase module and nuclease-phosphatase module on ice for 30 minutes, then injecting them on a Superose 6 Increase 10/300 GL column (Cytiva, cat No. 29091596) in 10 mM K-HEPES pH 8.0, 150 mM KCl, 0.5 mM TCEP and 5 % glycerol.^65^ Peak fractions were analyzed by SDS-PAGE for proper stoichiometry, pooled, concentrated to 8.6 mg/ml, flash frozen and stored at -80 °C.

#### Purification of endogenous yeast RNA Pol II

The purification of RNA Pol II from yeast was based on a previously described protocol^99^ with several modifications. For wild type (strain MCY102) and the RNA Pol II Δ-stalk (strain MCY101), 50-litre yeast cultures were grown in flasks in YEPD medium at 30°C, harvested at an OD_600_ of 8.0 (∼18-20 hours), resuspended in lysis buffer (200 mM Tris-HCl pH 8.0, 150 mM KCl, 10 μM Zn(OAc)_2_, 10 mM DTT, 1 % DMSO, and 10% w/v glycerol) supplemented with 5x protease EDTA-free inhibitor tablets (Roche, cat. No. 11836153001), and frozen by dropping the suspended yeast into liquid nitrogen. Lysis was performed through cryogenic grinding of the frozen cells by a Freezer Mill 6870 (SPEX CertiPrep). After thawing the ground material, the crude lysate was subjected to centrifugation for 30 minutes at 13,750 x g in a JLA 16.250 Beckman rotor. The supernatant was then ultracentrifuged at 45,000 rpm in a Ti45 Beckman rotor for 1.5 hours. (NH_4_)_2_SO_4_ was added to the cleared lysate until 50 % w/v saturation and precipitated overnight at 4 °C with gentle stirring. The precipitated solution was centrifuged at 33,768 x g in a JLA 16.250 Beckman rotor, and the supernatant was discarded. The (NH_4_)_2_SO_4_ pellet was resolubilized with a buffer without salt (50 mM Tris-HCl pH 8.0, 0.5 mM EDTA, 10 μM Zn(OAc)_2_, 7 mM imidazole, 2 mM DTT, and 10% w/v glycerol) supplemented with 1x protease inhibitor tablet, by adding 14 ml of buffer per 10 g pellet. The resuspended material was then subjected to centrifugation at 15,000 rpm in a JA 25.50 Beckman rotor for 10 minutes, passed through a 0.45 μM filter, and loaded on a 5 ml HisTrap HP column (Cytiva, cat. No. 17524802) for affinity purification. After washes, bound proteins were eluted with 200 mM imidazole over 30 CV, and the peak fractions were pooled for subsequent incubation with 1 ml (bed volume) pre-equilibrated StrepTactin resin (IBA, cat. No. 2-1201-025) for 1 hour and washed with buffer A containing 20 mM Tris-HCl pH 8.0, 0.5 mM EDTA, 150 mM KCl, 2 mM DTT, and 10 % glycerol. RNA Pol II (Rpb3-SII) was eluted with 20 ml of buffer A supplemented with 8 mM desthiobiotin (IBA, cat. No. 2-1000-005) and filtered through a 0.45 μM filter. It was then applied to a 1 ml MonoQ 5/50 GL column (Cytiva, cat. No. 17516601) and eluted with a linear gradient of 20–60 % buffer B (20 mM Tris-Acetate pH 8.0, 0.5 mM EDTA, 2 M KAc, 10 mM DTT, and 10 μM Zn(OAc)_2_, and 10 % glycerol) over 60 CV. Peak fractions were pooled and concentrated using a 30 kDa cut-off Amicon Ultra-15 concentrator (Merck, cat No. UFC903024) to a typical concentration of about 6 mg/ml, before flash freezing and storage at -80 °C.

For the RNA Pol II Δ-stalk purification, the 50-litre culture was allowed to grow for ∼40 hours at 28°C and harvested at an OD_600_ of 5.0. Five additional protease EDTA-free inhibitor tablets (Roche, cat. No. 11836153001) and 5 μM Pepstatin A were added to the resuspension buffer to prevent CTD degradation.

For the yeast strain carrying a C-terminal His_6_-Bio tag on Rpb3 (BJ5464) the purification protocol was the same, except there was no incubation with StrepTactin resin.^99^ After anion exchange, this version of RNA Pol II required further polishing by gel filtration on a Superose 6 Increase 10/300 equilibrated in 10 mM K-HEPES pH 8.0, 100 mM KCl, 0.5 mM TCEP and 5 % glycerol.

#### Recombinant proteins from *Escherichia coli*

##### Glc7 and Glc7 fusion proteins

His_10_–SUMO–Glc7 and the Glc7*–*Ref2_348-406_ chimeric protein used for crystallography were purified from 36 litres of transformed Rosetta pLysS competent cells grown in 2xTY, induced with 0.25 mM IPTG at 18 °C for 18-20 hours, harvested, and resuspended in lysis buffer (50 mM Tris-HCl pH 8.0, 750 mM NaCl, 20 mM imidazole, 2 mM MnCl_2_, 1 mM TCEP 10 % v/v glycerol) supplemented with 4x EDTA-free protease inhibitor cocktail tablets (Roche, cat. No. 11836153001) and 4 μg/ml DNase I. After lysis by sonication and centrifugation at 45,000 rpm in a Beckman 45Ti rotor for 30 minutes, the clear lysate was applied to a 5 ml HisTrap HP column. Bound proteins were eluted with 300 mM imidazole after extensive washes with 40 mM imidazole. Fractions containing the His_10_–SUMO–Glc7*–*Ref2_348-406_–GSGSG–SPSYSPTSPSYSPT chimera were pooled and dialyzed overnight against 50 mM Tris-HCl pH 8.0, 500 mM NaCl, 2 mM MnCl_2_, 0.5 mM TCEP, 5% v/v glycerol in the presence of SUMO protease. The sample was concentrated down to 5 ml volume (Vivaspin, Sartorius) and further purified through a HiLoad 26/600 Superdex 200 pg (Cytiva) size-exclusion column equilibrated in the dialysis buffer. Peak fractions were analyzed by SDS-PAGE, pooled, concentrated to 13 mg/ml, and used for crystallization trials.

##### Recombinant Rpb4–Rpb7

Competent BL21(DE3) *E. coli* cells were transformed with 50 ng of the pETDuet vector carrying Rpb4 and Rpb7-3C-His_6_. For both wild type and mutant Rpb4–Rpb7, 6-litre cultures were grown in 2xTY medium to an OD of 0.8 and induced overnight at 18 °C with 0.25 mM IPTG. Pellets were resuspended up to 180 ml with lysis buffer (50 mM Tris-HCl pH 8.0, 300 mM NaCl, 20 mM imidazole, 0.5 mM TCEP, 10% glycerol) supplemented with 4x EDTA-free protease inhibitor cocktail tablets (Roche, cat. No. 11836153001) and 4 μg/ml DNase I, sonicated, and subjected to centrifugation at 45,000 rpm in a Ti45 Beckman rotor for 30 minutes. Cleared lysate was passed through a 0.45 μM filter, and loaded on a 5 ml HisTrap HP column, washed, and eluted with a buffer containing 20 mM Tris-HCl pH 8.0, 300 mM NaCl, 200 mM imidazole, 0.5 mM TCEP and 5% glycerol. Peak fractions were pooled, concentrated to 3 ml volume (Vivaspin, Sartorius), and further purified on a HiLoad 16/600 Superdex 200 pg (Cytiva) size-exclusion column equilibrated in 10 mM K-HEPES pH 8.0, 150 mM KCl, 0.5 mM TCEP, and 5% glycerol. Peak fractions were pooled and concentrated to 36 mg/ml prior to snap freezing and storage at -80 °C. To remove high-molecular weight contaminants, mutant Rpb4–Rpb7 was further polished on a 1 ml HiTRAP heparin HP column (Cytiva, cat. No. 1704703) through a 7.5 to 50 % elution over 25 CV with a buffer consisting of 10 mM K-HEPES pH 8.0, 1M NaCl, 0.5 mM TCEP and 5 % glycerol. The protein complex was concentrated to 6.8 mg/ml and stored at -80 °C.

##### Ref2 C-terminal truncations

Competent BL21(DE3) *E. coli* cells were transformed with 50 ng of the FX vector containing the sequence encoding His_10_-SUMO-Ref2_348-523_. 5-litre cultures were grown in 2xTY medium until an OD of 0.8 and induced overnight at 20 °C with 0.25 mM IPTG. Pellets were resuspended in 120 ml lysis buffer (50 mM Tris-HCl pH 8.0, 300 mM NaCl, 5 mM imidazole, 1 mM TCEP and 10 % glycerol) supplemented with 2x EDTA-free protease inhibitor cocktail tablets (Roche, cat. No. 11836153001) and 4 μg/ml DNase I, sonicated, and subjected to centrifugation at 45,000 rpm in a Ti45 Beckman rotor for 30 minutes. Cleared lysate was applied to 4 ml bed volume Ni-NTA Agarose beads (Qiagen, cat. No. 30210) for 1 hour. After washing beads with 20 mM Tris-HCl pH 8.0, 1 M NaCl, 20 mM imidazole, 1 mM TCEP and 5 % glycerol, His_10_-SUMO-Ref2_348-523_ was eluted by incubation with 40 ml buffer containing 20 mM Tris-HCl pH 8.0, 300 mM NaCl, 250 mM imidazole, 1 mM TCEP. After overnight cleavage with SUMO protease to remove the tag, the protein was diluted with buffer A (25 mM MES pH 6.5, 1 mM TCEP and 5 % glycerol) to about 50 mM NaCl for cation exchange chromatography. Ref2_348-523_ was then applied to a 1 ml Resource S column (Cytiva, cat. No. 17-1177-01), washed and eluted with a gradient of 5-40 % buffer B (25 mM MES pH 6.5, 1 M NaCl, 1 mM TCEP and 5 % glycerol) over 80 CV. Eluted fractions were pooled, concentrated to 2 ml volume in Amicon Ultra-15 concentrators (Merck), and injected on a HiLoad 16/600 Superdex 75 pg (Cytiva) size-exclusion column equilibrated in 25 mM MES pH 6.5, 200 mM NaCl, 1 mM TCEP and 5% glycerol. Purified protein fractions were pooled, concentrated, and de-salted for further purification on a 1 ml Heparin column (Cytiva). High-purity Ref2_348-523_ was eluted through a linear 4-50 % gradient of a buffer containing 1M NaCl, over 50 CV. Peak fractions were pooled, concentrated to 7 mg/ml and stored at -80 °C after snap freezing.

For Ref2_348-434_ and Ref2_348-406_, 50 ng of the cloned pGEX-6P-2 were used to transform competent BL21(DE3) *E. coli* cells. 6-litre cultures were grown in 2xTY medium until an OD of 0.8 and induced overnight at 20 °C with 0.25 mM IPTG. Pellets were resuspended in 180 ml lysis buffer (50 mM Tris pH 8.0, 300 mM NaCl, 0.5 mM EDTA, 1 mM TCEP and 10 % glycerol), sonicated, and subjected to centrifugation at 45,000 rpm in a Ti45 Beckman rotor for 30 minutes. Cleared lysates were incubated with 5 ml bed volume Glutathione Sepharose 4B resin (GE Healthcare, cat. No. 17-0756-05) for 1 hour. Beads were washed with lysis buffer containing 1 M NaCl, de-salted in an 80 mM NaCl-containing buffer, and incubated overnight with 3C protease for GST-tag removal. Ref2_348-434_ or Ref2_348-406_ proteins were then loaded on a 1ml Resource S column, washed, and eluted with a gradient ranging from 8 to 30% buffer B (25 mM MES pH 6.5, 1 M NaCl, 1 mM TCEP and 5 % glycerol) over 30 CV. Proteins were concentrated for injection on a HiLoad 16/600 Superdex 75 pg in 25 mM MES pH 6.5, 200 mM NaCl, 1 mM TCEP and 5% glycerol. Peak fractions were frozen and stored after concentration.

#### *In vitro* Strep-Tactin pull-down assays

Reactions were set up in a total volume of 40 μl. 1.5 μM SII-tagged apo-RNA Pol II was immobilized on 10 μl (bed volume) of StrepTactin resin equilibrated in wash buffer (10 mM K-HEPES pH 8.0, 100 mM KCl, 0.5 mM TCEP, and 0.1 % v/v Tween-20). After rinsing to remove excess unbound bait, RNA Pol II-bound beads were incubated with 3 μM of the indicated prey proteins for 1 hour on ice without shaking. Beads were then washed three times with 150 μl wash buffer (600 x g, 30 seconds, 4 °C), and bound species eluted by SDS-loading dye supplemented with 5% β-mercaptoethanol. Input for both bait and prey proteins were included in the gel as reference. The assays were resolved by SDS-PAGE (NuPAGE™ 4-12%, Bis-Tris, Invitrogen) run in MES-SDS buffer at 190 V for 50 minutes. For the pull-down experiment in Figure S1B, 1 μM SII-tagged CPF subcomplexes and APT were immobilized on beads and incubated with 2 μM apo-RNA Pol II in solution.

#### Size exclusion chromatography assays

All analytical size exclusion chromatography (SEC) binding assays were conducted on an ÄKTAmicro system (GE Healthcare) using a Superose 6 Increase 3.2/300 column (Cytiva, cat No. 29091598) equilibrated in 10 mM K-HEPES pH 8.0, 100 mM KCl, 0.5 mM TCEP, and 5% v/v glycerol. 2.5 μΜ apo-RNA Pol II was incubated on ice for 10 minutes with a 1.8 molar excess of APT or APT-subcomplexes in a total volume of 50 μl and spun (5 minutes at 21,130 x g) before injection. The eluted volume was monitored by A_260-280_, collected in 50 μl fractions and analyzed by SDS-PAGE (NuPAGE™ 4-12%, Bis-Tris, Invitrogen) run in MES-SDS buffer at 190 V for 50 minutes.

Interaction studies between Glc7 and Ref2 were carried out by incubating 5 μΜ Glc7 with 10 μΜ Ref2 C-terminal truncations in 50 μl. Co-migration was assessed after injection on a Superdex 200 Increase 3.2/300 column (Cytiva, cat No. 28990946). The eluted volume was collected in 50 μl fractions and analyzed on SDS-PAGE (Bolt™ 4-12%, Bis-Tris, Invitrogen) run in MES-SDS buffer at 200 V for 35 minutes.

#### *In vitro* dephosphorylation assays

200 μg RNA Pol II was hyperphosphorylated by incubation with 280 units of human MAP Kinase 2/Erk2 Protein (Merck, cat. No, 14-550) in a total volume of 50 μl in kinase buffer (50 mM Tris-HCl pH 7.4, 200 mM KCl, 5 mM MgCl_2_, 0.5 mM EGTA, 2 mM DTT) in the presence of 200 μM ATP at 30 °C for 60 minutes. Excess ATP and MAP kinase were removed by gel filtration on a Superose 6 Increase 3.2/300 column equilibrated in 10 mM K-HEPES pH 8.0, 100 mM KCl, 0.5 mM TCEP, and 5% v/v glycerol.

500 nM hyperphosphorylated RNA Pol II was incubated with 1 μM CPF, APT, CPF/APT subcomplexes, Glc7 or Glc7–Ref2_348-406_ in 20 μl reactions in phosphatase buffer (20 mM K-HEPES pH 8.0, 150 mM KCl, 0.5 mM Mg(OAc)_2_, 0.5 mM MnCl_2_, 2 mM DTT). Reactions were incubated at 30 °C for 90 minutes and quenched by the addition of SDS-loading dye supplemented with 5 % β-mercaptoethanol. After heating at 70 °C, samples were analyzed by SDS-PAGE (Bolt™ 4-12%, Bis-Tris, Invitrogen), run in MES-SDS buffer at 200 V for 30 minutes, followed by immunoblot against phosphorylated Tyr1 or Ser5 CTD residues [antibodies 3D12 (1:7 in milk) and 3E8 (1:2000 in milk) respectively (Antibody Core Facility - Helmholtz-Zentrum Munich)]. Anti-Rpb3 [1Y26 antibody (abcam ab202893) 1:1000 in milk] was used as a loading control. Membranes in Figures S1A and S8A (left) were incubated with fluorescent secondary antibodies (Goat anti-mouse IgG DyLight 800 ThermoFisher #SA5-10176 and Goat anti-rat IgG DyLight 800 ThermoFisher #SA5-10024), and images acquired on a LI-COR Odyssey instrument. Membranes in Figures S8A (right) and S10D were incubated with HRP-conjugated secondary antibodies, developed by Amersham ECL Prime Western Blotting Detection Reagent (Cytiva, catalog # RPN2236), and images acquired on a ChemiDoc (Bio-Rad Laboratories).

#### Ref2-mAID depletion immunoblots

Whole cell extracts were prepared with an alkaline lysis protocol as previously described.^100^ Briefly, three independent 1 ml overnight yeast cultures (biological replicates) were subcultured in 25 ml YEPD until mid-log phase (∼6 hours). 5 ml of culture were harvested before the addition of auxin, and every 15 min after addition of 1 mM IAA. Cells were resuspended in 0.5 ml 100 mM NaOH, and incubated at room temperature for 5 minutes. Cells were then harvested and resuspended in 75 μl LDS sample buffer (Pierce, 84788) supplemented with 1 mM DTT. Sample was heated to 70^o^C and the extract separated from cell debris by centrifugation. 5-10 μl of cleared whole cell extract was run on a 4-12% Bis-Tris SDS-PAGE (Thermo, NP0323BOX) MOPS-SDS buffer at 200 V for 50 minutes. Proteins were transferred onto nitrocellulose membrane (Whatman Protran BA85, cat. No. 10401196) using the Trans Blot Turbo transfer apparatus (25 V, 1 A, 30 minutes) (Bio-Rad), and blocked with 5% milk. Blots were cut between the 75 kDa and 50 kDa marker bands. The top blot was probed with anti-FLAG M2 antibody (1:5,000 Sigma F1804), and the bottom blot was probed with anti-alpha tubulin [EPR13799] (1:5,000 Abcam ab184970). Secondary antibodies were used at 1:5,000 dilutions, and membranes visualized with ECL as described above.

#### Yeast analyses

##### Chromatin immunoprecipitation

Three independent 5 ml overnight cultures (biological replicates) were subcultured in 50 ml of YEPD and grown until mid-log phase. Cells were crosslinked with 1% formaldehyde at room temperature for 5 minutes and quenched with 125 mM glycine for 5 minutes. Cells were lysed (50 mM HEPES-KOH, 140 mM NaCl, 1 mM EDTA, 1% Triton X-100, 0.1% Na-Deoxycholate) by bead beating and chromatin sheared using a Bioruptor (48 cycles, 10 seconds on, 10 seconds off on a high setting). Cleared lysate was incubated with anti-Rpb3 1Y26 (abcam ab202893) (3 μl in 500 μl). 10% of cleared lysate was saved as input. 50 μl of lysis buffer-equilibrated protein G Dynabeads were used for immunoprecipitation. Beads were washed with wash buffer (10 mM Tris-HCl, 250 mM LiCl, 0.5% NP40, 1% Na-Deoxycholate, 1mM EDTA), high salt wash buffer (wash buffer supplemented with 500 mM NaCl) and 1x TE (10 mM Tris-HCl, 1 mM EDTA). Elution was done in elution buffer (50 mM Tris-HCl, 10 mM EDTA, 0.5% SDS, 5 mM CaCl_2_) at 65^o^C. ChIP eluate and inputs (with 50 μl elution buffer) were incubated with proteinase K at 65^o^C overnight. DNA was purified using the QIAquick PCR purification kit (Qiagen).

##### qPCR of ChIP and input DNA

qPCR reactions were performed using an Agilent ViiA 7 384-well instrument. Reactions from independent biological replicates were carried out in technical triplicates. The average Ct value from technical triplicates was used for downstream analyses. To calculate the average percentage IP/input, we first adjusted the input Ct values to reflect that they only represent 10% of the starting ChIP material. To do this, input Ct values were uniformly adjusted by adding log_2_ (0.1) = -3.32193 cycles to their Ct value. ChIP Ct values were normalized to their respective input Ct values (ΔCt = Ct^ChIP^-Ct^input^) and final percentage calculated (100 x 2^-ΔCt^). For each primer pair the average percentage was calculated and plotted using Prism 9 (9.4.1).

##### ChIP-seq library preparation

ChIP-seq libraries were prepared using the NEBNext Ultra II DNA Library Prep Kit for Illumina following the kit protocol. Importantly, 20 μl of ChIP and 1 μl of input DNA were used for library construction. Also, the adaptor was used with 1:10 dilution. For library size selection, we used parameters optimized for 250 bp insert size distribution. 10 cycles were performed in the final library amplification. Distribution and concentration of library was assessed using the Agilent 2100 Bioanalyzer instrument using a High Sensitivity DNA Assay. Libraries were pooled and paired-end sequenced in an Illumina NovaSeq instrument (Cancer Research UK Cambridge Institute).

##### 4tU labeling, RNA extraction, rRNA depletion

Three independent biological replicates of WT or Rpb7^QHF^ cells were grown in YEPD and labeled with 5 mM 4-thiouracil (Sigma, 440736-1G) for 6 minutes as previously described.^43^ RNA extraction was carried out as previously described^43^ with some minor modifications. Briefly, six *S. pombe* spike-in aliquots were resuspended in 6 ml of TRI reagent (ThermoFisher, AM9738) and aliquoted across three biological replicates of WT or Rpb7^QHF^ cells to have equally distributed spike-in RNA across strains and replicates. RNA was chloroform extracted and isopropanol precipitated following TRI reagent instructions. Extracted RNA was DNaseI treated and purified by phenol-chroloform extraction and isopropanol precipitation. RNA quality (i.e. intact rRNA) was assessed on an agarose gel.

Ribosomal RNA (rRNA) was depleted using the Ribominus Transcriptome Isolation Kit (Yeast and Bacteria) (Invitrogen K1550-03) following the kit protocol. To purify the RiboMinus RNA, we followed the ethanol precipitation protocol included in the kit. Complete rRNA depletion was confirmed by analysis of RiboMinus RNA on an agarose gel.

##### First strand cDNA synthesis and qPCR

First strand cDNA synthesis was performed on total RNA (without rRNA depletion) for subsequent qPCR analysis as previously described,^43^ with minor modifications. Superscript IV Reverse Transcriptase (Invitrogen 18090010) was used to synthesize the first strand cDNA using 1 μg of total RNA obtained from three independent biological replicates of WT and Rpb7^QHF^ cells, and 2.5 μM oligo(dT)/random hexamer mix.

cDNA qPCR reactions (only from the total RNA fraction) was performed in technical triplicates, and analyzed as previously described^43^ with some modifications. Specifically, only the act-1 and gpd-3 *S. pombe* transcripts were used to calculate the spike-in value. The *S. pombe*-normalized values were plotted using Prism 9 (9.4.1).

##### 4tU-seq library preparation

Nascent RNA biotinylation and purification was performed as previously described^43^ with minor modifications. Briefly, 3 μg of RiboMinus rRNA-depleted RNA were used in the biotinylation reaction (0.2 mg/ml EZ-Link Biotin-HPDP in 10 mM HEPES pH 7.5, 1 mM EDTA). 4tU-seq libraries were prepared following the protocol for rRNA depleted RNA in the NebNext Ultra II Directional RNA-seq kit.

##### Processing of sequencing data

Paired-end sequencing reads were aligned to the yeast genome (R64-1-1) using Bowtie (v2.5.1) for the ChIP-seq data and STAR (v2.5.3a) for the 4tU-seq data. Aligned reads were filtered using samtools (v1.6) with the following parameters (-b -h -q 7 -f 3 -F 1804). Replicates were manually inspected for overall reproducibility and subsequently merged to facilitate visualization using samtools merge (-c -r -f). Reads were sorted and indexed using samtools sort and samtools index, respectively. Strand-specific 4tU-seq tracks were generated with deeptools (v3.5.1) bamCoverage (--normalizeUsing CPM --binSize 10 --smoothLength 20 --filterRNAstrand forward (reverse)). Log_2_ IP/input was calculated using deeptools bamCompare implementing the SES scaling method^101^ (--scaleFactorsMethod SES -l 100 -n 100000 –operation log_2_ --pseudocount 1 –binSize 10 --smoothLength 20).

##### Sequencing data analysis

To visualize RNA-seq and ChIP-seq data across the genome we used Integrated Genome Viewer (v.2.4.11). We used Seq-Plots (v3.0.12) to perform metagene plots and K-means clustering of the 4tU-seq data past the TES. MACS2 (v2.2.6) was used to identify RNA Pol II peaks in WT and Rpb7^QHF^ cells, and DiffBind (v3.10.0)^92^ was used to determine differential enrichment analysis. RNA Pol II retention was calculated and visualized in R (v4.3.0). To calculate the RNA Pol II retention index we used deeptools multiBigWig summary to calculated the average RNA Pol II signal over a 300 bp window immediately upstream and 300 bp downstream of the TES. The average RNA Pol II signal over every annotated ORF-T in WT and Rpb7^QHF^ cells was also calculated using deeptools mltiBigWig summary and the difference (Change in RNA Pol II) calculated in R.

#### Crystallization and X-ray crystallography

The chimeric protein Glc7*–*Ref2_348-406_–GSGSG–2x(SPSYSPT) was diluted to 7 mg/ml in 10 mM Na-HEPES pH 8.0, 150 mM NaCl, 0.5 mM TCEP, 5% glycerol, and crystallization screens were set up using the MRC-LMB crystallization facility. Initial crystal hits were observed from experiments performed in 200 nl vapour diffusion sitting drops set up by a Mosquito Crystal nanodispenser (TTP Labtech) in MRC-2 well plates (Swissci, Hampton research). The best crystals grew in 40 % (v/v) 1,4-Butanediol and 0.1 M Tris-HCl pH 8.5, which could be reproduced manually in hanging drop consisting of 0.5 μl reservoir + 0.5 μl protein drop against 1 ml reservoir solution. Crystals were harvested and cryo-cooled without adding any cryoprotectant.

X-ray data were collected on beamline I04 at Diamond Light Source. 1800 images were recorded on a Dectris Eiger2 XE 16M detector using an oscillation range of 0.1° and an exposure time of 0.05 s per image (40% of beam transmission) at a temperature of 100 K. Datasets were initially processed with the Xia2^72^ automated pipeline, using XDS^73^ for indexing and integration, and XSCALE^74^ for scaling and merging. Data were cut at 1.85 Å resolution based on CC_1/2_ of 0.9 and I/σ of 1.84 in the highest resolution shell. The structure of Glc7*–*Ref2_348-406_–GSGSG–2x(SPSYSPT) was solved by molecular replacement in Phaser^75^ using apo-Glc7 (PDB: 7QWJ) as a search model. The Ref2 fragment was interactively built *de novo* in COOT^80^ into the F_o_-F_c_ difference map, followed by iterative cycles of refinement in phenix.refine^76^ and Refmac,^77^ and model adjustments in COOT. The final model was validated with MolProbity.^78^

The buried surface area between Ref2 and Glc7 was calculated with the PDBePISA tool.^79^ Images were prepared with UCSF ChimeraX^81^ and PyMOL (The PyMOL Molecular Graphics System, Version 2.3.5, Schrödinger, LLC). Overlay of the Glc7*–*Ref2_348-406_ crystal structure to that of Glc7 and PP1, and calculation of their root-mean-square deviation (RMSD) of Cα were performed with PDBeFold.^102^

Protein sequence alignments of Ref2 orthologs in Figure 5D were conducted by Clustal Omega (RRID:SCR_001591). The structural features of the RVxF motif in human PNUTS and fission yeast PNUTS (Ppn1 or C74.02c, Uniprot: 074535) were predicted based on AlphaFold2.^52^^,^^53^

#### Design of the DNA–RNA hybrid scaffolds

Nucleic acid scaffolds mimicking a transcription elongation bubble were designed for structural and functional studies based on sequences used in previous studies^39^ (Figure S2; Table S1). All nucleotides were synthesized by Integrated DNA Technologies. For the transcription assays, a different DNA scaffold was used that annealed to the *CYC1d* sub-fragment of *CYC1*,^36^ and it encoded the downstream sequence of *CYC1* (Figure S2A). For the RNA cleavage assays, we modified the DNA scaffold to anneal to the 3′-UTR of the *in vitro* transcribed *CYC1* model pre-mRNA^103^ (Figure S2B). For the EM studies on the RNA Pol II–Ref2–Glc7–Swd2 complex, we used the scaffold reported in Ehara et al.^39^ (Figure S2C) with no further modifications. For the RNA Pol II–APT EM studies, the scaffold from Ehara and colleagues was modified to anneal to the small-nucleolar RNA *snR47*, whose processing has been linked to APT^25^ (Figure S2C). All the template strand (TS) and the non-template strand (NTS) DNAs were designed to have an 11 bp mismatch, to allow the formation of a stable 9 nt DNA:RNA hybrid in the transcription bubble.^68^

The RNA was first annealed to TS-DNA at 40 μM concentration in 20 mM Tris-HCl pH 7.5, 4 mM Mg(OAc)_2_, and 150 mM KAc in 1.5 ml Eppendorf amber tubes to protect the fluorophore. The reaction was heated to 95 °C for 5 minutes, and then allowed to gradually cool at room temperature. The TS-DNA–RNA scaffold was subsequently mounted step-wise on hyperphosphorylated RNA Pol II.^68^ RNA Pol II was incubated with 1.5 molar excess of the TS-DNA–RNA hybrid in 10 mM K-HEPES pH 8.0, 100 mM KCl and 0.5 mM TCEP for 10 minutes at 30 °C with gentle shaking (300 rpm). An equal amount of NTS-DNA strand was added to the reaction, and the 10-minute incubation was repeated. After centrifugation for 5 minutes at 21,130 x g, the sample was subjected to gel filtration in 10 mM K-HEPES pH 8.0, 100 mM KCl, 0.5 mM TCEP and 5 % glycerol to remove the unbound nucleic acids. Peak fractions with an A_260/280nm_ ∼1 were pooled, concentrated, and stored as DNA–RNA-loaded RNA Pol II to be utilized in EM (see EM methods).

#### RNA Pol II *in vitro* transcription assay

For the RNA extension assays in the presence of APT and CPF we employed the DNA-RNA hybrid displayed in Figure S2A and Table S1. Hyperphosphorylated RNA Pol II was assembled with the scaffold as detailed above and subjected to gel filtration. 150 nM DNA–RNA-loaded RNA Pol II was pre-incubated with an excess APT or CPF (1 μM), then incubated with a 0.5 mM mixture of nucleotide triphosphates (NTPs) in 50 μl reactions in transcription buffer (20 mM Tris-HCl pH 8.0, 150 mM KAc, 5 mM Mg(OAc)_2_. 5 μl fractions were taken over time to monitor the progression of transcription, and quenched in a stop solution (130 mM EDTA, 5% (w/v) SDS, and 12 mg/ml proteinase K) at 37 °C for 10 minutes. 2 x denaturing loading dye (95% formamide, 10 mM EDTA, 0.01% w/v bromophenol blue) was added to the samples before loading onto 20% TBE (Tris-borate-EDTA)-polyacrylamide gels containing 7 M urea. The gels were run at 400 V in 1 x TBE running buffer for 45 minutes and were scanned with an Amersham™ Typhoon™ Biomolecular Imager for 5′ 6-FAM labelled *CYC1* RNA with excitation at 488 nm.

#### RNA *in vitro* cleavage assay

CPF cleavage assays were performed at 30 °C in a reaction buffer containing 10 mM K-HEPES pH 8.0, 100 mM KCl and 0.5 mM TCEP on 100 nM of the 259-nt *CYC1* pre-mRNA annealed to an artificial transcription bubble as described above (Figure S2B), in the absence or in the presence of RNA Pol II. Purified recombinant CPF was added to a final concentration of 150 nM in the reaction. The cleavage factors, CF IA and CF IB were diluted to 15 μM in 20 mM Na-HEPES pH 8.0, 250 mM NaCl, and 0.5 mM TCEP and added to a final concentration of 450 nM in the reaction. Reactions were stopped at different time points as indicated, by incubation for 10 minutes at 37 °C in a stop solution containing 130 mM EDTA, 5% (w/v) SDS, and 12 mg/ml proteinase K in reaction buffer. 2 x denaturing loading dye (95% formamide, 10 mM EDTA, 0.01% w/v bromophenol blue) was added to the samples before loading onto 15% TBE (Tris-borate-EDTA)-polyacrylamide gels containing 7 M urea. The gels were run at 400 V in 1 x TBE running buffer, stained with SYBR Green (Invitrogen S7564), and imaged using an Amersham™ Typhoon™ Biomolecular Imager.

#### Negative stain electron microscopy

##### Specimen preparation

To compare the extent of RNA Pol II dimerization under different conditions we used negative stain electron microscopy. 200 μg DNA–RNA-loaded RNA Pol II (Figure S2C) was hyperphosphorylated and subjected to SEC in 10 mM K-HEPES pH 8.0, 100 mM KCl and 0.5 mM TCEP as reported above. After concentration, 50 μg hyperphosphorylated RNA Pol II was incubated with with 1 μM of either CPF or APT in a total volume of 50 μl for 90 minutes at 30 °C in phosphatase buffer (20 mM K-HEPES pH 8.0, 150 mM KCl, 0.5 mM Mg(OAc)_2_, 0.5 mM MnCl_2_, 2 mM DTT). For the same set of experiments, 50 μg hyperphosphorylated RNA Pol II was was dephosphorylated with 250 units of λ phosphatase (NEB, cat. No. P0753L) supplemented with PMP buffer and MnCl_2_ in a total 50 μl volume for 30 minutes at 30 °C. RNA Pol II-ΔCTD was generated by incubation of unphosphorylated RNA Pol II with 2.5 μg 3C protease in 50 μl. Purified RNA Pol II Δ-stalk was incubated with 3 molar excess of recombinant Rpb4–Rpb7 (wild type or mutant). After treatment, each RNA Pol II-containing reaction was loaded on gel filtration (Superose 6 3.2/300 equilibrated in 10 mM K-HEPES pH 8.0, 100 mM KCl and 0.5 mM TCEP) in order to remove excess reagents, monitor RNA Pol II integrity, and apply the same buffer to all conditions being compared. Peak fractions were pooled, and RNA Pol II diluted to 20 nM in 10 mM K-HEPES pH 8.0, 100 mM KCl, 0.5 mM TCEP and 0.005 % v/v Tween-20) for negative stain specimen preparation. 3 μl sample was applied to copper 400-mesh grids with an ultra-thin layer of carbon (Electron Microscopy Sciences, cat. No. CF400-Cu-UL), that had been glow-discharged in air for 30 seconds. The sample was incubated on the support for 60 seconds and then wicked with filter paper. Grids were sequentially applied to three 30 μl drops of 2% w/v uranyl acetate (7 seconds floating on top of each drop), and the excess stain removed by filter paper.

##### Data collection and processing

Grids were stored until imaging on a Tecnai Spirit microscope (FEI) operated at 120 keV, equipped with an Orius SC200W CCD camera (Gatan). 50-60 micrographs for each RNA Pol II treatment were collected at a magnification of 21,000 X (2.53 Å/pixel), at -1 μm defocus and a total electron dose of 40-60 e^-^/A^2^ over 2 seconds. For each micrograph, total particle auto-picking was performed with Laplacian-of-Gaussian (LoG) in Relion (v3.1),^104^ using a 300 Å-wide area, which can accommodate RNA Pol II dimers (230 Å through the longest axis). Automated picking was manually inspected to check the quality of the particles considered as ‘dimeric, using the 2D-projections of the cryoEM RNA Pol II dimer as a reference (Figure S8C). Trimeric particles, if present, were also picked as dimers. For each RNA Pol II sample, the ratio of dimeric particles over the total number of particles per micrograph was determined and plotted to calculate means and standard deviations using Graphpad Prism 9. Means were used for pairwise comparison between treatments, and statistical significance assessed by one-way ANOVA Tukey’s test. The quantifications in the left and middle panels of Figure 4 were performed twice in ‘double-blind’ replicates.

#### Single-particle electron cryo-microscopy

##### Specimen preparation and data collection

To stabilize the DNA–RNA-loaded RNA Pol II (scaffold in Figure S2C) in the presence of APT, we utilized gradient centrifugation coupled to cross-linking (GraFix).^37^ A 10-40 % sucrose gradient in the presence of 0.1 % glutaraldehyde (Figure S3A) was generated with a Biocomp Gradient Station. 200 ng sample in 100 μl was pipetted on top of the gradient before ultracentrifugation at 33,000 rpm in a SW40 Ti Beckman rotor at 4 °C for 18 hours. The sample was then manually fractionated, quenched in 100 mM Tris-HCl pH 8.0 and analyzed on a NuPAGE 3-8 % Tris-Acetate gel (Invitrogen). Fractions containing protein bands corresponding to the expected size of a RNA Pol II–APT complex were dialyzed against 10 mM K-HEPES pH 8.0, 100 mM KCl and 0.5 mM TCEP (final concentration of 200-300 nM).

For RNA Pol II–Ref2–Glc7–Swd2, 2.5 μM DNA–RNA-loaded RNA Pol II (Figure S2C) was assembled with 1.8 molar excess of Ref2–Glc7–Swd2 and incubated on ice for 30 minutes in 50 μl. After spinning at 21,130 x g, the supernatant was injected on a Superose 6 3.2/300 in 10 mM K-HEPES pH 8.0, 100 mM KCl and 0.5 mM TCEP. Pooled fractions were concentrated in Vivaspin 500 (Sartorious, cat. No. VS0121) to 2 μM. 0.005 % v/v Tween-20 was added just before vitrification.

RNA Pol II Δ-stalk and RNA Pol II Δ-CTD were prepared in the same conditions detailed above in the ‘negative stain EM’ section, and concentrated to 2 μM. 0.005 % v/v Tween-20 was added just before vitrification.

For all protein complexes and RNA Pol II deletions, 3 μl sample was applied to 1.2/1.3 or 0.6/1 UltrAuFoil grids (Quantifoil)^105^ made hydrophilic using a 9:1 Argon:Oxygen plasma for 45 seconds. The applied sample was blotted for 5 seconds with a blot force of -12 at 4 °C and 100 % humidity, and plunged into liquid ethane on a Vitrobot Mark IV (FEI). Grids were imaged on Titan Krios microscopes operated at 300 keV using the EPU automated data collection software. Total dose (40 e^-^/Å^2^) and defocus range (-1.5 μm to -2.7) were kept consistent throughout datasets and microscopes.

For RNA Pol II–APT, 7,980 movies were collected on a Gatan K3 detector in super-resolution mode, with a pixel size of 0.83 Å/pixel on Krios III at eBIC.

For RNA Pol II–Ref2–Glc7–Swd2, a total of 27,500 movies were recorded. Initial datasets with stage tilt (0 °, 30 ° and 40°) were collected on Krios I (MRC-LMB) with a Falcon III detector in counting mode, with a pixel size of 1.04 Å/pixel. Data at zero tilt were collected at the University of Cambridge with a Gatan K3 detector in super-resolution mode, with a pixel size of 0.83 Å/pixel. Data with a tilted stage (33°, 40 ° and 45°) were acquired at MRC-LMB on Krios III with a Gatan K3 detector in counting mode, pixel size of 0.86 Å/pixel.

For RNA Pol II Δ-stalk and RNA Pol II Δ-CTD, 1,200 movies for each condition (containing ∼400,000 particles) were collected on Krios I (MRC-LMB) with a Falcon III detector in integrating mode, with a pixel size of 1.04 Å/pixel.

##### Data processing

For all samples, the stack of frames from the movies were corrected for whole-frame motion using Relion’s version of MotionCor2,^106^ and the defocus was estimated using CTFFIND4.^107^ A flowchart for the RNA Pol II–APT processing is shown in Figure S3C. After LoG-based particle picking in Relion v3.1, particles were subjected to two rounds of 2D classification followed by consensus 3D refinement and 3D classification. Despite achieving high resolution for apo RNA Pol II, 3D classes displaying additional density for APT were structurally heterogeneous. We therefore carried out extensive processing in both Relion and cryoSPARC (not shown), and obtained similar results. In Relion, we attempted focused 3D classification followed by focused refinement of the APT density after signal subtraction of RNA Pol II. Different values of the T regularization parameter were employed during 3D classification with or without image alignment. Because the APT density did not seem to improve, we repeated the same procedure without signal subtraction, by shifting the mask over the region of interest. In cryoSPARC, we used non-uniform refinement, but this also gave poor resolution for the density that we assign to APT.

For RNA Pol II–Ref2–Glc7–Swd2, all seven collected datasets were treated separately for initial processing. The best particles were merged only after 2D classification, keeping Falcon III and K3 data separate, as described in the processing schematic (Figure S5). Image processing was performed in Relion (v3.1)^104^ and Relion wrappers were used for external programs except for crYOLO. Particles were picked using a general network model in crYOLO.^108^ For each dataset, three rounds of 2D classification on 3.5x binned particles were performed to remove junk particles. High-resolution classes containing both RNA Pol II monomers and dimers were then merged and used to generate an initial 3D model. The initial model was used as a reference for one round of 3D classification to separate monomers from dimers. The RNA Pol II dimer classes were subjected to 3D refinement with no symmetry applied. Particles were unbinned and the Falcon III and K3 datasets were merged with a consensus pixel size of 0.83 Å^2^/pixel. Merged particles were refined further resulting in a 3D reconstruction of the RNA Pol II dimer with a global resolution of 4.6 Å.

Focused refinement on each monomer was performed after signal subtraction from the other monomer. The two separately focus-refined monomers showed no noticeable difference between each other. Therefore, to further improve the resolution of our map, we applied ‘local symmetry’,^109^ whereby particles from each monomeric subunit of the dimer were merged after signal subtraction of the other subunit. We refined this merged particle stack against the ‘focus-refined’ map of either monomer yielding 3.6 Å maps irrespective of which monomer was used as the reference. Maps were post-processed using negative B-factor values calculated by Relion. Map validation according to the gold-standard FSC at 0.143, and local resolutions maps are displayed in Figure S6. We calculated directional FSC plots and sphericity values using the 3D-FSC server.^93^

Two copies of a monomeric RNA Pol II crystal structure^42^ were manually fitted into the EM density of the RNA Pol II dimer and then subjected to rigid fitting in USCF Chimera.^110^ Illustrations and movies were prepared using UCSF ChimeraX^81^ and PyMOL (The PyMOL Molecular Graphics System, Version 2.3.5, Schrödinger, LLC). The buried surface area between the Rpb7 subunits within the RNA Pol II crystallographic dimer (PDB: 5C4X) was calculated in PyMOL with the ‘get_area’ command.

#### Dynamic light scattering

To analyze the concentration-dependent dimerization of RNA Pol II, RNA Pol II-Δstalk and RNA Pol II-ΔCTD were analysed by DLS. DNA–RNA hybrid-loaded RNA Pol II (Figure S2C) was serially diluted from 5 μM to 156 nM in 10 mM K-HEPES pH 8.0, 100 mM KCl and 0.5 mM TCEP. 25 μl of each dilution was applied to a 384-well black/clear bottom Polystyrene microplate (Corning, cat No. 3540), followed by centrifugation to remove air bubbles or aggregates in the samples (2 minutes, 4,000 x g). 40 consecutive DLS measurements were acquired for each concentration point on a DynaPro plate reader (Wyatt Technology) at 25 °C. Data were analyzed directly in the accompanying DYNAMICS® software, and an autocorrelation function calculated for each measurement. The translational diffusion coefficient was obtained through automated nonlinear least squares fitting of the autocorrelation function, and the hydrodynamic radius (R_h_) derived through the Stokes-Einstein equation. Visualization of the R_h_ as a function of protein concentration was carried out in GraphPad Prism version 9.0. R_h_ values deriving from DLS measurements with weak scattering signal or showing multimodal distribution (on average 7 % per sample) were excluded from the plots.

#### SEC multiangle light scattering

The mass in solution of both wild type and mutant Rpb4–Rpb7 (RNA Pol II stalk) was determined by SEC-MALS measurements using a Wyatt Heleos II 18 angle light scattering instrument coupled to a Wyatt Optilab rEX online refractive index detector. Protein samples (100 μl at 2 mg/ml) were resolved using a Superdex 200 10/300 analytical gel filtration column (GE Healthcare) running at 0.5 ml/min at 25 °C in 10 mM K-HEPES pH 8.0, 100 mM KCl and 0.5 mM TCEP. Protein concentration was determined from the excess differential refractive index (RI) based on 0.186 RI increment for 1 g/ml protein solution. The concentration and the observed scattered intensity at each point in the chromatograms were used to calculate the absolute molecular mass from the intercept of the Debye plot using the Zimm model as implemented in Wyatt’s ASTRA software.

#### *In vitro* phase separation and microscopy

Droplet formation occurs upon mixing 0.6 μM DNA–RNA-loaded RNA Pol II with ≥ 4 μM APT (titration experiment, not shown). To follow the distribution of RNA Pol II and RNA in the condensates, in the presence or in the absence of APT, we used confocal microscopy. The RNA annealed to the nucleic acid scaffold was labelled at the 5ʹ with fluorescein (5′ 6-FAM) (Integrated DNA Technologies). For RNA Pol II, we took advantage of the Rpb3-His_6_-Bio tag carrying a biotinylation acceptor peptide, which can be biotinylated *in vitro* by incubation with bacterial BirA biotin ligase (Merck, cat. No. SRP0717). Biotinylation enabled us to label RNA Pol II via Streptavidin-conjugated Alexa Fluor™ 647 (ThermoFisher, cat. No. S21374). The droplets were allowed to form for 2 minutes in a 0.5 ml Protein LoBind Eppendorf tube in 10 mM K-HEPES pH 8.0, 100 mM KCl and 0,5 mM TCEP. 4 μl of the reaction was transferred to buffer-rinsed 50-well CultureWell chambered coverslides (Grace Bio-Labs, cat. No. GBL103350), and sealed with crystal clear tape. Imaging was conducted on a Zeiss LSM 780 laser scanning confocal microscope with a Plan-Apochromat 20x/0.8 M27 objective, by focusing on the bottom of the coverslide, where droplets settle. The sample was imaged with an excitation wavelength of 488 nm and 633 nm for FAM-RNA and RNA Pol II-Alexa Fluor 647, respectively. Images were further processed and merged in FIJI (version 2.0.0-rc-66/1.52b).

### Quantification and statistical analysis

Statistical analyses were performed using Prism (Figures 3G, 4B, 4C, 5F, S9C, S9D, and S10E), R (Figure 4E) and SeqPlots (Figures 4F, S9A, and S9F–S9H). Details on number of replicates or number of genes, error estimate, statistical tests, and significance cutoff can be found in the respective figure legends.

## Supplemental information

Document S1. Figures S1–S12, Table S1, and supplemental references

Document S2. Article plus supplemental information

## References

[1] Hirose Y., Manley J.L. (1998). RNA polymerase II is an essential mRNA polyadenylation factor. Nature.

[2] Hirose Y., Tacke R., Manley J.L. (1999). Phosphorylated RNA polymerase II stimulates pre-mRNA splicing. Genes Dev..

[3] Mischo H.E., Proudfoot N.J. (2013). Disengaging polymerase: terminating RNA polymerase II transcription in budding yeast. Biochim. Biophys. Acta.

[4] Bentley D.L. (2014). Coupling mRNA processing with transcription in time and space. Nat. Rev. Genet..

[5] Herzel L., Ottoz D.S.M., Alpert T., Neugebauer K.M. (2017). Splicing and transcription touch base: co-transcriptional spliceosome assembly and function. Nat. Rev. Mol. Cell Biol..

[6] Zhao J., Hyman L., Moore C. (1999). Formation of mRNA 3′ ends in eukaryotes: mechanism, regulation, and interrelationships with other steps in mRNA synthesis. Microbiol. Mol. Biol. Rev..

[7] Ghosh A., Shuman S., Lima C.D. (2011). Structural insights to how mammalian capping enzyme reads the CTD code. Mol. Cell.

[8] Komarnitsky P., Cho E.J., Buratowski S. (2000). Different phosphorylated forms of RNA polymerase II and associated mRNA processing factors during transcription. Genes Dev..

[9] Nojima T., Rebelo K., Gomes T., Grosso A.R., Proudfoot N.J., Carmo-Fonseca M. (2018). RNA polymerase II phosphorylated on CTD serine 5 interacts with the spliceosome during co-transcriptional splicing. Mol. Cell.

[10] Vasiljeva L., Kim M., Mutschler H., Buratowski S., Meinhart A. (2008). The Nrd1-Nab3-Sen1 termination complex interacts with the Ser5-phosphorylated RNA polymerase II C-terminal domain. Nat. Struct. Mol. Biol..

[11] Mayer A., Heidemann M., Lidschreiber M., Schreieck A., Sun M., Hintermair C., Kremmer E., Eick D., Cramer P. (2012). CTD tyrosine phosphorylation impairs termination factor recruitment to RNA polymerase II. Science.

[12] Porrua O., Libri D. (2015). Transcription termination and the control of the transcriptome: why, where and how to stop. Nat. Rev. Mol. Cell Biol..

[13] Licatalosi D.D., Geiger G., Minet M., Schroeder S., Cilli K., McNeil J.B., Bentley D.L. (2002). Functional interaction of yeast pre-mRNA 3′ end processing factors with RNA polymerase II. Mol. Cell.

[14] Casañal A., Kumar A., Hill C.H., Easter A.D., Emsley P., Degliesposti G., Gordiyenko Y., Santhanam B., Wolf J., Wiederhold K. (2017). Architecture of eukaryotic mRNA 3′-end processing machinery. Science.

[15] Chanfreau G., Noble S.M., Guthrie C. (1996). Essential yeast protein with unexpected similarity to subunits of mammalian cleavage and polyadenylation specificity factor (CPSF). Science.

[16] Kumar A., Clerici M., Muckenfuss L.M., Passmore L.A., Jinek M. (2019). Mechanistic insights into mRNA 3′-end processing. Curr. Opin. Struct. Biol..

[17] Sun Y., Hamilton K., Tong L. (2020). Recent molecular insights into canonical pre-mRNA 3′-end processing. Transcription.

[18] Xiang K., Nagaike T., Xiang S., Kilic T., Beh M.M., Manley J.L., Tong L. (2010). Crystal structure of the human symplekin-Ssu72-CTD phosphopeptide complex. Nature.

[19] Singh B.N., Hampsey M. (2007). A transcription-independent role for TFIIB in gene looping. Mol. Cell.

[20] Allepuz-Fuster P., O'Brien M.J., González-Polo N., Pereira B., Dhoondia Z., Ansari A., Calvo O. (2019). RNA polymerase II plays an active role in the formation of gene loops through the Rpb4 subunit. Nucleic Acids Res..

[21] Tan-Wong S.M., Zaugg J.B., Camblong J., Xu Z., Zhang D.W., Mischo H.E., Ansari A.Z., Luscombe N.M., Steinmetz L.M., Proudfoot N.J. (2012). Gene loops enhance transcriptional directionality. Science.

[22] Schreieck A., Easter A.D., Etzold S., Wiederhold K., Lidschreiber M., Cramer P., Passmore L.A. (2014). RNA polymerase II termination involves C-terminal-domain tyrosine dephosphorylation by CPF subunit Glc7. Nat. Struct. Mol. Biol..

[23] Cortazar M.A., Sheridan R.M., Erickson B., Fong N., Glover-Cutter K., Brannan K., Bentley D.L. (2019). Control of RNA Pol II Speed by PNUTS-PP1 and Spt5 dephosphorylation facilitates termination by a "sitting duck torpedo" mechanism. Mol. Cell.

[24] Kecman T., Kuś K., Heo D.H., Duckett K., Birot A., Liberatori S., Mohammed S., Geis-Asteggiante L., Robinson C.V., Vasiljeva L. (2018). Elongation/termination factor exchange mediated by PP1 phosphatase orchestrates transcription termination. Cell Rep..

[25] Lidschreiber M., Easter A.D., Battaglia S., Rodríguez-Molina J.B., Casañal A., Carminati M., Baejen C., Grzechnik P., Maier K.C., Cramer P., Passmore L.A. (2018). The APT complex is involved in non-coding RNA transcription and is distinct from CPF. Nucleic Acids Res..

[26] Nedea E., Nalbant D., Xia D., Theoharis N.T., Suter B., Richardson C.J., Tatchell K., Kislinger T., Greenblatt J.F., Nagy P.L. (2008). The Glc7 phosphatase subunit of the cleavage and polyadenylation factor is essential for transcription termination on SnoRNA genes. Mol. Cell.

[27] Connelly S., Manley J.L. (1988). A functional mRNA polyadenylation signal is required for transcription termination by RNA polymerase II. Genes Dev..

[28] Logan J., Falck-Pedersen E., Darnell J.E., Shenk T. (1987). A poly(A) addition site and a downstream termination region are required for efficient cessation of transcription by RNA polymerase II in the mouse beta maj-globin gene. Proc. Natl. Acad. Sci. USA.

[29] Baejen C., Andreani J., Torkler P., Battaglia S., Schwalb B., Lidschreiber M., Maier K.C., Boltendahl A., Rus P., Esslinger S. (2017). Genome-wide analysis of RNA polymerase II termination at protein-coding genes. Mol. Cell.

[30] Dantonel J.C., Murthy K.G., Manley J.L., Tora L. (1997). Transcription factor TFIID recruits factor CPSF for formation of 3′ end of mRNA. Nature.

[31] Kim M., Krogan N.J., Vasiljeva L., Rando O.J., Nedea E., Greenblatt J.F., Buratowski S. (2004). The yeast Rat1 exonuclease promotes transcription termination by RNA polymerase II. Nature.

[32] West S., Gromak N., Proudfoot N.J. (2004). Human 5′ --> 3’ exonuclease Xrn2 promotes transcription termination at co-transcriptional cleavage sites. Nature.

[33] Richard P., Manley J.L. (2009). Transcription termination by nuclear RNA polymerases. Genes Dev..

[34] West M.L., Corden J.L. (1995). Construction and analysis of yeast RNA polymerase II CTD deletion and substitution mutations. Genetics.

[35] Allepuz-Fuster P., Martínez-Fernández V., Garrido-Godino A.I., Alonso-Aguado S., Hanes S.D., Navarro F., Calvo O. (2014). Rpb4/7 facilitates RNA polymerase II CTD dephosphorylation. Nucleic Acids Res..

[36] Hill C.H., Boreikaitė V., Kumar A., Casañal A., Kubík P., Degliesposti G., Maslen S., Mariani A., von Loeffelholz O., Girbig M. (2019). Activation of the endonuclease that defines mRNA 3′ ends requires incorporation into an 8-subunit core cleavage and polyadenylation factor complex. Mol. Cell.

[37] Kastner B., Fischer N., Golas M.M., Sander B., Dube P., Boehringer D., Hartmuth K., Deckert J., Hauer F., Wolf E. (2008). GraFix: sample preparation for single-particle electron cryomicroscopy. Nat. Methods.

[38] Davis J.A., Takagi Y., Kornberg R.D., Asturias F.A. (2002). Structure of the yeast RNA polymerase II holoenzyme: mediator conformation and polymerase interaction. Mol. Cell.

[39] Ehara H., Yokoyama T., Shigematsu H., Yokoyama S., Shirouzu M., Sekine S.I. (2017). Structure of the complete elongation complex of RNA polymerase II with basal factors. Science.

[40] Lahiri I., Xu J., Han B.G., Oh J., Wang D., DiMaio F., Leschziner A.E. (2019). 3.1 A structure of yeast RNA polymerase II elongation complex stalled at a cyclobutane pyrimidine dimer lesion solved using streptavidin affinity grids. J. Struct. Biol..

[41] Aibara S., Dienemann C., Cramer P. (2021). Structure of an inactive RNA polymerase II dimer. Nucleic Acids Res..

[42] Barnes C.O., Calero M., Malik I., Graham B.W., Spahr H., Lin G., Cohen A.E., Brown I.S., Zhang Q., Pullara F. (2015). Crystal structure of a transcribing RNA polymerase II complex reveals a complete transcription bubble. Mol. Cell.

[43] Rodríguez-Molina J.B., O'Reilly F.J., Fagarasan H., Sheekey E., Maslen S., Skehel J.M., Rappsilber J., Passmore L.A. (2022). Mpe1 senses the binding of pre-mRNA and controls 3′ end processing by CPF. Mol. Cell.

[44] Peti W., Nairn A.C., Page R. (2013). Structural basis for protein phosphatase 1 regulation and specificity. FEBS Journal.

[45] Fedoryshchak R.O., Přechová M., Butler A.M., Lee R., O'Reilly N., Flynn H.R., Snijders A.P., Eder N., Ultanir S., Mouilleron S. (2020). Molecular basis for substrate specificity of the Phactr1/PP1 phosphatase holoenzyme. eLife.

[46] Werner-Allen J.W., Lee C.J., Liu P., Nicely N.I., Wang S., Greenleaf A.L., Zhou P. (2011). cis-proline-mediated Ser(P)5 dephosphorylation by the RNA polymerase II C-terminal domain phosphatase Ssu72. J. Biol. Chem..

[47] Tanaka S., Miyazawa-Onami M., Iida T., Araki H. (2015). iAID: an improved auxin-inducible degron system for the construction of a 'tight' conditional mutant in the budding yeast Saccharomyces cerevisiae. Yeast.

[48] Russnak R., Nehrke K.W., Platt T. (1995). REF2 encodes an RNA-binding protein directly involved in yeast mRNA 3′-end formation. Mol. Cell. Biol..

[49] Strzyz P. (2018). Concentrating on intrinsic disorder. Nat. Rev. Genet..

[50] Choy M.S., Hieke M., Kumar G.S., Lewis G.R., Gonzalez-DeWhitt K.R., Kessler R.P., Stein B.J., Hessenberger M., Nairn A.C., Peti W. (2014). Understanding the antagonism of retinoblastoma protein dephosphorylation by PNUTS provides insights into the PP1 regulatory code. Proc. Natl. Acad. Sci. USA.

[51] Shi Y., Di Giammartino D.C., Taylor D., Sarkeshik A., Rice W.J., Yates J.R., Frank J., Manley J.L. (2009). Molecular architecture of the human pre-mRNA 3′ processing complex. Mol. Cell.

[52] Varadi M., Anyango S., Deshpande M., Nair S., Natassia C., Yordanova G., Yuan D., Stroe O., Wood G., Laydon A. (2022). AlphaFold Protein Structure Database: massively expanding the structural coverage of protein-sequence space with high-accuracy models. Nucleic Acids Res..

[53] Jumper J., Evans R., Pritzel A., Green T., Figurnov M., Ronneberger O., Tunyasuvunakool K., Bates R., Žídek A., Potapenko A. (2021). Highly accurate protein structure prediction with AlphaFold. Nature.

[54] Cermakova K., Demeulemeester J., Lux V., Nedomova M., Goldman S.R., Smith E.A., Srb P., Hexnerova R., Fabry M., Madlikova M. (2021). A ubiquitous disordered protein interaction module orchestrates transcription elongation. Science.

[55] Russnak R., Pereira S., Platt T. (1996). RNA binding analysis of yeast REF2 and its two-hybrid interaction with a new gene product, FIR1. Gene Expr..

[56] Pearson E., Moore C. (2014). The evolutionarily conserved Pol II flap loop contributes to proper transcription termination on short yeast genes. Cell Rep..

[57] Martinez-Rucobo F.W., Kohler R., van de Waterbeemd M., Heck A.J., Hemann M., Herzog F., Stark H., Cramer P. (2015). Molecular basis of transcription-coupled Pre-mRNA capping. Mol. Cell.

[58] Garg G., Dienemann C., Farnung L., Schwarz J., Linden A., Urlaub H., Cramer P. (2023). Structural insights into human co-transcriptional capping. Mol. Cell.

[59] Zhang S., Aibara S., Vos S.M., Agafonov D.E., Lührmann R., Cramer P. (2021). Structure of a transcribing RNA polymerase II-U1 snRNP complex. Science.

[60] Boehning M., Dugast-Darzacq C., Rankovic M., Hansen A.S., Yu T., Marie-Nelly H., McSwiggen D.T., Kokic G., Dailey G.M., Cramer P. (2018). RNA polymerase II clustering through carboxy-terminal domain phase separation. Nat. Struct. Mol. Biol..

[61] Lu H., Yu D., Hansen A.S., Ganguly S., Liu R., Heckert A., Darzacq X., Zhou Q. (2018). Phase-separation mechanism for C-terminal hyperphosphorylation of RNA polymerase II. Nature.

[62] Nosella M.L., Forman-Kay J.D. (2021). Phosphorylation-dependent regulation of messenger RNA transcription, processing and translation within biomolecular condensates. Curr. Opin. Cell Biol..

[63] Rawat P., Boehning M., Hummel B., Aprile-Garcia F., Pandit A.S., Eisenhardt N., Khavaran A., Niskanen E., Vos S.M., Palvimo J.J. (2021). Stress-induced nuclear condensation of NELF drives transcriptional downregulation. Mol. Cell.

[64] Schilbach S., Aibara S., Dienemann C., Grabbe F., Cramer P. (2021). Structure of RNA polymerase II pre-initiation complex at 2.9 A defines initial DNA opening. Cell.

[65] Kumar A., Yu C.W.H., Rodríguez-Molina J.B., Li X.H., Freund S.M.V., Passmore L.A. (2021). Dynamics in Fip1 regulate eukaryotic mRNA 3′ end processing. Genes Dev..

[66] Schilbach S., Hantsche M., Tegunov D., Dienemann C., Wigge C., Urlaub H., Cramer P. (2017). Structures of transcription pre-initiation complex with TFIIH and Mediator. Nature.

[67] Heinemeyer W., Kleinschmidt J.A., Saidowsky J., Escher C., Wolf D.H. (1991). Proteinase yscE, the yeast proteasome/multicatalytic-multifunctional proteinase: mutants unravel its function in stress induced proteolysis and uncover its necessity for cell survival. EMBO J.

[68] Kireeva M.L., Komissarova N., Waugh D.S., Kashlev M. (2000). The 8-nucleotide-long RNA:DNA hybrid is a primary stability determinant of the RNA polymerase II elongation complex. J. Biol. Chem..

[69] Naydenova K., Russo C.J. (2017). Measuring the effects of particle orientation to improve the efficiency of electron cryomicroscopy. Nat Commun.

[70] Gasteiger E., Hoogland C., Gattiker A., Duvaud S., Wilkins M.R., Appel R.D., and Bairoch A. (2005). Protein Identification and Analysis Tools on the Expasy Server. In The Proteomics Protocols Handbook, Humana Press (2005). John M. Walker, ed. pp. 571-607

[71] Zivanov J., Nakane T., Forsberg B.O., Kimanius D., Hagen W.J.H., Lindahl E., Scheres S.H.W. (2018). New tools for automated high-resolution cryo-EM structure determination in RELION-3. Elife.

[72] Winter G., Lobley C.M., Prince S.M. (2013). Decision making in xia2. Acta Crystallogr. D Biol. Crystallogr..

[73] Kabsch W. (2010). Xds. Acta Crystallogr. D Biol. Crystallogr..

[74] Kabsch W. (2010). Integration, scaling, space-group assignment and post-refinement. Acta Crystallogr. D Biol. Crystallogr..

[75] McCoy A.J. (2007). Solving structures of protein complexes by molecular replacement with Phaser. Acta Crystallogr. D Biol. Crystallogr..

[76] Adams P.D., Afonine P.V., Bunkóczi G., Chen V.B., Davis I.W., Echols N., Headd J.J., Hung L.W., Kapral G.J., Grosse-Kunstleve R.W. (2010). Phenix: a comprehensive Python-based system for macromolecular structure solution. Acta Crystallogr. D Biol. Crystallogr..

[77] Murshudov G.N., Skubák P., Lebedev A.A., Pannu N.S., Steiner R.A., Nicholls R.A., Winn M.D., Long F., Vagin A.A. (2011). REFMAC5 for the refinement of macromolecular crystal structures. Acta Crystallogr. D Biol. Crystallogr..

[78] Williams C.J., Headd J.J., Moriarty N.W., Prisant M.G., Videau L.L., Deis L.N., Verma V., Keedy D.A., Hintze B.J., Chen V.B. (2018). MolProbity: more and better reference data for improved all-atom structure validation. Protein Sci..

[79] Krissinel E., Henrick K. (2007). Inference of macromolecular assemblies from crystalline state. J. Mol. Biol..

[80] Emsley P., Lohkamp B., Scott W.G., Cowtan K. (2010). Features and development of coot. Acta Crystallogr. D Biol. Crystallogr..

[81] Pettersen E.F., Goddard T.D., Huang C.C., Meng E.C., Couch G.S., Croll T.I., Morris J.H., Ferrin T.E. (2021). UCSF ChimeraX: structure visualization for researchers, educators, and developers. Protein Sci..

[82] Goujon M., McWilliam H., Li W., Valentin F. Squizzato S., Paern J., and Lopez R. (2010). A new bioinformatics analysis tools framework at EMBL-EBI. Nucleic Acids Res 38, W695-W699. 10.1093/nar/gkq313 PMC2896090 20439314

[83] Sievers F., Wilm A., Dineen D., Gibson T.J., Karplus K., Li W., Lopez R., McWilliam H., Remmert M., Soding J. (2011). Fast, scalable generation of high-quality protein multiple sequence alignments using Clustal Omega. Mol Syst Biol.

[84] Schindelin J., Arganda-Carreras I., Frise E., Kaynig V., Longair M. Pietzsch T., Preibisch S., Rueden C., Saalfeld S., Schmid B., et al., (2012). Fiji: an open-source platform for biological-image analysis. Nat Methods 9, 676-682. 10.1038/nmeth.2019 PMC3855844 22743772

[85] Robinson J.T., Thorvaldsdóttir H., Winckler W., Guttman M., Lander E.S., Getz G., and Mesirov J.P. (2012). Integrative genomics viewer. Nat Biotechnol, 2011. 29(1): p. 24-6. 10.1038/nbt.1754 PMC3346182 21221095

[86] Langmead B., Salzberg S.L. Fast gapped-read alignment with Bowtie 2. Nat Methods 9:357–359. 10.1038/nmeth.1923 PMC3322381 22388286

[87] Dobin A., Davis C.A., Schlesinger F., Drenkow J., Zaleski C., Jha S., Batut P., Chaisson M., Gingeras T.R. (2013). STAR: ultrafast universal RNA-seq aligner. Bioinformatics.

[88] Li H., Handsaker B., Wysoker A., Fennell T., Ruan J., Homer N., Marth G., Abecasis G., Durbin R., and 1000 Genome Project Data Processing Subgroup. (2016). The Sequence Alignment/Map format and SAMtools. Bioinformatics 25, 2078-2079. 10.1093/bioinformatics/btp352 PMC2723002 19505943

[89] Ramirez F., Ryan D.P., Gruning B., Bhardwaj V., Kilpert F., Richter A.S., Heyne S., Dundar F., Manke T. (2016). deepTools2: a next generation web server for deep-sequencing data analysis. Nucleic Acids Res.

[90] Stempor P., Ahringer J. (2016). SeqPlots - Interactive software for exploratory data analyses, pattern discovery and visualization in genomics. Wellcome Open Res.

[91] Zhang Y., Liu T., Meyer C.A., Eeckhoute J., Johnson D.S., Bernstein B.E., Nusbaum C., Myers R.M., Brown M., Li W., Liu S. (2008). Model-based analysis of ChIP-Seq (MACS). Genome Biol.

[92] Ross-Innes C.S., Stark R., Teschendorff A.E., Holmes K.A., Ali H.R., Dunning M.J., Brown G.D., Gojis O., Ellis I.O., Green A.R. (2012). Differential oestrogen receptor binding is associated with clinical outcome in breast cancer. Nature.

[93] Tan Y.Z., Baldwin P.R., Davis J.H., Williamson J.R., Potter C.S., Carragher B., Lyumkis D. (2017). Addressing preferred specimen orientation in single-particle cryo-EM through tilting. Nat. Methods.

[94] Krissinel E., Henrick K. (2005). LNBI.

[95] Laughery M.F., Wyrick J.J. (2019). Simple CRISPR-Cas9 genome editing in Saccharomyces cerevisiae. Curr. Protoc. Mol. Biol..

[96] Concordet J.P., Haeussler M. (2018). CRISPOR: intuitive guide selection for CRISPR/Cas9 genome editing experiments and screens. Nucleic Acids Res..

[97] Chen D.C., Yang B.C., Kuo T.T. (1992). One-step transformation of yeast in stationary phase. Curr. Genet..

[98] Weissmann F., Petzold G., VanderLinden R., Huis In 't Veld P.J., Brown N.G., Lampert F., Westermann S., Stark H., Schulman B.A., Peters J.M. (2016). biGBac enables rapid gene assembly for the expression of large multisubunit protein complexes. Proc. Natl. Acad. Sci. USA.

[99] Sydow J.F., Brueckner F., Cheung A.C., Damsma G.E., Dengl S., Lehmann E., Vassylyev D., Cramer P. (2009). Structural basis of transcription: mismatch-specific fidelity mechanisms and paused RNA polymerase II with frayed RNA. Mol. Cell.

[100] Kushnirov V.V. (2000). Rapid and reliable protein extraction from yeast. Yeast.

[101] Diaz A., Park K., Lim D.A., Song J.S. (2012). Normalization, bias correction, and peak calling for ChIP-seq. Stat. Appl. Genet. Mol. Biol..

[102] Krissinel E., Henrick K. (2004). Secondary-structure matching (SSM), a new tool for fast protein structure alignment in three dimensions. Acta Crystallogr. D Biol. Crystallogr..

[103] Butler J.S., Platt T. (1988). RNA processing generates the mature 3′ end of yeast CYC1 messenger RNA in vitro. Science.

[104] Zivanov J., Nakane T., Scheres S.H.W. (2019). A Bayesian approach to beam-induced motion correction in cryo-EM single-particle analysis. IUCrJ.

[105] Russo C.J., Passmore L.A. (2014). Electron microscopy: ultrastable gold substrates for electron cryomicroscopy. Science.

[106] Zheng S.Q., Palovcak E., Armache J.P., Verba K.A., Cheng Y., Agard D.A. (2017). MotionCor2: anisotropic correction of beam-induced motion for improved cryo-electron microscopy. Nat. Methods.

[107] Rohou A., Grigorieff N. (2015). CTFFIND4: fast and accurate defocus estimation from electron micrographs. J. Struct. Biol..

[108] Wagner T., Merino F., Stabrin M., Moriya T., Antoni C., Apelbaum A., Hagel P., Sitsel O., Raisch T., Prumbaum D. (2019). SPHIRE-crYOLO is a fast and accurate fully automated particle picker for cryo-EM. Commun. Biol..

[109] Shakeel S., Rajendra E., Alcón P., O'Reilly F., Chorev D.S., Maslen S., Degliesposti G., Russo C.J., He S., Hill C.H. (2019). Structure of the Fanconi anaemia monoubiquitin ligase complex. Nature.

[110] Pettersen E.F., Goddard T.D., Huang C.C., Couch G.S., Greenblatt D.M., Meng E.C., Ferrin T.E. (2004). UCSF Chimera--a visualization system for exploratory research and analysis. J. Comput. Chem..

