# Supplementary material for: A direct interaction between CPF and RNA Pol II links RNA 3′ end processing to transcription
Source: Mol Cell. 2023 Dec 21;83(24):4461–4478.e13. doi: 10.1016/j.molcel.2023.11.004 (PMC10783616; doi:10.1016/j.molcel.2023.11.004)
Supplement: Document S1. Figures S1–S12, Table S1, and supplemental references [file mmc1.pdf]

Molecular Cell, Volume *83*

Supplemental information

A direct interaction between CPF and RNA Pol II links RNA 3′ end processing to transcription

Manuel Carminati, Juan B. Rodríguez-Molina, M. Cemre Manav, Dom Bellini, and Lori A. Passmore

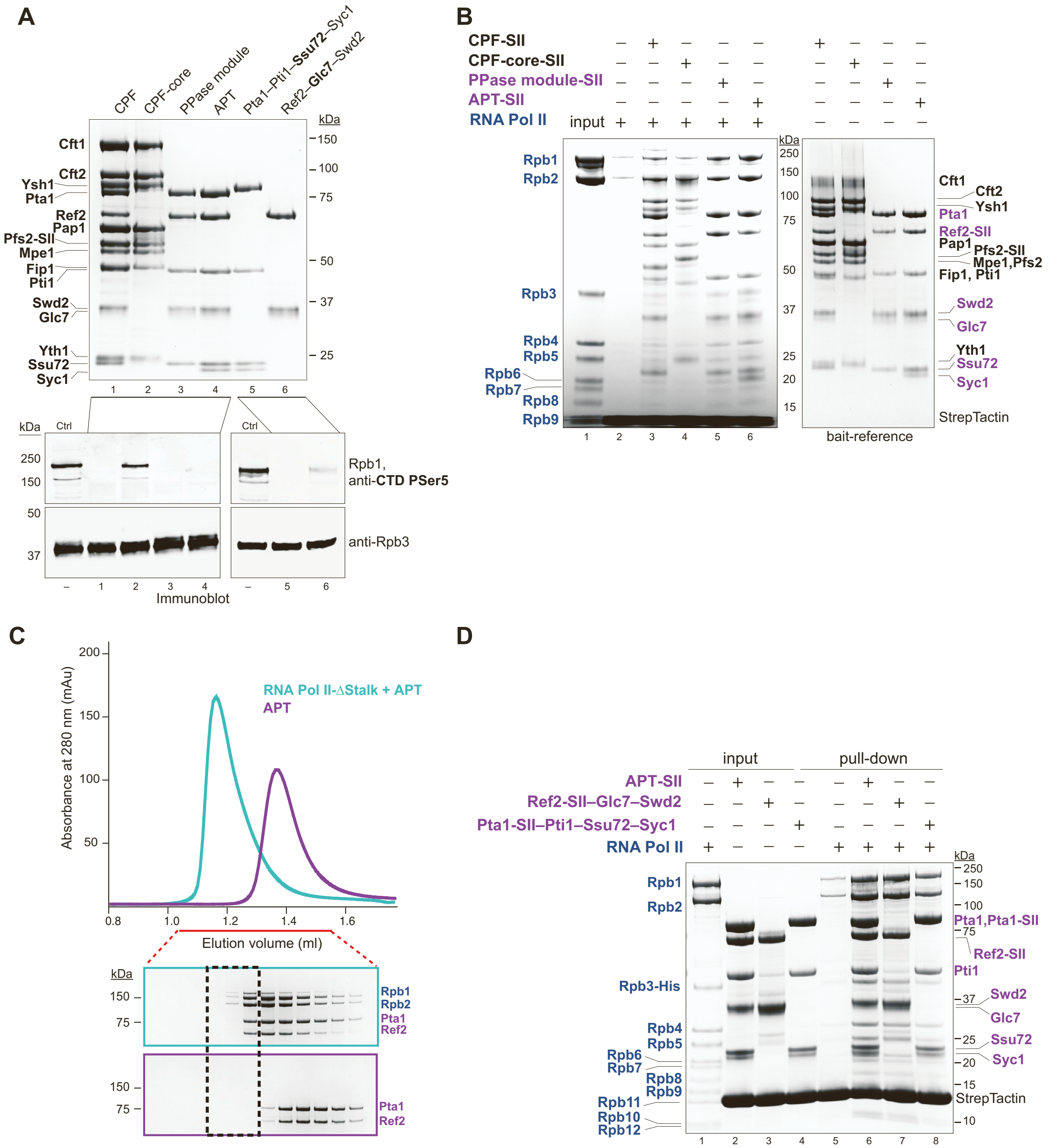

Figure S1 | Characterization of the interaction between CPF and RNA Pol II, Related to Figure 1. (**A**) SDS-PAGE of CPF, APT and CPF subcomplexes. CPF-core includes the nuclease and phosphatase modules; PPase is phosphatase module. The phosphatase module is more prone to degradation *in vitro* compared to APT, likely because APT is a complete complex rather than a subcomplex of CPF. (Bottom) *In vitro* dephosphorylation assay of hyperphosphorylated RNA Pol II with CPF complexes and APT, monitored by immunoblotting with an antibody against phosphorylated Ser5 (P-Ser5) CTD (3E8 antibody). Anti-Rpb3 (1Y26 antibody) was used as a loading control. Dephosphorylation assays against P-Tyr1 were not reproducible for recombinant proteins, possibly because this activity is affected by the CTD phosphorylation pattern, or due to variability in the 3D12 antibody.

(**B**) Pulldown assay of untagged RNA Pol II with SII-tagged CPF, CPF-core, phosphatase module (PPase) or APT immobilized on StrepTactin beads. SII indicates StrepII-tagged proteins. A gel with the bait proteins obtained from a different preparation is shown on the right as reference for comparison with lanes 2-6. RNA Pol II subunits are labelled in blue (left); APT and PPase module subunits are labelled in purple and the remaining CPF subunits are in black (right).
(**C**) Analytical size exclusion chromatography analysis of APT with RNA Pol II-Δstalk. RNA Pol II-Δstalk was purified from a *RPB4* deletion strain. The red line denotes the fractions loaded on the SDS-PAGE below. The dashed black box highlights the migration position of the complex. These data show that the primary binding site for APT is not within the stalk.
(D) Pulldown assay of RNA Pol II using StrepII (SII)-tagged APT (Ref2-SII), Ref2-SII–Glc7–Swd2, or Pta1-SII–Pti1–Ssu72–Syc1 immobilized on StrepTactin beads. Input and bound proteins were analyzed on SDS-PAGE. APT subunits are labelled in purple (right); RNA Pol II subunits are labelled in blue (left). By following the Rpb3-His band, the Ssu72-complex interacts the most weakly with RNA Pol II, compared to APT and the Glc7-subcomplex.

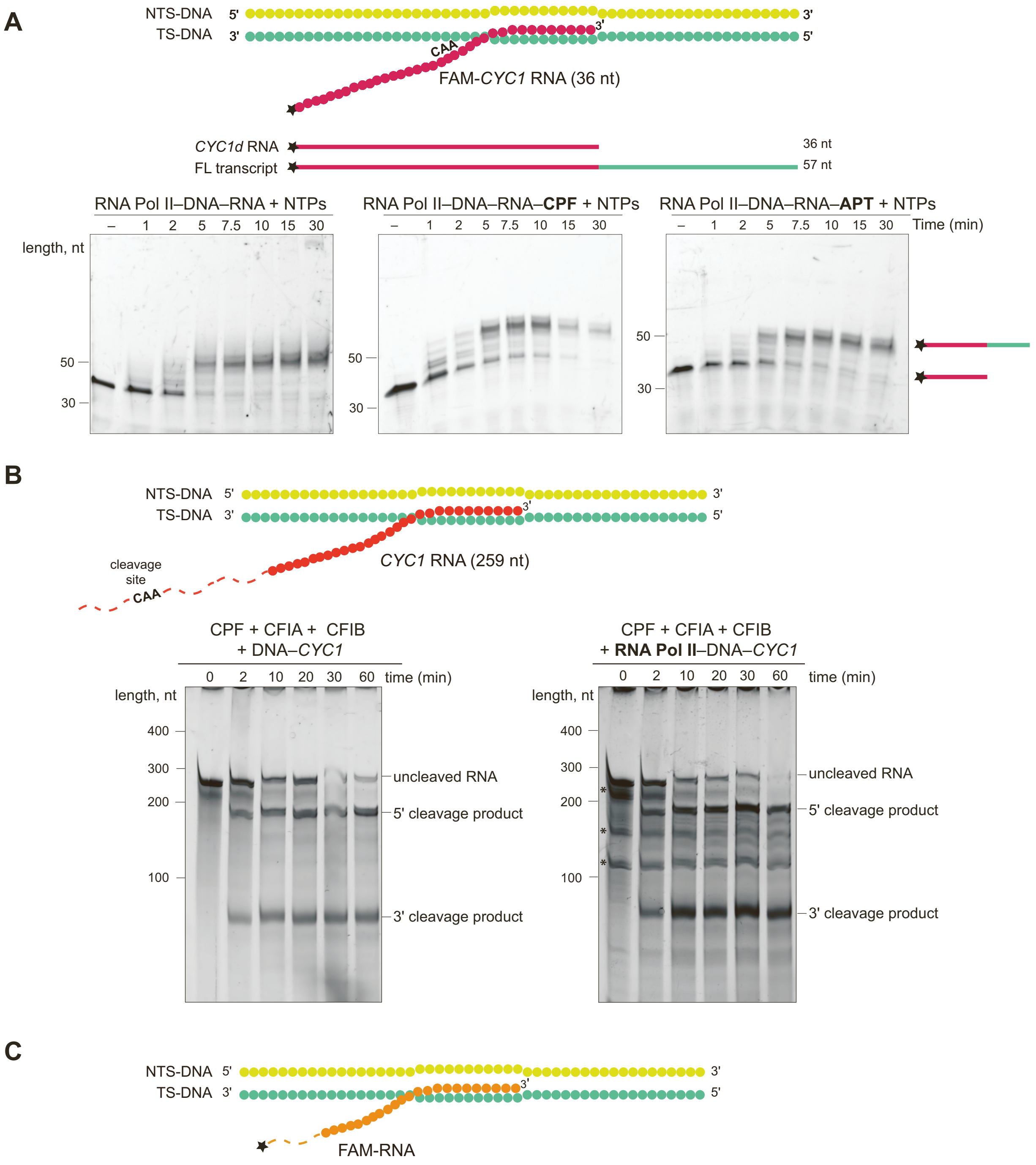

Figure S2 | Functional interactions between CPF or APT and RNA Pol II, Related to Figure 1.
(**A**) Promoter-independent transcription assay where RNA Pol II was incubated with a DNA–RNA scaffold (NTS-DNA_A, TS-DNA_A and *CYC1d*-RNA, Table S1) and a mixture of nucleotide triphosphates (NTPs), with or without APT or CPF. NTS, non-template strand; TS, template strand; FL, full-length; *CYC1*, pre-mRNA substrate containing the CAA cleavage site (*CYC1d* fragment from Hill *et al*., 2019 [S1]). Samples were collected at the indicated time points after the addition of NTPs. RNA extension was monitored on 20% denaturing urea-PAGE. The assays were performed in duplicate.
(**B**) *In vitro* RNA cleavage assays of a 259-nt *CYC1* RNA annealed to the template strand DNA (TS-DNA_B, Table S1) (left) or annealed to an artificial transcription bubble (TS-DNA_B and NTS-DNA_D,

Table S1) assembled with RNA Pol II (right). The CAA cleavage site is depicted on the RNA and is 80 nucleotides away from the annealed sequence. Samples were collected at the indicated time points and cleavage products were analyzed on a 15% denaturing urea-PAGE. The asterisks on the right gel indicate degradation products of the RNA substrate prior to cleavage, likely resulting from the assembly procedure. The assays were repeated three times, and representative gels are shown.
(**C**) Cartoon of the DNA–RNA hybrid mounted on RNA Pol II for cryoEM analysis in complex with APT (TS-DNA_C, NTS-DNA_C and snR47 RNA, Table S1) or Ref2–Glc7–Swd2 (TS-DNA_D, NTS-DNA_D and RNA_D, Table S1). The latter RNA was based on Ehara et al., 2017 [S2].

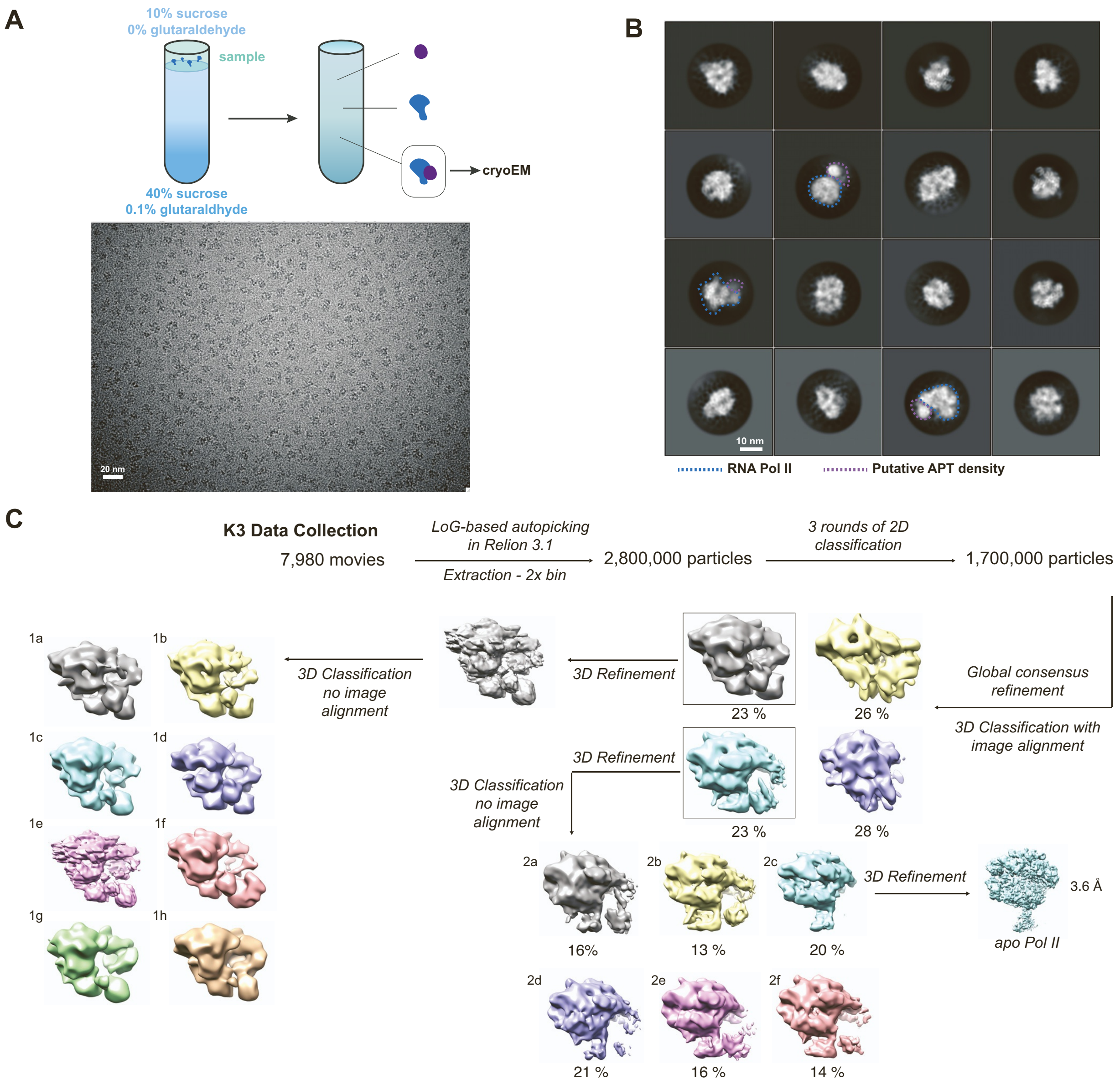

Figure S3 | CryoEM analysis of DNA–RNA-loaded RNA Pol II–APT, Related to Figure 2.

(**A**) (Top) Schematic of the GraFix protocol used for the crosslinking of RNA Pol II–APT on a DNA–RNA scaffold. The gradient of sucrose and glutaraldehyde are indicated. The RNA Pol II–APT mixture was applied on the top of the gradient (left). Blue and purple cartoons are RNA Pol II and APT, respectively. The distribution across the gradient of APT, RNA Pol II and their complex that was vitrified on grids (highlighted) is depicted on the right. (Bottom) Representative cryoEM micrograph collected on a Gatan K3 detector at a magnification corresponding to 1.06 Å/pixel.

(**B**) 2D-class averages of the cross-linked RNA Pol II–APT complex. RNA Pol II core is highlighted in blue, and the putative APT density in purple.

(**C**) Processing pipeline for the RNA Pol II–APT data. Percentages represent the number of particles in each 3D-class, relative to the total particles from the previous step. Model 2a is shown in Figure 2A.

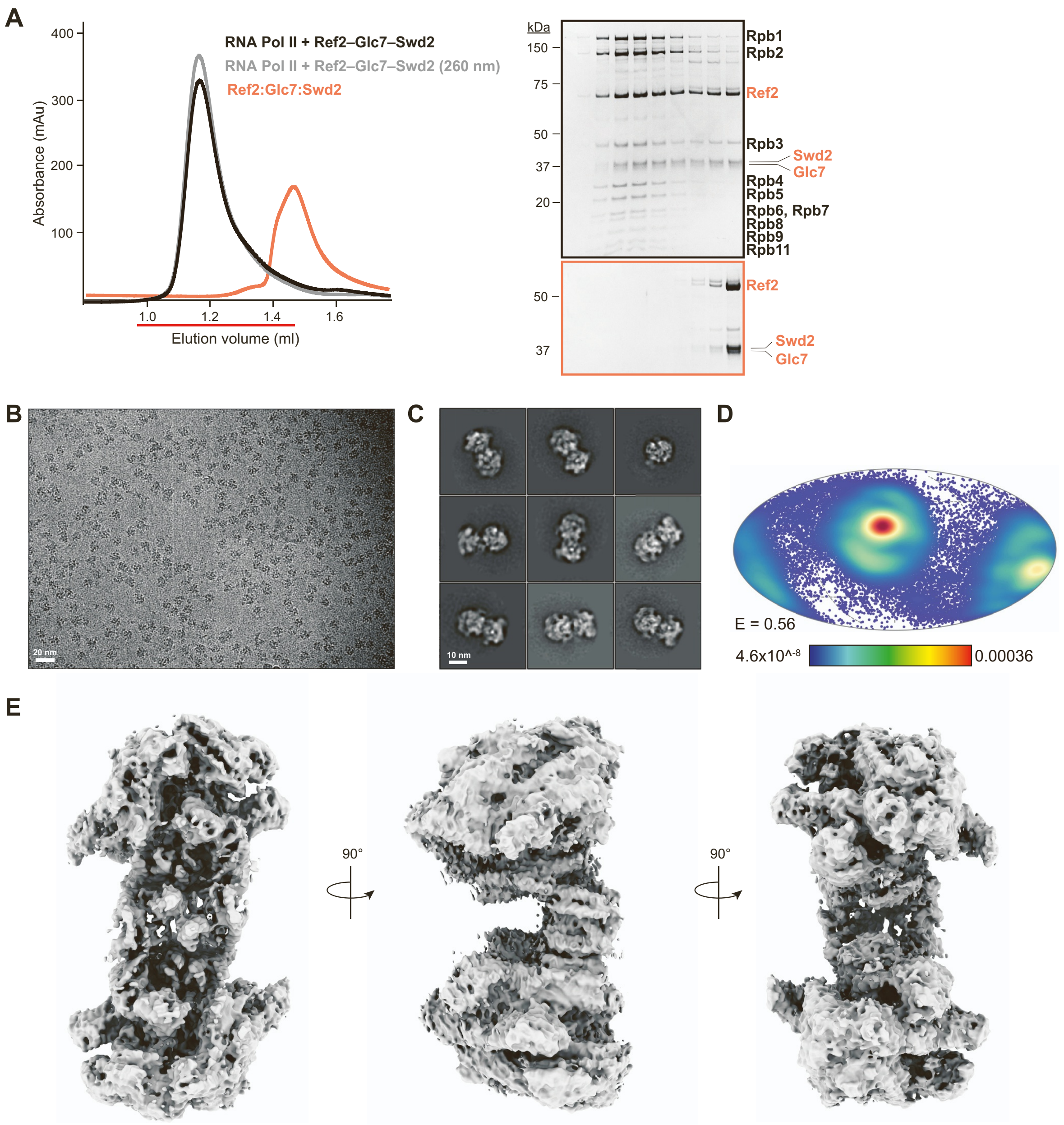

Figure S4 | CryoEM analysis of RNA Pol II bound to Ref2–Glc7–Swd2, Related to Figure 2.

(**A**) Size exclusion chromatography of Ref2–Glc7–Swd2 with RNA Pol II in the presence of a DNA–RNA scaffold. The absorbance is at 280 nm except the grey trace which is at 260 nm. SDS-PAGE of selected fractions is shown on the right. The presence of the nucleic acid scaffold promoted increased association of Ref2–Glc7–Swd2 with Pol II (compare with Figure 1D). This may be due to the presence of an RNA binding region within Ref2 [S3], which might contribute to recognition of RNA.

(**B**) Representative cryoEM micrograph of the complex prepared under native conditions (not crosslinked). Data were collected on a Gatan K3 detector at a magnification corresponding to 0.83 Å/pixel.

(**C**) Selected 2D class-averages from data collected at a 40° tilt angle.

(**D**) Projection plot of orientation distribution of particles contributing to the final RNA Pol II map shown in Figure 2D and Figure S4E. The red to blue color range denotes highest to lowest number of particles. E = cryoEF efficiency [S4].

(**E**) 3D reconstruction of the intact RNA Pol II homodimer before focused refinement.

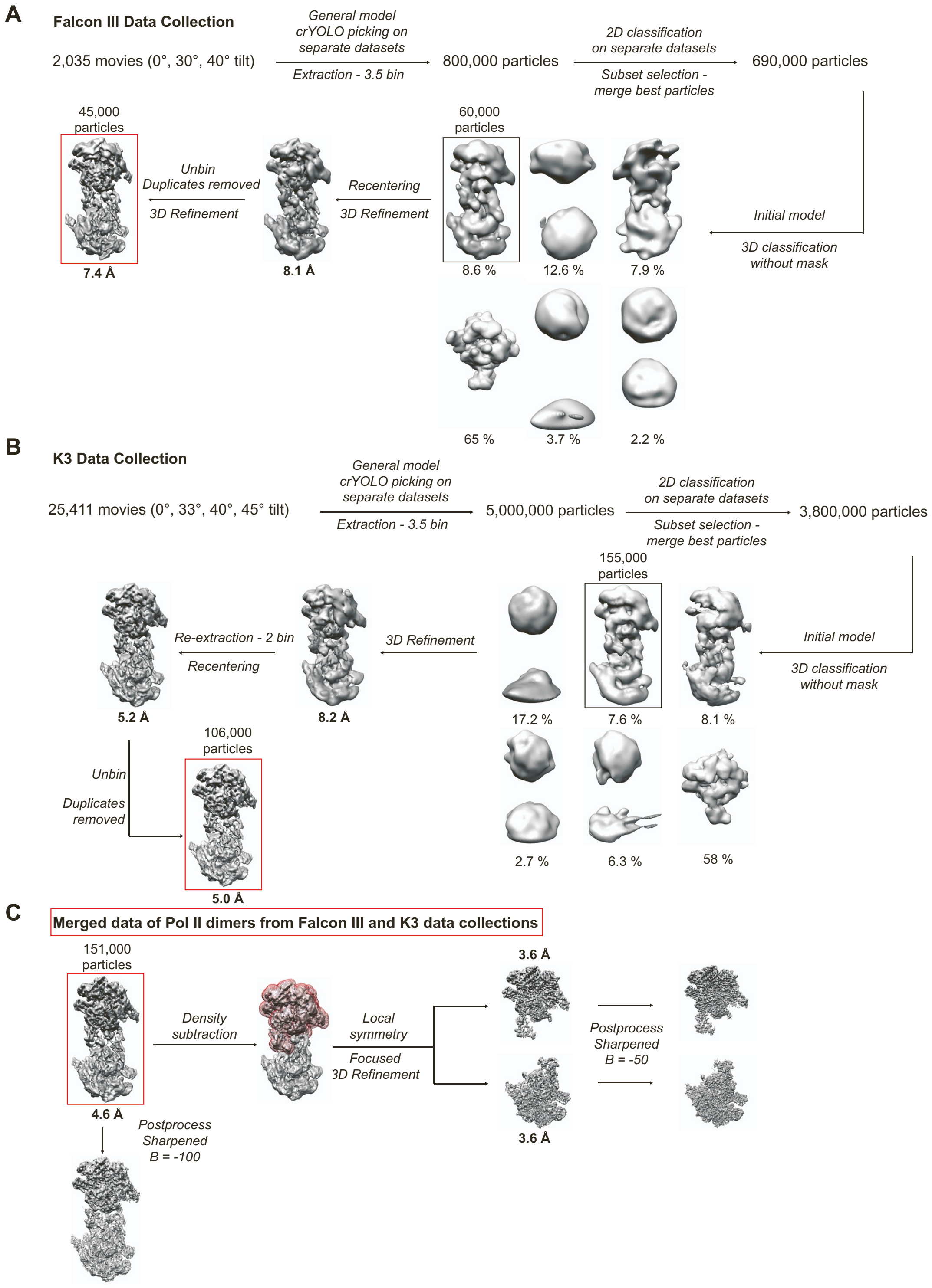

Figure S5 | CryoEM processing pipeline of RNA Pol II with Ref2–Glc7–Swd2, Related to Figure 2. (**A-B**) Flowchart of cryoEM processing for data collected with or without stage tilt on a Falcon III (**A**) or a Gatan K3 (**B**) detector. Percentages represent the number of particles in each 3D-class, relative to the total particles from the previous step. (**C**) Particles from the final 3D reconstructions shown in panels (**A**) and (**B)** were merged and 3D refined (red box). The mask around the top monomer of the RNA Pol II dimer map (red mesh) was used for signal subtraction. Maps were flipped on the Z-axis to match the correct handedness before rigid body fitting of two copies of monomeric RNA Pol II (PDB 5C4X) [S5].

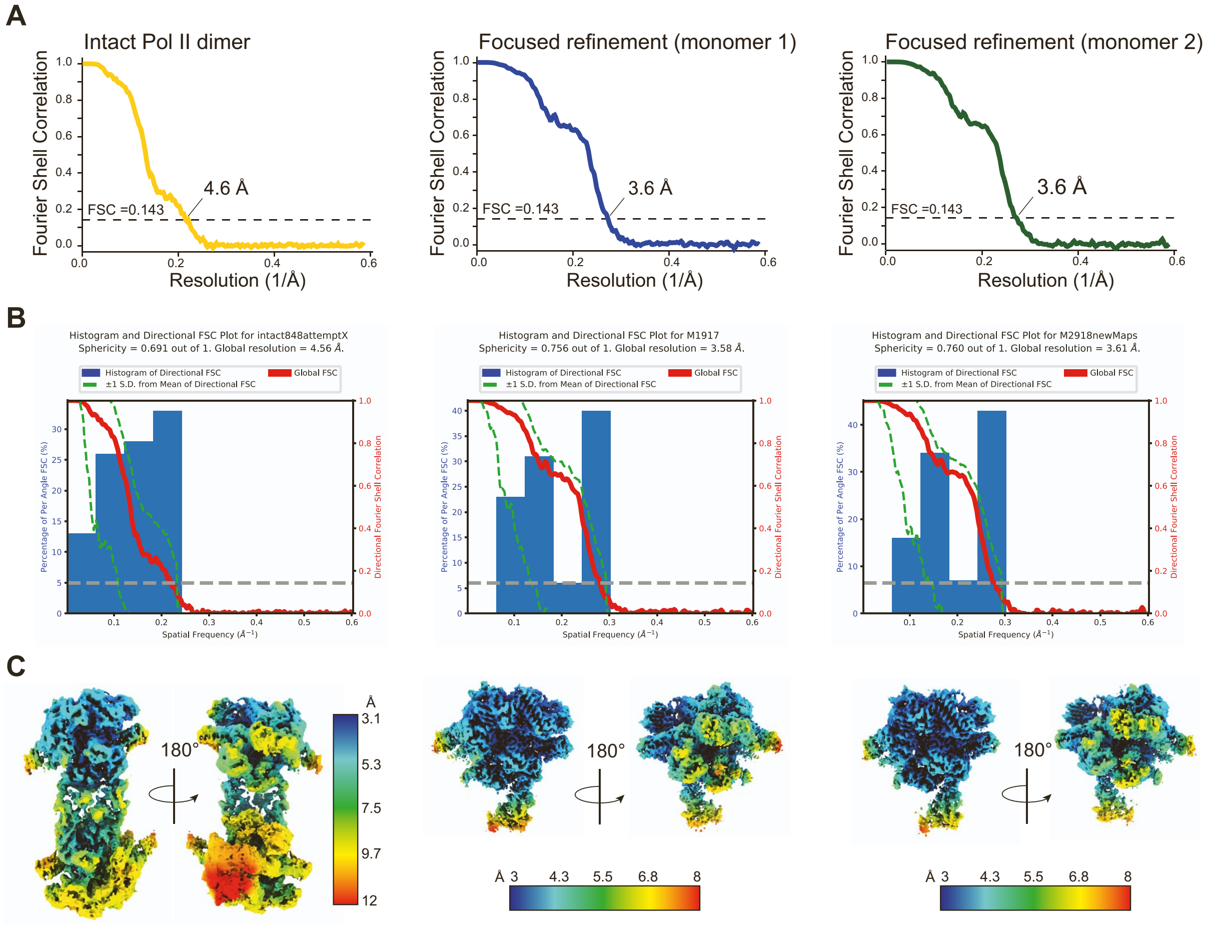

Figure S6 | RNA Pol II dimer cryoEM map validation and local resolution, Related to Figure 2.

(**A**) Fourier-shell correlation (FSC) curves relative to the final reconstructions of the intact RNA Pol II dimer map (left), the focused-refined map of RNA Pol II monomer 1 (middle) or RNA Pol II monomer 2 (right). Resolution was estimated according to the gold-standard FSC at 0.143.
(**B**) Directional FSC plots and sphericity values were calculated via the 3D-FSC server. The intact RNA Pol II dimer maps showed more severe preferred orientations.
(**C**) Local resolutions estimated in Relion (v3.1) and plotted on the final sharpened maps.

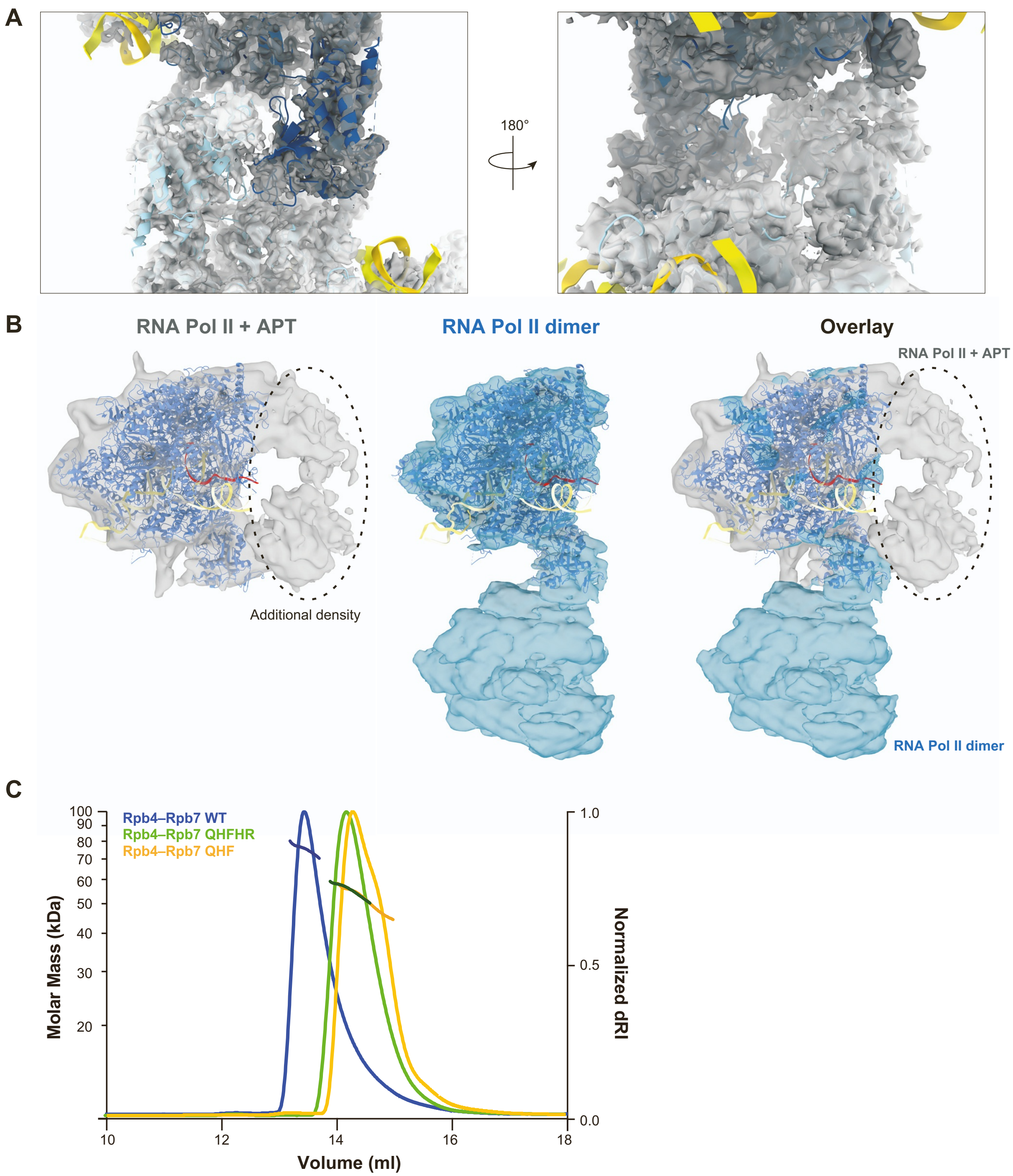

Figure S7 | Validation and biophysics of the RNA Pol II dimer interface, Related to Figure 3.

(**A**) Close-up view of the RNA Pol II dimerization interface. The EM density is displayed as a transparent surface (grey). Fitted monomers 1 and 2 are shown in blue and cyan cartoon, respectively. The DNA–RNA hybrid is in yellow. The resolution of the stalk was not sufficient to confidently re-model it.

(**B**) Comparison and overlay between the cryoEM map of crosslinked RNA Pol II–APT (grey) and of the RNA Pol II dimer (blue). The dashed area indicates the putative APT density, which does not clash with the second monomer of the RNA Pol II dimer.

(**C**) SEC-MALS analysis of recombinant Rpb4–Rpb7 Pol II stalk variants. Samples were run at a concentration of 2 mg/ml and included the wild-type stalk (blue trace), the mutant stalk utilized in the

cryoEM studies in Figure 3F (green), and the stalk carrying the 'QHF' mutations introduced *in vivo* (yellow trace). The traces represent the differential refractive index (dRI) of the samples, normalized to 1 and plotted against the y-axis on the right. The calculated molecular weights across the traces are shown in color-matched solid lines plotted against the left y-axis as 10^3 g/mol (kDa). The lower average molecular mass of the Rpb4–Rpb7 mutants is consistent with a shift towards a monomeric species in a concentration-dependent monomer-dimer equilibrium.

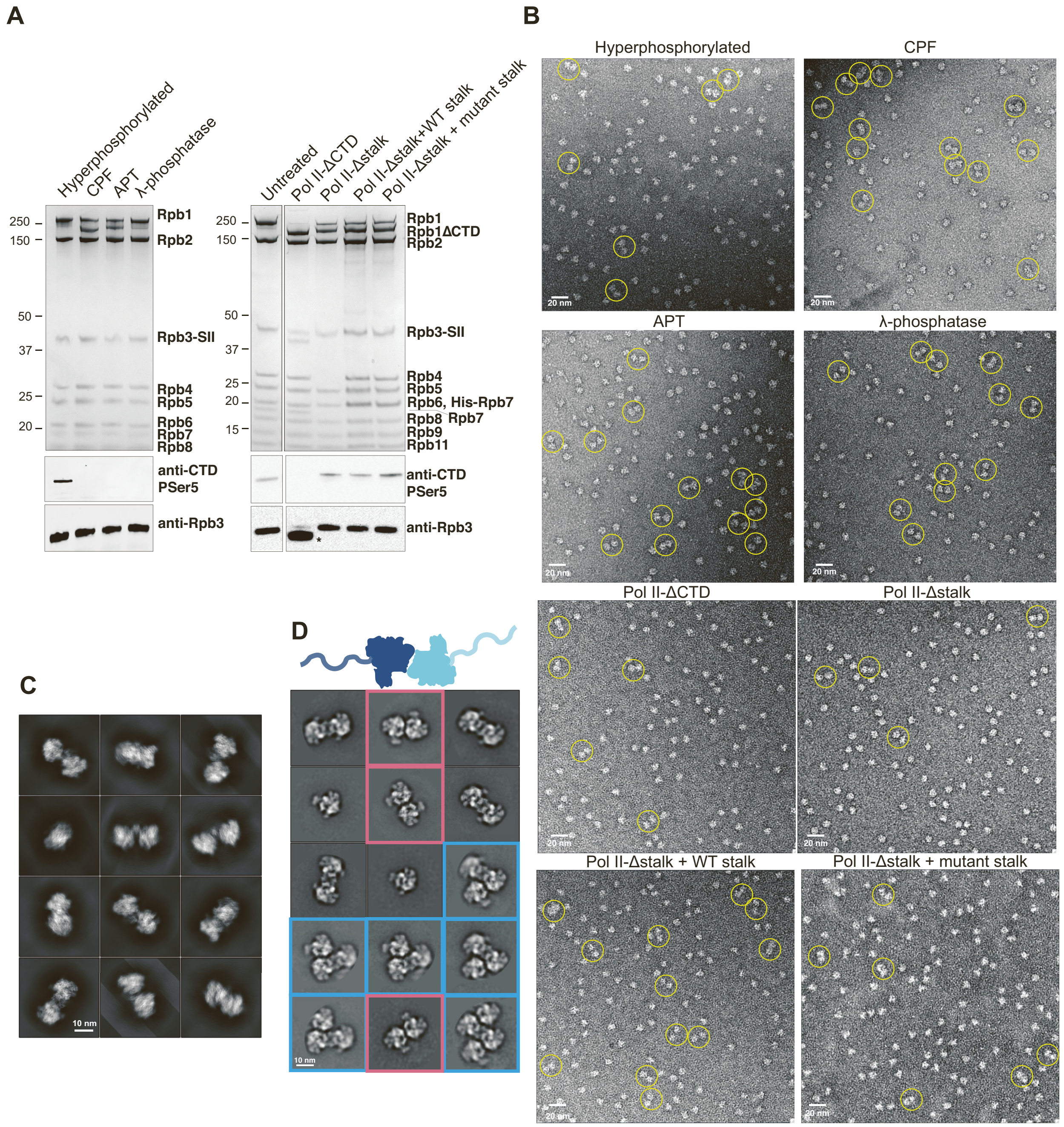

Figure S8 | Negative Stain EM analysis of RNA Pol II dimerization, Related to Figure 4.

(**A**) (Top) SDS-PAGE of the RNA Pol II samples after different treatments used for the negative stain EM shown in panel B, analyzed in Figure 4C. (Bottom) Immunoblots against phosphorylated CTD Ser5 (antibody 3E8) to monitor the phosphorylation state for each condition. Anti-Rpb3 (antibody 1Y26) was used as a loading control. The shifted band marked with a black asterisk most likely resulted from non-specific recognition of a TEV cleavage site (within the Rpb3 tag) by 3C protease.

(**B**) Representative negative stain EM micrographs of the various RNA Pol II samples collected at a magnification of 21,000 X (2.53 Å/pixel). Data from 50-60 micrographs per condition were analyzed in Figure 4C. Particles counted as 'dimeric' RNA Pol II are indicated with a yellow circle.

(**C**) 2D projections of the RNA Pol II homodimer cryoEM structure. The projections were used as a guide for manual inspection of 'dimeric' particles picked by Relion on the micrographs in **B**.

(**D**) Selected cryoEM 2D-class averages of RNA Pol II in the absence of a DNA–RNA hybrid. Classes with cleft-to-cleft dimers are highlighted in pink, whereas higher-order oligomers in light blue. The cleft-to-cleft dimer was not evident in the samples assembled on a DNA–RNA hybrid (confirming that the assembled RNA Pol II complex is stable even at the low nanomolar concentrations required for EM).

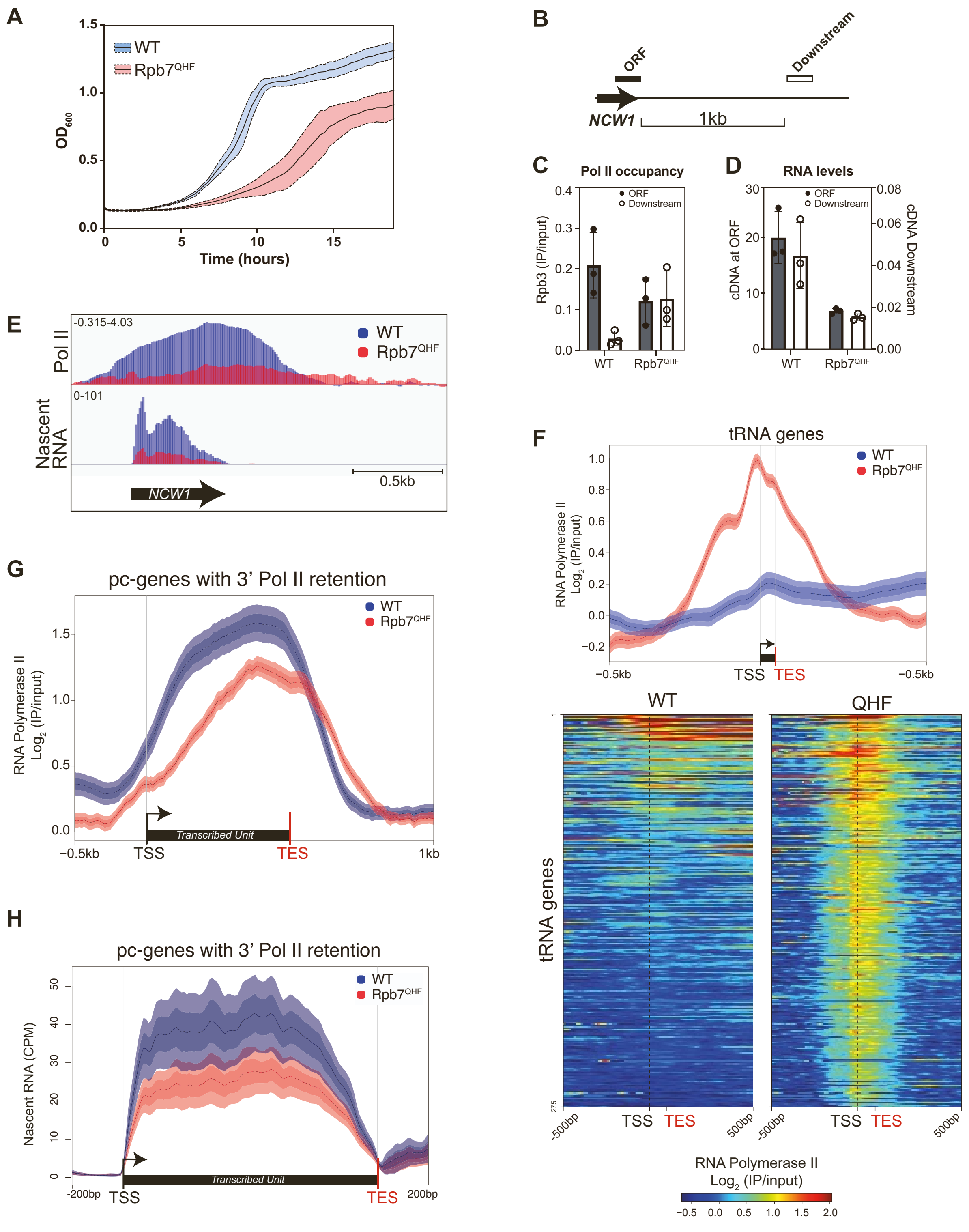

Figure S9 | Characterization of the Rpb7^{QHF} Pol II dimer interface mutant, Related to Figure 4.
(**A**) Growth curve of WT and Rpb7^{QHF} yeast cells. Shaded area corresponds to the standard deviation of the mean (n = 3).
(**B**) Positional schematic of qPCR probes within the ORF and Downstream region of the *NCW1* gene.

(**C**) ChIP-qPCR of Pol II (Rpb3) at the ORF and Downstream region of the *NCW1* gene in wild-type (WT) and $Rpb7^{QHF}$ cells. Values for individual replicates (n = 3) are overlayed with the bar plots which represent the average. Error bars correspond to the standard deviation of the mean. In wild-type cells, RNA Pol II occupancy decreases downstream of the gene body, consistent with transcription termination and release of Pol II. In contrast, in $Rpb7^{QHF}$ cells the downstream RNA Pol II signal is comparable to the signal within the gene body of *NCW1*, indicative of a transcription termination defect.
(**D**) RT-qPCR of total RNA at the ORF and Downstream region of the *NCW1* gene. Note that the values for the Downstream region are plotted on a different scale shown to the right of the bar graph. Values for individual replicates (n =3) are overlayed with the bar plots which represent the average. Error bars correspond to the standard deviation of the mean. Surprisingly, we did not observe a corresponding read-through transcript despite the apparent termination defect in (C).
(**E**) Representative snapshot of the ChIP-seq and 4tU-seq data at the *NCW1* locus. Data from WT and $Rpb7^{QHF}$ cells are overlayed, and are plotted on the same scale (scale shown on the top left). This shows a decrease in RNA Pol II occupancy across gene bodies, as well as retention of RNA Pol II beyond the canonical termination site at some genes.
(**F**) There is a marked increase in RNA Pol II occupancy at tRNA genes in $Rpb7^{QHF}$ (right) compared to WT (left) cells. The physiological relevance of this observation remains unclear. A recent study has shown that a mutation in Sen1 that disrupts its interaction with RNA Pol III, leads to an increase in RNA Pol II occupancy upstream of tRNA genes [S6]. This observation has been interpreted as a role for Sen1 in preventing Pol II-Pol III conflicts at the vicinity of tRNA genes. It is possible that the Rpb7 dimerization surface is required for resolving or preventing Pol II-Pol III conflicts, but further studies are required to test this possibility.
(**G**) Metagene plot of the average RNA Pol II occupancy over the transcribed unit of protein-coding (pc) genes with high RNA Pol II signal (199 genes, see Figure 4E-F). This shows lower overall RNA Pol II occupancy across the transcribed units but RNA Pol II retention beyond the TES in the $Rpb7^{QHF}$ strain. TSS, transcription start site; TES, transcription end site.
(**H**) Metagene plot of average nascent RNA signal over the transcribed unit of pc genes with high RNA Pol II signal (199 genes, see Figure 4E-F) in wild-type and $Rpb7^{QHF}$ cells. CPM; counts per million.

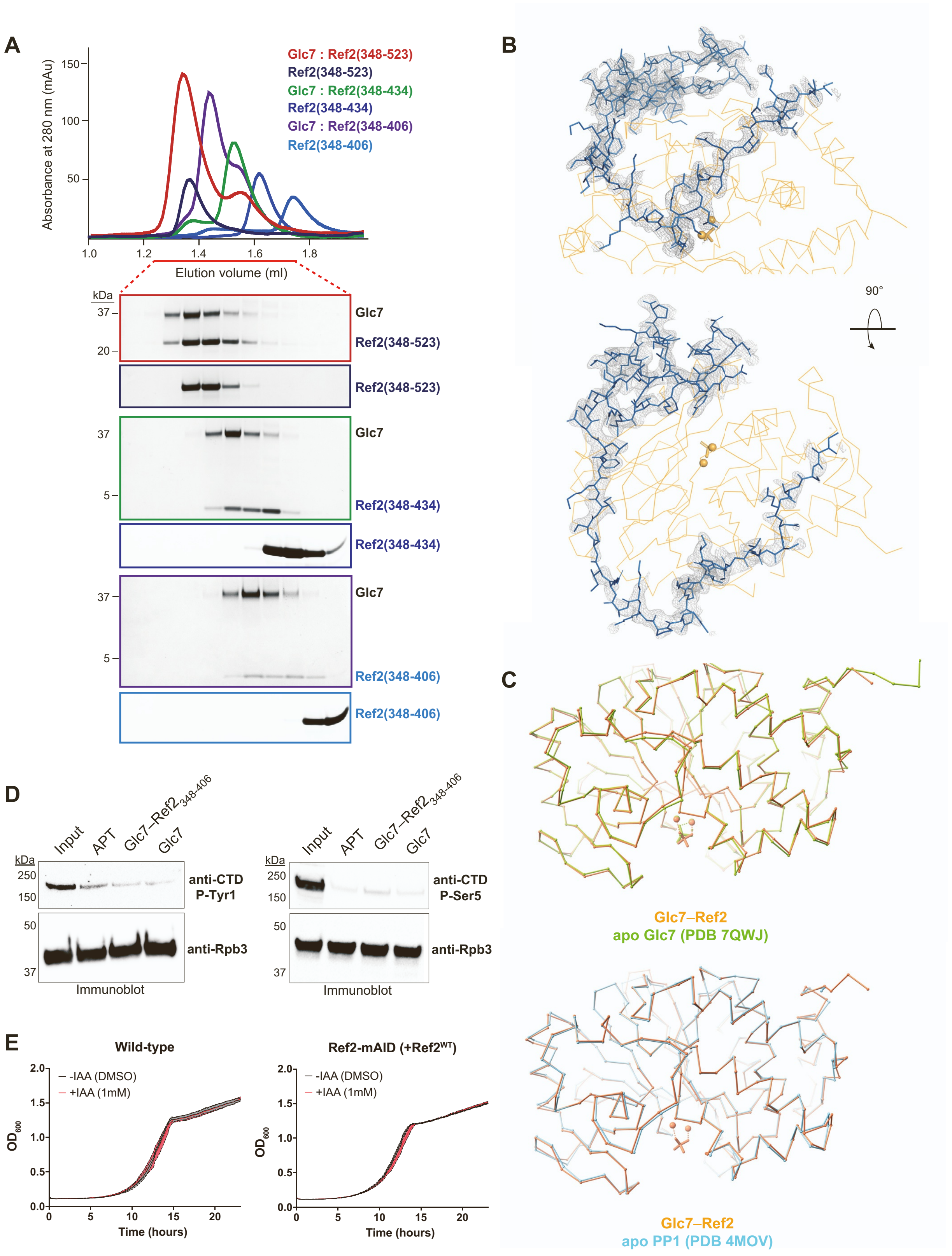
A
Glc7 : Ref2(348-523)
Ref2(348-523)
Glc7 : Ref2(348-434)
Ref2(348-434)
Glc7 : Ref2(348-406)
Ref2(348-406)
Absorbance at 280 nm (mAu)
150
100
50
1.0
1.2
1.4
1.6
1.8
Elution volume (ml)
kDa
37
20
5
Glc7
Ref2(348-523)
Ref2(348-434)
Ref2(348-406)
B
90°
C
Glc7–Ref2
apo Glc7 (PDB 7QWJ)
Glc7–Ref2
apo PP1 (PDB 4MOV)
D
Input
APT
Glc7–Ref2348-406
Glc7
kDa
250
150
50
37
anti-CTD
P-Tyr1
anti-CTD
P-Ser5
anti-Rpb3
Immunoblot
E
Wild-type
Ref2-mAID (+Ref2WT)
-IAA (DMSO)
+IAA (1mM)
OD600
2.0
1.5
1.0
0.5
0.0
0
5
10
15
20
Time (hours)

Figure S10 | Structural analysis of Ref2 binding to Glc7, Related to Figure 5.

(**A**) Identification of the Glc7-binding region on Ref2 (fragments in shades of blue) by size exclusion chromatography. The elution profiles of the Ref2 truncations in isolation or in complex with Glc7 were overlayed, and the peak fractions were analyzed by SDS-PAGE. The outlines of the gels correspond to the colors of the chromatogram traces.

(**B**) Two views of the $Ref2_{348-406}$ peptide (blue sticks) built into the electron density obtained from X-ray crystallography. The grey mesh is an F_o-F_c difference map displayed at 1.5 σ contour level. Glc7 is represented in ribbon (orange).

(**C**) Overlay of the Ref2–Glc7 crystal structure (orange) with apo-Glc7 (green; PDB 7QWJ), and apo-PP1 (cyan; PDB 4MOV) [S7]. The Ref2 peptide was omitted for clarity.

(**D**) *In vitro* dephosphorylation assay of hyperphosphorylated RNA Pol II comparing the activity of APT and isolated Glc7 to a Glc7–$Ref2_{348-406}$ chimera lacking the CTD peptide. Immunoblots were performed with antibodies that recognize phosphorylated CTD Tyr1 (top) and phosphorylated CTD Ser5 (bottom) (antibodies 3D12 and 3E8, respectively). Anti-Rpb3 (1Y26) was used as loading control.

(**E**) Growth curves of the wild-type Ref2-mAID parent strain (left) and Ref2-mAID cells co-expressing a wild-type copy of Ref2 (right). Cells were grown in 1 mM auxin (IAA) or an equivalent volume of DMSO (solvent control). Dotted line represents the average OD_{600} of biological replicates (n = 3), and the shaded area represents the standard deviation of the mean. Growth arrest of Ref2-mAID is dependent on the mAID tag (left) and can be rescued by co-expression of wild-type *REF2* (right).

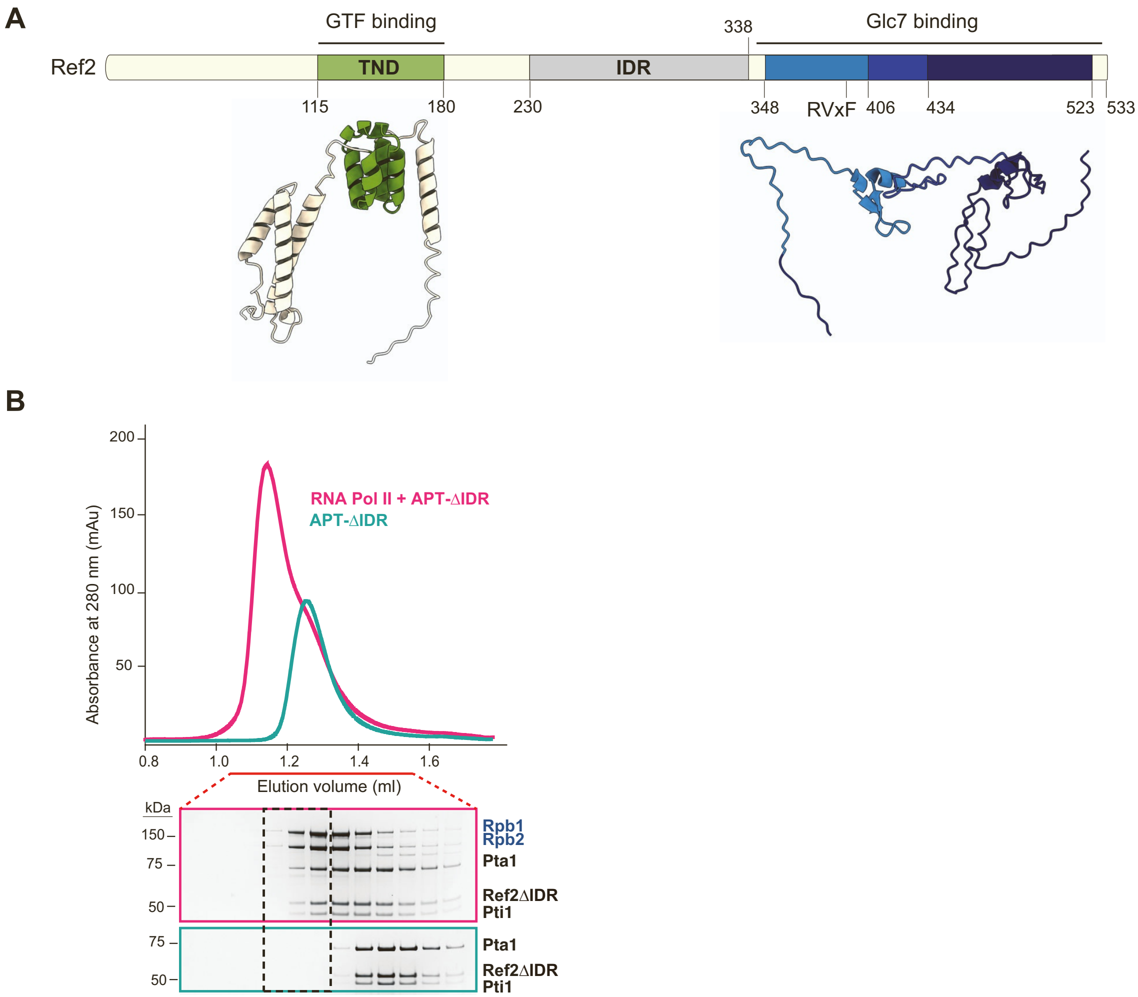

Figure S11 | Characterization of Ref2-mediated interaction with RNA Pol II, Related to Figure 6.

(**A**) Domain diagram of yeast Ref2 (Uniprot P42073) with the AlphaFold2 structure prediction shown below in cartoon. The N-terminal region harbors a TFIIS N-terminal domain (TND) (green). A predicted intrinsically-disordered region (IDR) (grey) spans more than 100 residues in the middle of Ref2. The C-terminal region contains an RVxF motif that has been proposed to bind Glc7 *in vivo* [S8].The different shades of blue in the schematic and cartoon representation show the three fragments that were tested for Glc7 binding in Figure S10A.

(**B**) Size exclusion chromatography of RNA Pol II in complex with APT-ΔIDR. The elution profile of the complex is in magenta, and the fractions indicated with a red line were loaded on SDS-PAGE. The black dashed line indicates the complex being formed. The outlines of the gels correspond to the colors of the chromatogram traces.

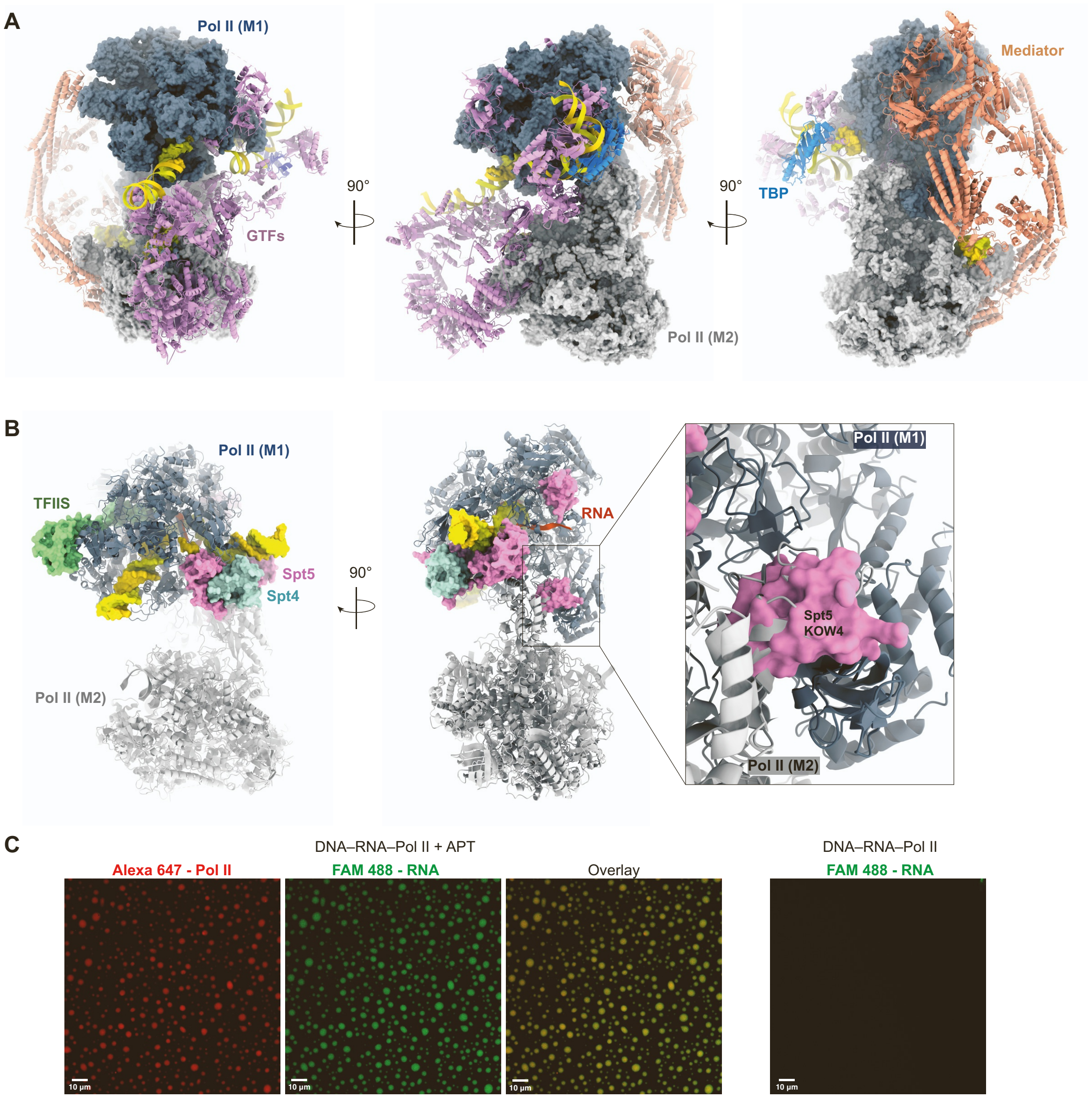

Figure S12 | Overlay of RNA Pol II dimer with other transcription complexes, Related to Figure 7.

(**A**) Views of the structural superposition between the yeast RNA Pol II dimer (monomer 1 and monomer 2 in grey surface) and the pre-initiation complex (PIC) (PDB 5OQM) [S9] in cartoon. Mediator subunits are shown in salmon, TATA-binding protein (TBP) in blue, general transcription factors (GTFs) in pink, and DNA is in yellow. Monomer 2 has major clashes with mediator and GTF subunits including TFIIB, TFIIE and TFIIH.

(**B**) Superposition of dimeric yeast RNA Pol II (in cartoon) with the elongation complex (EC) shown in surface (PDB 5XON) [S2]. EC RNA Pol II has been omitted from the figure for clarity. TFIIS is in green, Spt5 in pink and Spt4 in cyan. The inset on the right shows a clash between monomer 2 from the dimer and the KOW4 domain of Spt5.

(**C**) Confocal microscopy images of 0.6 µM RNA Pol II - Alexa Fluor 647 loaded with a DNA–RNA(FAM) scaffold [S2] (Figure S2C) in the presence or in the absence of 4 µM APT. The overlay shows the co-localization of RNA Pol II with the loaded DNA–RNA scaffold. The control on the right is RNA Pol II mounted with the DNA–RNA hybrid without APT. Condensate formation can only be observed in the presence of APT. The experiment was repeated twice, and representative confocal acquisitions are shown.

Table S1 | List of DNA and RNA oligonucleotides used across the study, Related to Key Resources Table

Purpose	Name	Sequence (5′-3′)	Source
CRISPR Rpb1-3C-CTD	Donor 1	GGTGCATTTGATGTGATGATCGATGAGGAGTCACTGGTAAAATACATGTTGGAAGTTTTATTCCAAGGTCCAGAACAAAAAAATAACTGAGATTGAAGACGGACAAGATGGTGGCGTCACACCATACAGTAACGAAAGTGGTTTGGTCAATGCAGATCTTGACGTTAAAGATGAGCTAATGTTTTCACCTTTGGTAGATTCTGGTTCTAATGACGCTATGGCTGGAGGATTTACAGCGTACGGTGGTGCTGATTATGGTG	This study
CRISPR Rpb1-3C-CTD	sgRNA1	TTTGAACCCGAATCAACCAGAGG	CRISPOR
CRISPR Rpb3-His$_6$-3C-SII	Donor 9	ATGCATCTCAAATGGGTAATACTGGATCAGGAGGGTATGATAATGCTTGGgaaaacctgtattttcagggggggtagtcatcaccatcaccatcacggtagtTGGTCACATCCGCAGTTTGAAAAAGGTAGCGCAGGTAGTGCAGCAGGTAGCGGTGCAGGTTGGAGCCATCCTCAGTTTGAGAAAtagTTCTCAAGTGGGCGTAATAGAGGAAAAAAAACAAGTGAACGAACCGAAAATA	This study
CRISPR Rpb3-His$_6$-3C-SII	sgRNA 67_fw	AATGCTTGGTAGTTCTCAAG	CRISPOR
CRISPR Ref2-mAID	sgRNA 56R_top	GATCCATGTCATAGCTTAGTGTACGTTTTAGAGCTAG	CRISPOR
CRISPR Ref2-mAID	sgRNA 6R_bottom	CTAGCTCTAAAACGTACACTAAGCTATGACATG	CRISPOR
C-term mAID Ref2 donor	Fwd	AGCATGTCCCCATAGTAAAAAGAAATAAATATCCTCCAAGAGGAGTACACCGGATCCCCgggTTAATTAA	This study
C-term mAID Ref2 donor	Rev	GAAGCTTGCAAGGCATGAGAAATATTTACTAATTGAGGAACAGGTGCTGA TTTATCATCATCATCTTTATAATCC	This study
Native REF2 cassette	Ref2p_pRS_Fwd	cggccgctctagaactagtggatccTGTATCACGTCGGCACACCAACGGTTTC	This study
Native REF2 cassette	Ref2t_pRS_Rev	cgaattcctgcagcccgggggatccGGAGTATTTCCCACCTTTTGGGGCC	This study
Ref2^{mut} mutagenesis	Ref2_I372D_F374K_Y384E_Fwd	GGTAAACCGAAAAAAGCACGCATAAGTAGCGATAAAAAATTGGATGATTCCCAACTAATAAAAGTTGAAGGTGACGATCTACCGAACCAAGGGCTACAA	This study
Ref2^{mut} mutagenesis	Ref2_I372D_F374K_Y384E_Rev	TTGTAGCCCTTGGTTCGGTAGATCGTCACCTTCAACTTTTATTAGTTGGGAATCATCCAATTTTTTATCGCTACTTATGCGTGCTTTTTTCGGTTTACC	This study
CRISPR Rpb7^{QHF}	Donor 17	CTGTAGTTTTCAAACCATTTAAAGGGGAAGTAGTGGACGGCACAGTCGTTTCATGTTCTGCGGCCGGaTTCGAAGTGCAAGTAGGTCCAATGAAAGTAAAGGTGACAAAGCATCTGATGCCTCAAGATTTAACCTTTAATGCGGGTTCAAACCCACCATCATACCAAAGTTCCGAGGATGTCATCACCATAAAAAGTAGAATTAGAGTTAAAATTGAAGGTTGTATCAGTCAAGTGAGTTCTATTGCCGCAATCGaTAGTATCAAAGAAGATTATTTGGGTGCTATTTAATCACTTGTTAC	This study
CRISPR Rpb7^{QHF}	R7_15	GATCTCGTTTCATGTTCTCAGCACGTTTTAGAGCTAG	This study
CRISPR Rpb7^{QHF}	R7_16	CTAGCTCTAAAACGTGCTGAGAACATGAAACGA	This study
qPCR	NCW1_ORF_Fwd	TCTACTGCTAGTTCCAGTGCCGCTAAG	[S10]
qPCR	NCW1_ORF_Rev	TTAAATACCGGTGCCTAGGACGAAAG	[S10]
qPCR	S_pombe_gpd3_+892_F	TCTGCCGGTATCCAACTTTC	[S10]
qPCR	S_pombe_gpd3_+892_R	TCAACAACACGGTGGGAGTA	[S10]
qPCR	S_pombe_act1_+111_F	ACCCCGTCACCATGGTATTA	[S10]
qPCR	S_pombe_act1_+186_R	ACGCTTGCTTTGAGCTTCAT	[S10]

qPCR	NCW1_downstream_Fwd	TCCATGGGAATAACGATGCACTT	[S10]
qPCR	NCW1_downstream_Rev	ACAGAGTAGATTCGTAATAACTTAAATGGC	[S10]
Transcription assay	TS-DNA_A	CACTCTACCGATAAGCAGACGTACCTCTCGACCCTGTGCTAGACACGG	This study
Transcription assay	NTS-DNA_A	CCGTGTCTAGCACAGGGAAATGGTTTGTGTCTGCTTATCGGTAGAGTG	This study
RNA cleavage assay	TS-DNA_B	CACTCTACCGATAAGCAGACGTTTCCCAAAACCCTGTGCTAGACACGG	This study
Electron microscopy	TS-DNA_C	CCTACCGATAAGCAGACGATATCAGTGTACCACGGACTCTTTATATACAAGCG	This study
Electron microscopy	NTS-DNA_C	CGCTTGTATATAAAGAGTCCGTGGATGTGACTATAGCAGTGCTTATCGGTAGG	This study
Electron microscopy	TS-DNA_D	CACTCTACCGATAAGCAGACGTACCTCTCGACCCTGTGCTAGACACGG	[S2]
Electron microscopy/ RNA cleavage assay	NTS-DNA_D	CCGTGTCTAGCACAGGGAAATGGTTTGTGTCTGCTTATCGGTAGAGTG	[S2]
RNA transcription assay	*CYC1d* RNA	AAGAACGUUAUUUAUAUUU**CAA**AUUUUUCUUUUUUU	[S1]
RNA cleavage assay	Full length *CYC1* RNA	GGGAGAACAGGCCCCUUUUCCUUUGUCGAUAUCAUGUAAUUAGUUAUGUCACGCUUACAUUCACGCCCUCCUCCCACAUCCGCUCUAACCGAAAAGGAAGGAGUUAGACAACCUGAAGUCUAGGUCCCUAUUUAUUUUUUUUAUAGUUAUGUUAGUAUUAAGAACGUUAUUUAUAUUUCAAAUUUUUCUUUUUUUUCUGUACAAACGCGUGUACGCAUGUAACAUUAUACUGAAAACCUUGCUUGAGAAGGUUUUGGGA	[S11]
Electron microscopy	*snR47* RNA	FAM-AUAUAUGAUGAUAUCCUAUAACAACAACAACAUGAAUUUCUUCGUCCGAAUCCUUUAUAGGUGGAAACAAACUUUGACAAUAGCUUUUUAACACUGAUA	This study
Electron microscopy	RNA_D	FAM-AUCUUGAAUCUAUUUCUUUUAUCGAGAGGU	[S2]

Supplemental references

S1. Hill, C.H., Boreikaite, V., Kumar, A., Casanal, A., Kubik, P., Degliesposti, G., Maslen, S., Mariani, A., von Loeffelholz, O., Girbig, M., et al. (2019). Activation of the Endonuclease that Defines mRNA 3' Ends Requires Incorporation into an 8-Subunit Core Cleavage and Polyadenylation Factor Complex. Mol Cell *73*, 1217-1231 e1211. 10.1016/j.molcel.2018.12.023.

S2. Ehara, H., Yokoyama, T., Shigematsu, H., Yokoyama, S., Shirouzu, M., and Sekine, S.I. (2017). Structure of the complete elongation complex of RNA polymerase II with basal factors. Science *357*, 921-924. 10.1126/science.aan8552.

S3. Russnak, R., Nehrke, K.W., and Platt, T. (1995). REF2 encodes an RNA-binding protein directly involved in yeast mRNA 3'-end formation. Mol Cell Biol *15*, 1689-1697. 10.1128/MCB.15.3.1689.

S4. Naydenova, K. and Russo, C.J. (2017) Measuring the effects of particle orientation to improve the efficiency of electron cryomicroscopy. Nat Commun *8*, 629. 10.1038/s41467-017-00782-3

S5. Barnes, C.O., Calero, M., Malik, I., Graham, B.W., Spahr, H., Lin, G., Cohen, A.E., Brown, I.S., Zhang, Q., Pullara, F., et al. (2015). Crystal Structure of a Transcribing RNA Polymerase II Complex Reveals a Complete Transcription Bubble. Mol Cell *59*, 258-269. 10.1016/j.molcel.2015.06.034.

S6. Aiello, U., Challal, D., Wentzinger, G., Lengronne, A., Appanah, R., Pasero, P., Palancade, B., and Libri, D. (2022). Sen1 is a key regulator of transcription-driven conflicts. Mol Cell *82*, 2952-2966. 10.1016/j.molcel.2022.06.021

S7. Choy, M.S., Hieke, M., Kumar, G.S., Lewis, G.R., Gonzalez-DeWhitt, K.R., Kessler, R.P., Stein, B.J., Hessenberger, M., Nairn, A.C., Peti, W., and Page, R. (2014). Understanding the antagonism of retinoblastoma protein dephosphorylation by PNUTS provides insights into the PP1 regulatory code. Proc Natl Acad Sci U S A *111*, 4097-4102. 10.1073/pnas.1317395111.

S8. Nedea, E., Nalbant, D., Xia, D., Theoharis, N.T., Suter, B., Richardson, C.J., Tatchell, K., Kislinger, T., Greenblatt, J.F., and Nagy, P.L. (2008). The Glc7 phosphatase subunit of the cleavage and polyadenylation factor is essential for transcription termination on snoRNA genes. Mol Cell *29*, 577-587. 10.1016/j.molcel.2007.12.031.

S9. Schilbach, S., Aibara, S., Dienemann, C., Grabbe, F., and Cramer, P. (2021). Structure of RNA polymerase II pre-initiation complex at 2.9 A defines initial DNA opening. Cell *184*, 4064-4072 e4028. 10.1016/j.cell.2021.05.012.

S10. Rodriguez-Molina, J.B., O'Reilly, F.J., Fagarasan, H., Sheekey, E., Maslen, S., Skehel, J.M., Rappsilber, J., and Passmore, L.A. (2022). Mpe1 senses the binding of pre-mRNA and controls 3' end processing by CPF. Mol Cell *82*, 2490-2504 e2412. 10.1016/j.molcel.2022.04.021.

S11. Casanal, A., Kumar, A., Hill, C.H., Easter, A.D., Emsley, P., Degliesposti, G., Gordiyenko, Y., Santhanam, B., Wolf, J., Wiederhold, K., et al. (2017). Architecture of eukaryotic mRNA 3'-end processing machinery. Science *358*, 1056-1059. 10.1126/science.aao6535.
